# Cancer genome standards for long-read sequencing using cancer cell line mixtures

**DOI:** 10.1093/gigascience/giag037

**Published:** 2026-04-03

**Authors:** Jia Zhang, Ho Yi Wong, Lingchen Liu, Lambros T Koufariotis, Scott Wood, Nadine Fitzpatrick, Jenny Quiatchon, Paul Collins, John V Pearson, Nicola Waddell

**Affiliations:** QIMR Berghofer Medical Research Institute, Herston, Brisbane, QLD, 4006, Australia; Faculty of Health, Medicine and Behavioural Sciences, The University of Queensland, Brisbane, QLD, 4102, Australia; QIMR Berghofer Medical Research Institute, Herston, Brisbane, QLD, 4006, Australia; Faculty of Health, Medicine and Behavioural Sciences, The University of Queensland, Brisbane, QLD, 4102, Australia; QIMR Berghofer Medical Research Institute, Herston, Brisbane, QLD, 4006, Australia; Faculty of Health, Medicine and Behavioural Sciences, The University of Queensland, Brisbane, QLD, 4102, Australia; QIMR Berghofer Medical Research Institute, Herston, Brisbane, QLD, 4006, Australia; QIMR Berghofer Medical Research Institute, Herston, Brisbane, QLD, 4006, Australia; QIMR Berghofer Medical Research Institute, Herston, Brisbane, QLD, 4006, Australia; QIMR Berghofer Medical Research Institute, Herston, Brisbane, QLD, 4006, Australia; QIMR Berghofer Medical Research Institute, Herston, Brisbane, QLD, 4006, Australia; QIMR Berghofer Medical Research Institute, Herston, Brisbane, QLD, 4006, Australia; QIMR Berghofer Medical Research Institute, Herston, Brisbane, QLD, 4006, Australia; Faculty of Health, Medicine and Behavioural Sciences, The University of Queensland, Brisbane, QLD, 4102, Australia

**Keywords:** long-read sequencing, Oxford Nanopore Technologies, somatic mutation, tumour purity, sequencing depth

## Abstract

**Background:**

Long-read sequencing (LRS) improves genome alignment and facilitates resolving variants in genomic regions of low complexity, making it a promising approach for cancer variant detection and biomarker discovery.

**Results:**

Here, we evaluate the performance of LRS using Oxford Nanopore sequencing to detect somatic variants across different tumour purities and sequencing depths by comparing to somatic variants from short-read sequencing. We generated experimental mixtures of cancer cell lines and matched normal cell lines to simulate 10 tumour purities (ranging from 0 to 100% tumour content). This resulted in 22 samples, which were sequenced using whole-genome LRS to a targeted depth of 60x, and somatic variants were identified. We down-sampled data to explore the optimal read depth for somatic variant detection. Our results show long-read variant calling tools achieve recall rates comparable to short-read gold standards. While tumour sequencing depth of 30x–60x is generally sufficient for detecting common variants, particularly structural variants, sequencing the matched normal sample at adequate depth is crucial for accuracy. Notably, we found variants unique to LRS that may represent real and previously undetected events.

**Conclusion:**

This study highlights key factors for optimising cancer genome sequencing with LRS and provides a comprehensive dataset of cell line mixtures for future research and tool development.

## Introduction

Cancer is a complex and multifaceted disease characterized by diverse genetic and epigenetic alterations, such as single-nucleotide variations (SNVs), structural variations (SVs), and changes in base methylation [1–3]. These genomic changes drive abnormal cellular proliferation and carcinogenesis [4], and represent targets for treatment [5] making the detection of mutations in tumour cells crucial for cancer research and precision therapy [6, 7]. While high-throughput short-read sequencing (SRS) has been widely used to identify somatic mutations across various cancer types, it often fails to detect variations in low-complexity, highly repetitive, or GC-biased regions due to limited mappability [8–10]. Long-read sequencing (LRS) technologies, such as those developed by Pacific Biosciences [11] and Oxford Nanopore Technologies (ONT) [12], offer extended read lengths that can span highly repetitive genomic regions effectively [13]. Initially used to enhance reference genome assembly and to resolve haplotypes [14, 15], LRS is increasingly applied in cancer genomics due to its ability to identify novel variations in previously unresolved regions of tumour genomes [16–20]. Recent studies using high-throughput platforms like the Nanopore PromethION have enabled rapid and comprehensive profiling of various advanced cancer cohorts [20] and central nervous system tumours [21, 22], demonstrating significant potential for clinical applications. However, the use of LRS in cancer has largely been restricted to a small number of studies or the analysis of cell lines, which underscores the importance of benchmarking these technologies to optimize their clinical and research use.

The evolution of LRS technologies has led to a proliferation of approaches for analysing the resulting data [23], including basecalling, alignment, and variant calling. LRS requires computational methods that are distinct from SRS methods due to its longer read length and unique error profiles [24, 25]. Benchmarking studies have evaluated various LRS tools to analyse cancer genome data, including aligners, somatic structural variation detection, and methylation detection methods [26–28]. However, many of these evaluations relied on *in silico* data or sequence data from cell lines [29, 30], meaning they did not capture complexities of tumour tissue samples such as tumour purity. Notably, some benchmarking efforts are published alongside the development of new tools, which can introduce bias as optimal parameters may not be used for each tool [31–34]. Therefore, independent benchmarking is valuable with the continuously emerging new tools and frequent version updates for existing tools [35].

Tumour purity and sequencing depth have a significant impact on the data quality and interpretation in tumour genomics studies [36–38]. Tumour purity refers to the proportion of cancer cells in a sample, and it can affect the signal-to-noise ratio, particularly for detecting somatic alterations (mutations specific to the cancer cells in a sample). Low tumour purity can mask somatic mutations by lowering the somatic variant allele frequency, potentially leading to false negatives [39]. Sequencing depth determines the sensitivity for identifying low-frequency variants as well as the ability to distinguish signals from germline cells; therefore, sequencing depth is particularly crucial in heterogeneous tumours [35] where low tumour purity and subclonal mutations are prevalent. Together, tumour purity and read depth influence the ability to detect somatic mutations, analyse clonal evolution, and understand tumour heterogeneity. Inadequate tumour purity or sequencing depth can result in the incorrect detection of clinically relevant alterations, which will negatively impact genomic discovery for cancer research and precision medicine [38, 40]. The implications of tumour purity and sequencing depth for LRS methods have not been explored.

In this study, we created cancer reference samples from two pairs of matched tumour-normal cell lines to measure the impact of tumour purity (0–100%) and sequence depth on somatic mutation detection. We sequenced the samples to an average read depth of >60x using Oxford Nanopore LRS. We compared the somatic variant calls from different experimental settings against our SRS gold standard to determine optimal sequencing strategies. The results establish guidelines for expected data quality based on differing tumour purity levels and sequencing depths, providing valuable insights for optimizing experimental design in tumour sequencing studies. Furthermore, the generated dataset serves as a valuable resource for the development and validation of bioinformatic tools in this rapidly evolving field.

## Results

### Study design and overview

**Figure 1:**
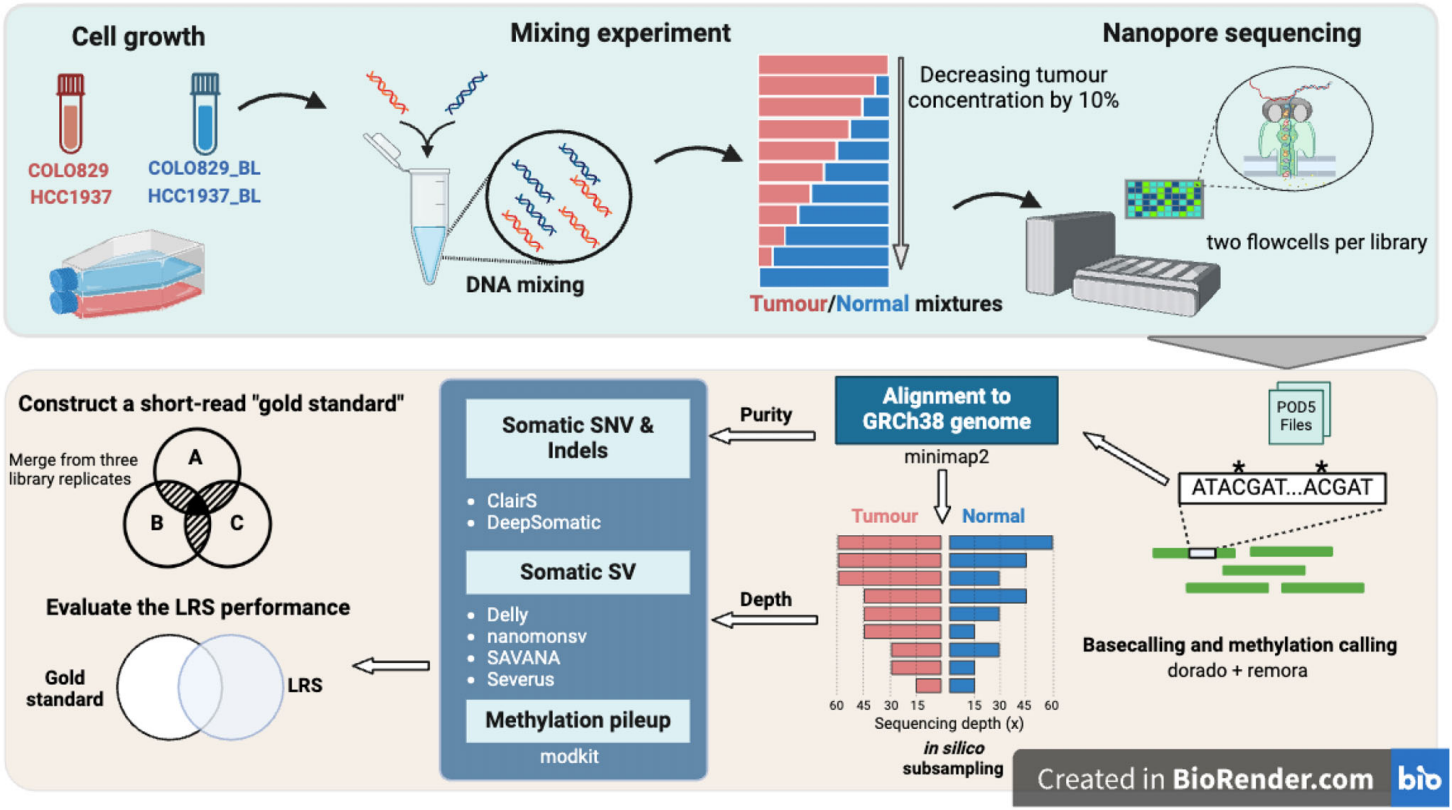
The overview of the experiment design. Two tumour-derived cancer cell lines, COLO829 and HCC1937, with their patient-matched non-tumour B lymphoblastoid (BL) cell line, were cultured and DNA extracted. The DNA was quantified, and tumour and non-tumour DNA samples were mixed to obtain tumour cell mixtures with decreasing tumour purity by 10% decrements. Libraries for each cell line mixture (*n* = 11 for each tumour cell line) were sequenced with the Nanopore PromethION sequencer with two flow cells per library. The raw data were basecalled with Dorado, and the 5mC methylation at CpG sites was called with a matched Remora model during basecalling. Sequence data were aligned to GRCh38 assembly and somatic SNVs, indels, and structural variations were identified for samples with different tumour purity levels. Sequencing depths were simulated by subsampling reads at 60x, 45x, 30x, and 15x for samples with 100, 80, 60 and 40% tumour purity levels. These combinations of sequencing depths (*n* = 9 per tumour cell line) were used for somatic SNV, indels, and SV detection. Short-read gold standard somatic mutations were constructed by overlapping high-confident sets from SRS of three replicate libraries for each cell line. Finally, the somatic variation results were compared with the short-read gold standard to evaluate the performance of LRS under different tumour purity and depths. The figure was created using BioRender (https://www.biorender.com).

We generated two tumour-normal paired cancer samples to simulate different tumour purities and sequence read depths to evaluate the ability of LRS to identify somatic mutations (Fig. 1). We cultured two cell lines representing distinct tumour types (melanoma, COLO829 and breast cancer, HCC1937) and a matched non-tumour cell line for each. DNA was extracted from each cell line and matched tumour- and non-tumour-derived DNA were serially mixed to simulate 10 tumour purity levels at 10% increments (0 to 100%). This resulted in 10 tumour mixtures and one matched blood lymphocyte (BL, as a germline/normal control) sample per cell line pair. A sequencing library was made for each sample and sequenced with the PromethION from ONT using two flow cells per library (R10.4.1). Basecalling was performed using Dorado (v0.5.1) with the super accuracy (SUP) model. The mean and median read quality scores were 21.8 and 23. The average yield per flow cell was 126 Gb, and the read length N50 was 10.4 kb (Supplementary Fig. S1A).

Two experiments were designed to assess the impact of common variables on somatic mutation calling in cancer samples: tumour purity and sequence read depth. To assess the impact of tumour purity, the 10 tumour cell mixtures and BL germline samples were sequenced to a mean depth of 70.61x for COLO829 (range 58.29x to 79.13x) and 61.57x for HCC1937 (range 53.42x to 71.14x) (Supplementary Fig. S1B), resulting in >95% of the genome being covered by at least 1 read (Supplementary Fig. S1C). The N50 (median aligned read length) for each library ranged from 9.4 to 10.4 kb (Supplementary Fig. S1D). We assessed impact of alignment using different human reference assemblies, CHM13-T2T and GRCh38. Slight improvements were observed using the CHM13-T2T reference with lower sequence divergence between reads and reference sequences and higher mapping rates (Supplementary Table S1). However, in our study,we used the GRCh38 assembly due to the comprehensive annotations and maximized compatibility with current tools and our short-read gold standard. To evaluate the performance of mutation detection under different sequencing read depths, we randomly subsampled sequence reads from four tumour mixtures (100, 80, 60, and 40%) per tumour type (COLO829 and HCC1937) and the matched BL sequence data to create tumour and germline BAM files with 60x, 45x, 30x, and 15x sequencing read depths. This resulted in nine combinations of sequencing depths.

In both experiments, we compared multiple approaches to call somatic single-nucleotide variants (SNVs), small insertions and deletions (indels), and structural variants (SVs). To assess the accuracy of the mutations detected with the LRS, we created a “gold standard” call from short-read data by sequencing DNA from the two tumour and matched non-tumour cell lines using three short-read replicate libraries. Sequencing resulted in a minimum read depth of 34x in BL and 67x in tumour samples, and we identified 40,422 SNVs, 416 indels, and 57 SVs in the “gold standard” short-read somatic calls for COLO829 and 48,982 SNVs, 3,236 indels, and 573 SVs for HCC1937 (Supplementary Fig. S2A–F).

### Tumour purity impacts somatic mutation detection

Patient tumour tissues have different tumour purities; therefore, we created 10 tumour mixtures for each cell line to explore how tumour purity impacts somatic mutation detection from LRS. Two approaches were used for somatic SNV and indel detection from long-read data. ClairS [32] and DeepSomatic [31]. The shifted distributions of variant mutation allele frequency (VAF) for SNVs (Supplementary Fig. S3A) and methylation frequency at CpG sites that are differentially methylated between the tumour and normal cell lines (Supplementary Fig. S3B) support the serial dilutions of tumour DNA by normal cell DNA.

**Figure 2:**
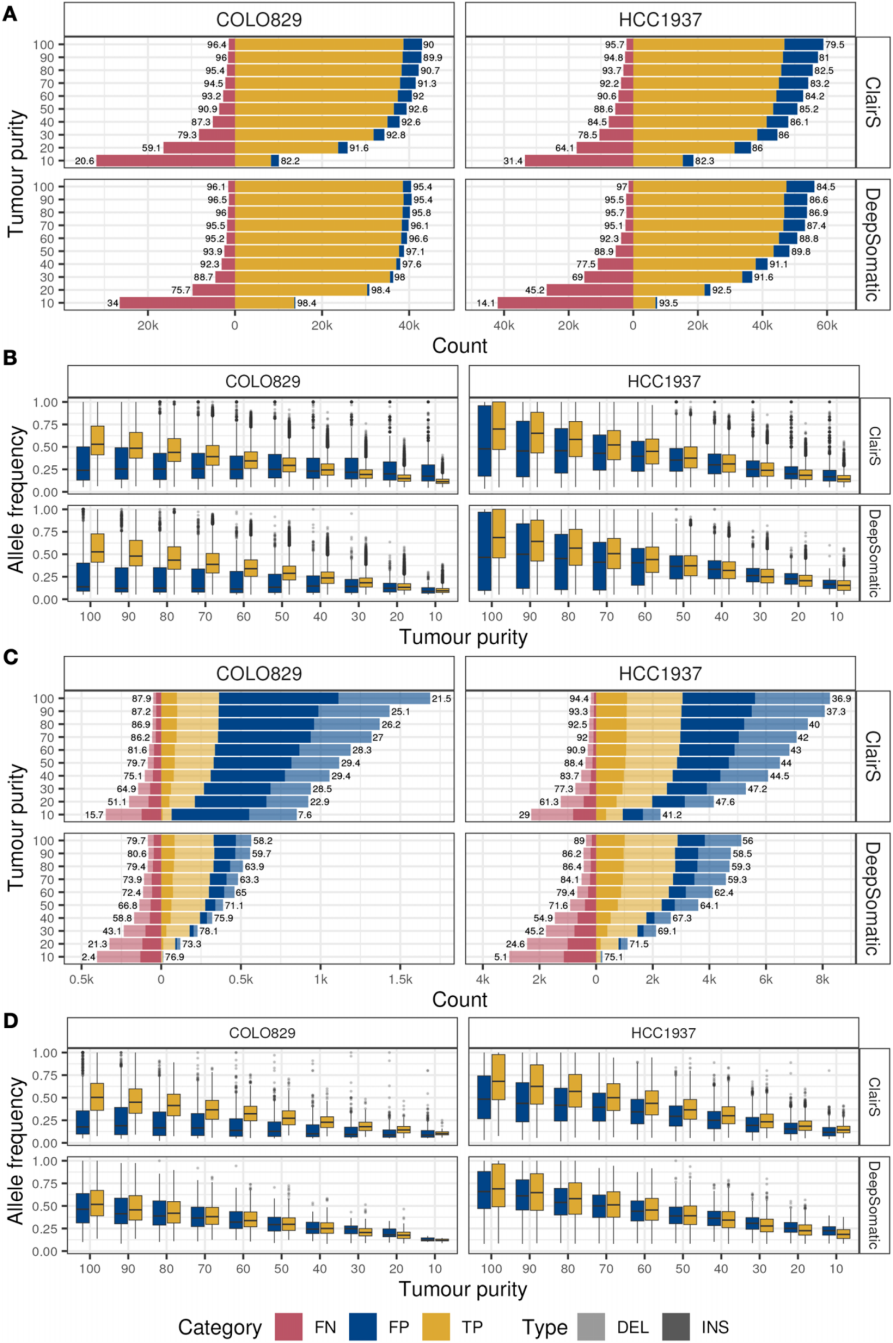
Tumour purity affects somatic SNV and INDEL calling. (A) Number of true positive (TP), false positive (FP), and false negative (FN) SNV calls in COLO829 and HCC1937 tumour cell mixtures using ClairS and DeepSomatic. The size of each call was present on the right side of 0 on the *x*-axis, and the missed call size on the left side. Precision and recall rates are labelled at the edges of the right and left sides of each row. (B) Boxplots comparing the distribution of variant allele frequency (VAF) for FP and TP in SNV calling with decreasing tumour purity. (C) The bar plot displays the number of TP, FP, and FN indel calls. The pale colour represents the deletions, and the solid colour shows the insertions. Precision and recall rates are labelled at the edges of the right and left sides of each row. (D) Boxplots comparing the distribution of VAF for FP and TP in indel calling with decreasing tumour purity.

Overall, the number of somatic mutations detected by ClairS and DeepSomatic decreased with decreasing tumour purity, resulting in a lower recall (Fig. 2A). Nevertheless, even at low tumour purity, the precision remained high (>79.5%). DeepSomatic had a higher precision than ClairS for COLO829 (ClairS range from 82.2 to 92.8%, DeepSomatic range 95.4 to 98.4%) and HCC1937 (ClairS range 79.5 to 86.1%, DeepSomatic range 84.5 to 93.5%) (Fig. 2A and Supplementary Fig. S4). In terms of recall, DeepSomatic had a higher mean recall at all tumour purity levels except the sample with 100% tumour purity compared to ClairS for COLO829 (ClairS 81.27%, DeepSomatic 86.39%); however, ClairS had a higher mean recall than DeepSomatic in HCC1937 in samples with lower tumour purity (<50%) (ClairS 81.43%, DeepSomatic 77.02%). To confirm these results, we performed a data simulation using the COLO829 data aligned to chromosome 22 with NanoSim [58] to introduce defined somatic SNVs to establish a ground-truth mutation set. This data confirmed that both ClairS and DeepSomatic show high (>99%) precision and recall at 100% tumour purity, with DeepSomatic achieving higher recall at lower purities than ClairS for this sample (Supplementary Table S3).

Within the sequenced mixtures (not simulated data), the majority of the SNVs were detected by both approaches, with 90% of SNVs being detected by ClairS and DeepSomatic for the COLO829 non-diluted sample and 87% for HCC1937 (Supplementary Table S2). The union of the two approaches achieved higher recall at the cost of introducing more FP events. The variant allele frequency of true positive (TP, present in “gold standard”) calls decreased with decreasing tumour purity for both COLO829 and HCC1937 (Fig. 2B) as expected. In contrast, the false positive (FP, absent from “gold standard”) calls for COLO829 do not show a decrease in AF, with FP having a greater range of AF in both COLO829 and HCC1937 at higher tumour purities (Fig. 2B). Interestingly, for ClairS, the read depth for FP calls was consistently lower than for TP calls (Supplementary Fig. S5A). This was not the case for DeepSomatic, suggesting that DeepSomatic FP variants might be TP variants missed by SRS meanwhile read depth could be used as a filter to remove real FP events in ClairS. The quality scores were lower for FP events compared to TP for both callers (Supplementary Fig. S5B). The different patterns observed in allele frequency, read depth, and quality score distributions between TP and FP events suggest that integrating these measures could serve as an efficient filter to reduce FP calls.

The average recall for indels (ClairS 71.62% for COLO829, 80.28% for HCC1937; DeepSomatic 57.85% for COLO829, 62.66% for HCC1937) (Fig. 2C) was lower than SNV (Fig. 2A). ClairS had a higher recall for indels at all tumour purity levels than DeepSomatic. Similar to SNV detection, the number of somatic indels detected by ClairS and DeepSomatic decreased with decreasing tumour purity, resulting in a lower recall (Fig. 2C). The average precision was significantly lower for indels (ClairS: 24.6% for COLO829, 42.39% for HCC1937; DeepSomatic: 68.55% for COLO829, 64.27% for HCC1937) than for SNVs (ClairS: 90.57% for COLO829, 83.61% for HCC1937; DeepSomatic: 96.87% for COLO829, 89.29% for HCC1937). The ClairS approach consistently had a lower precision in all tumour purities compared to DeepSomatic for both cell lines (Fig. 2C). There was no difference between the detection of insertion and deletion events (Fig. 2C).

Similar to the SNVs calls, the mutation allele frequency of true positive (TP, present in “gold standard”) indels calls decreased with decreasing tumour purity for both COLO829 and HCC1937 (Fig. 2D). The AF of TP indels was higher than the FP calls for ClairS but not DeepSomatic, suggesting that AF could be used as a filter to remove FP indel events in ClairS. The read depth for FP and TP calls was similar for indel events in ClairS and DeepSomatic (Supplementary Fig. S6A), suggesting that read depth is not useful for determining TP events. The quality scores were lower for FP events compared to TP for both callers (Supplementary Fig. S6B), suggesting that quality could be used as a filter to remove potential FP calls.

**Figure 3:**
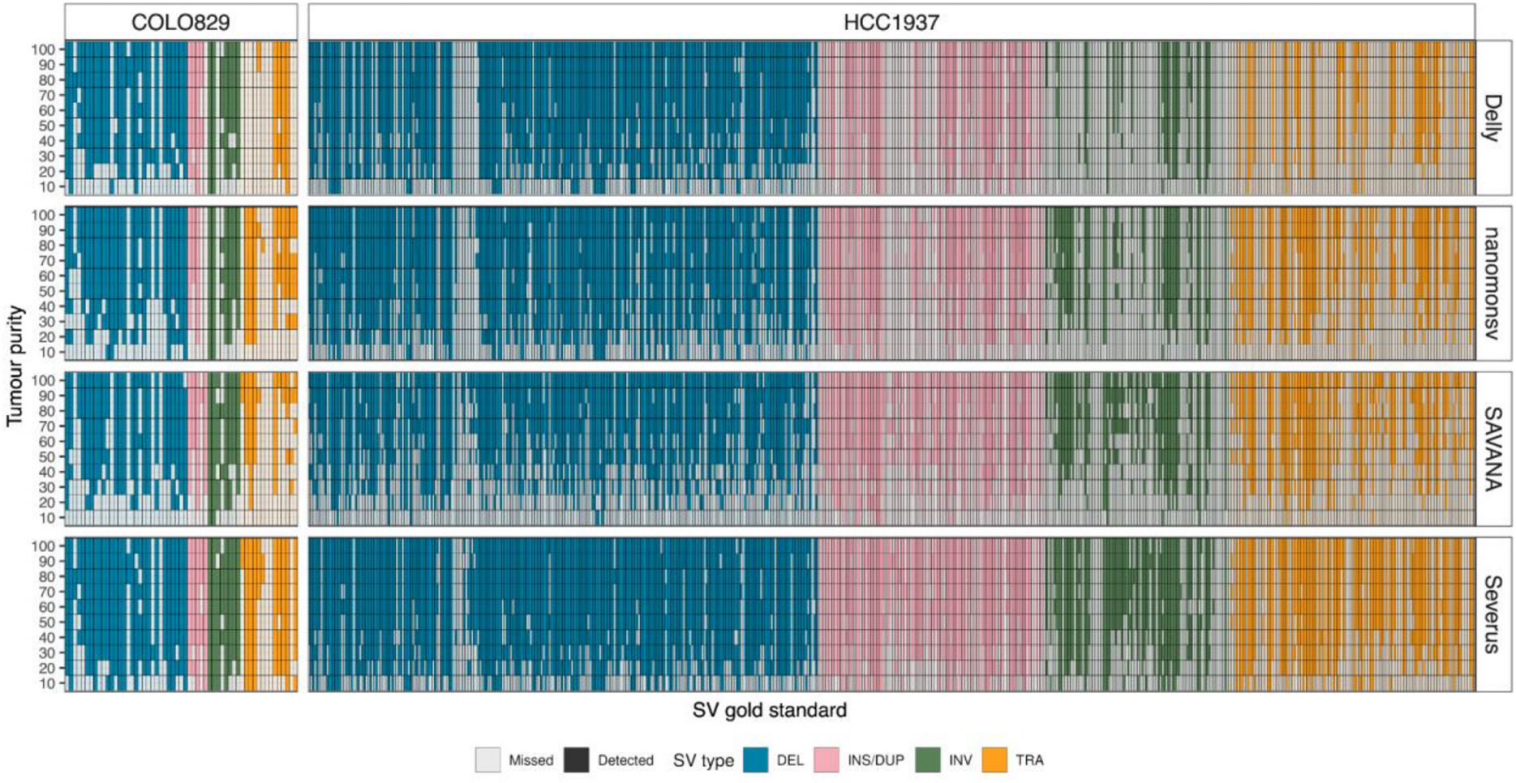
Tumour purity affects somatic SV calling. A heatmap of short-read “gold standard” SV events that are detected by long read using four SV callers in decreasing tumour purity levels for COLO829 and HCC1937 tumour cell lines. SVs of different SV types (DEL: deletion, INS/DUP: insertion/duplication, INV: inversion, TRA: translocation) are filled with different colours.

We compared the SV calls under different purities using four SV detection tools in two cell lines. Most SV calls in the short-read “gold standard” are identified with long-read SV callers; however, with 3 out of 57 in COLO829 and 87 out of 573 HCC1937 short-read “gold standard” SVs were not detected by any LR tool in all tumour purity levels (Fig. 3), suggesting these events may be missed by LRS or false positive calls in the short-read data. Samples with higher purity usually generate more SV calls (Supplementary Fig. S7), with a decreasing SV count as purity decreases, particularly in HCC1937, where there are more SV events (573, compared to 57 in COLO829). The Severus tool displays the best recall rates (36.84–84.21% in COLO829 and 26.18–78.53% in HCC1937) at all tumour purity levels for both cell lines. Even at 20% tumour purity, the recall for Severus is 63.16% for COLO829 and 50.79% for HCC1937. In contrast, the recall for other tools at 20% tumour purity for COLO829 and HCC1937, respectively, was 42.11 and 36.47% for Delly, 40.35 and 35.25% for nanomonsv, and 28.07 and 20.59% for SAVANA. The recall rate for SAVANA was comparable to nanomonsv in higher purity samples (>60%); however, SAVANA did not perform as well in lower purity samples (Supplementary Fig. S7).

### Sequencing depth affects somatic mutation calls

We sub-sampled tumour and germline samples to simulate different read depths (60x, 45x, 30x, and 15x) and then compared the somatic mutation detection under four purities (100, 80, 60, and 40%). This resulted in nine scenarios of tumour-germline sequencing depths under four tumour purities (Fig. 1).

**Figure 4:**
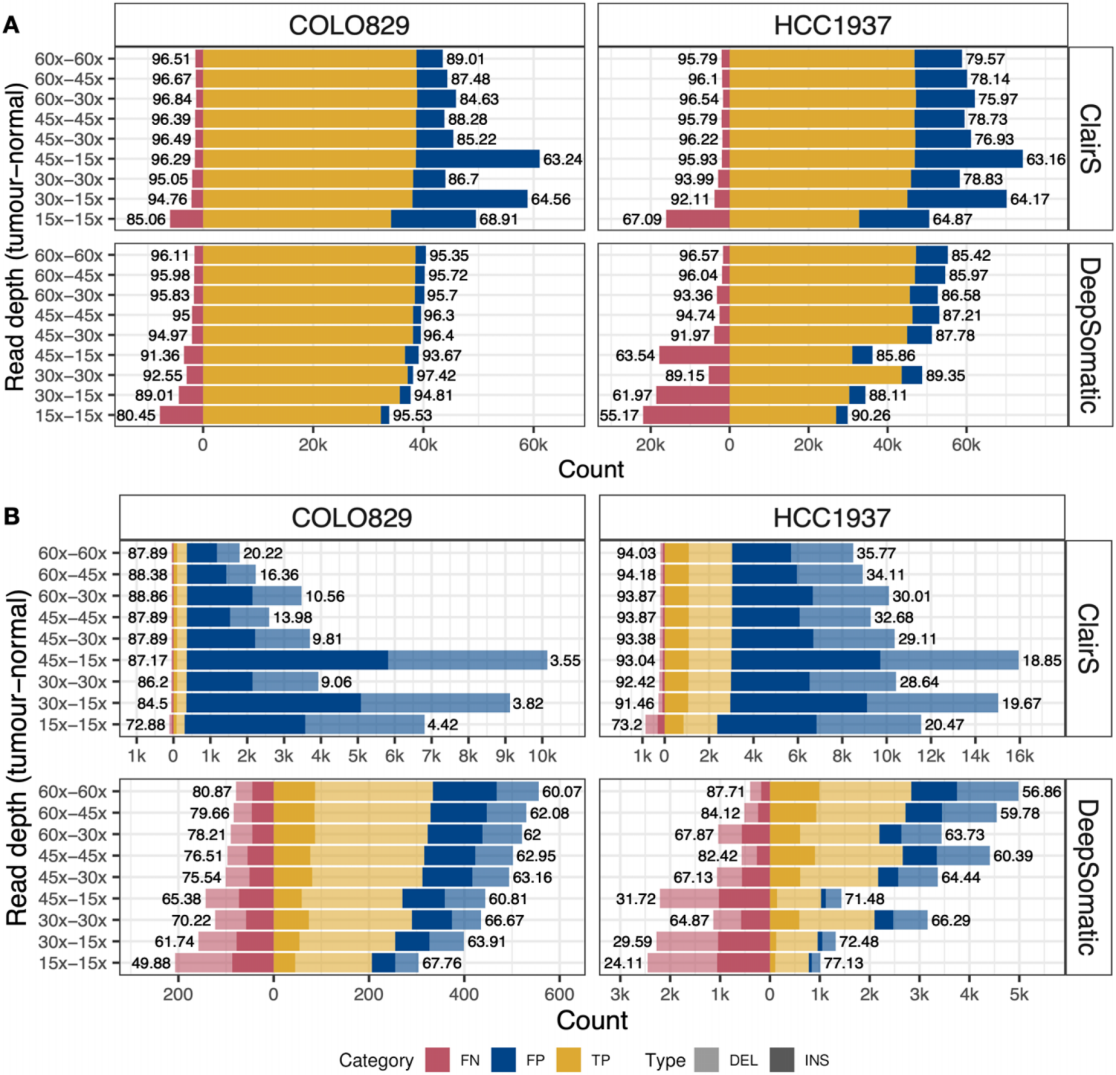
Sequencing depth affects somatic SNV and indel calling. Number of true positive, false positive, and false negative somatic variant calls under nine depth combinations (*y*-axis) of tumour and normal samples in COLO829 and HCC1937 using ClairS and DeepSomatic in samples with 100% tumour purity. (A) Somatic SNV calling performance in recall and precision of ClairS and DeepSomatic. (B) Somatic indel calling performance in recall and precision of ClairS and DeepSomatic, deletions and insertions were filled with stacked bars in different alpha.

Lower read depth in the germline sample is expected to impact somatic SNV detection. For ClairS, this resulted in an increase in recall and a decrease in precision, whereas for DeepSomatic, it resulted in a lower recall and a higher precision. When the germline samples were sequenced at 15x sequencing depth, the performance of these approaches was severely impacted, a finding that was confirmed in simulated data (Supplementary Table 3). Within the sequenced mixtures (not simulated data), using the samples with 100% purity, the precision dropped below 65% in both cell lines for ClairS, and the recall also dropped below 65% in HCC1937 for DeepSomatic (Fig. 4A). Furthermore, we observed the average allele frequency of FP SNVs when the normal sample at 15x depth higher than other read depth combinations (Supplementary Fig. S8). Similarly, in indel calling, lower sequencing depth in normal samples leads to decreased precision but increased recall for ClairS except when normal samples had only 15x read depth, whereas DeepSomatic results in reduced recall but improved precision (Fig. 4B). Interestingly, ClairS demonstrates robustness to the impact in the HCC1937 cell line, while DeepSomatic shows greater robustness in COLO829. Similar results were observed for samples with lower tumour purity (Supplementary Fig. S9).

Sequencing depth in both tumour and normal samples had a smaller impact on SV calling than SNV and indel calling, particularly for nanomonsv and Severus (Supplementary Fig. S10). Nanomonsv consistently demonstrated the highest precision in four tools across tumour purities, underscoring the importance of incorporating a reference panel to achieve accurate variant detection. Meanwhile, SAVANA is more sensitive not only to tumour purity but also to sequencing depth. In terms of recall, Delly outperformed SAVANA in recall when germline samples were sequenced at 15x, although its recall remained lower than that of nanomonsv and Severus. Severus exhibited the highest recall among the four tools across all depth combinations and detected more LR-specific (FP) SVs than nanomonsv, particularly in scenarios with lower depth in the normal sample. While the LR-specific SVs may represent novel SVs, they could also be read false positives.

### False positives detected by LRS might be true

**Figure 5:**
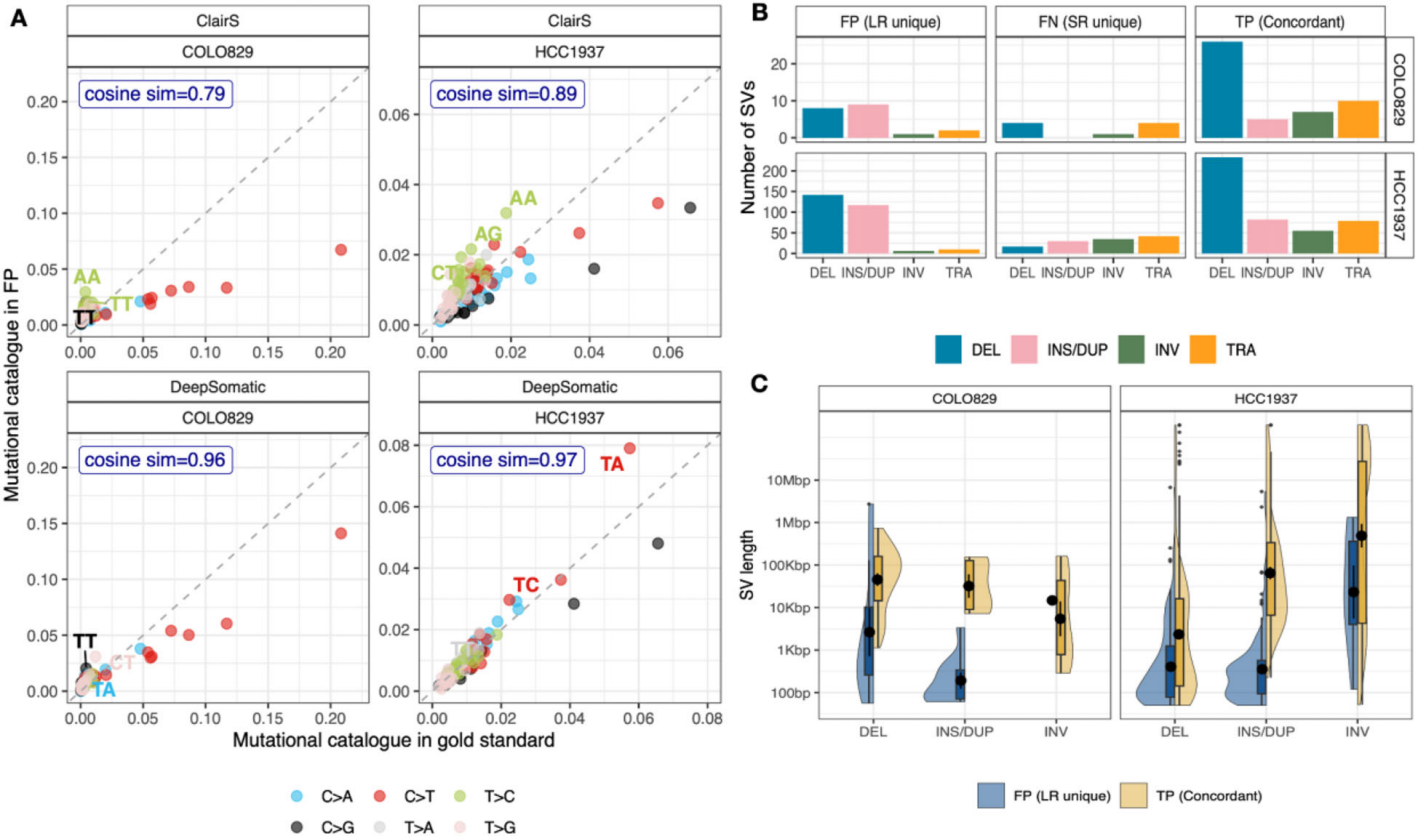
Potential novel events detected by LRS. (A) The comparison of the 96 mutational catalogues between the short-read gold standard and FP from LRS. (B) The number of SVs in four types (DEL: deletion, INS/DUP: insertion/duplication, INV: inversion, TRA: translocation) from FP, FN, and TP. (C) The SV length distribution of FP and TP events in DEL, INS/DUP, and INV types, the black dot in each bar indicates the mean value.

Despite the high recall rate in variant detection using LRS compared with SRS, we found that many LR-specific false positive calls might be real. The proportions of single substitution signatures (SBS) catalogues extracted in the false positives had high cosine similarity to our short-read gold standard with 0.79 and 0.89 for ClairS, 0.96 and 0.97 for DeepSomatic in COLO829 and HCC1937 (Fig. 5A). Notably, ClairS detected more T to C (A to G) type mutations with a context AA existing in FP-positive calls for both cell lines. We also found that false positive SNVs from ClairS tend to cluster along the centromere and telomere regions (Supplementary Figs S11 and S12) along chromosomes for both cell lines compared with DeepSomatic (Supplementary Figs S13 and S14). This suggests that DeepSomatic has more stringent variant calling at difficult genome regions. The genome stratification of false positive SNVs shows that ClairS calls more FPs in tandem repeats and low mappability regions, while DeepSomatic calls more FPs in areas of high or low GC%. Meanwhile, the genome stratification of false positive indels displays a similar pattern between the two tools with a higher proportion of FPs overlapped with tandem repeats and genomic regions with GC percentages <30% or > 55% (Supplementary Fig. S15).

The high-confidence LR SV calls presented in at least two out of four tools also contain many LR-specific SVs. While these SVs may appear as false positives, we expected some of these SVs to be real due to the technical advantage of long-read over short-read. In support of this, we found that a substantial fraction of the LR-specific SVs are located at genomic regions of extreme GC content (GC% <30 or >55) or within low mappability for short reads (Supplementary Fig. S16). Furthermore, these events are mostly deletions, insertions, and duplications with shorter lengths (<10 kb) (Fig. 5B, C), which means they can be sequenced by a single long read with high confidence. Meanwhile, SRS might miss these smaller SVs (<10 kb) due to the restriction of the length of reads and the uncertainty of the pair-end library sizes.

### Germline leakage is more related to the sequencing depth but not tumour purity

Germline leakage refers to germline mutations being misclassified as somatic calls in the variant detection pipeline [41]. We estimate the germline leakage rate by quantifying somatic calls with a maximum population frequency above 0.1 in gnomAD (version 4.1) [42]. While this threshold may not capture all germline variants, we used these values to compare the germline leakage across tumour purity and sequencing depth. We found that the germline leakage rates remain low across tumour purity and start to increase with tumour purity less than 30% for ClairS while decreasing for DeepSomatic (Supplementary Fig. 17A). Notably, we found that the leakage spiked when the sequencing depth in the germline was 15x, suggesting enough depth in germline samples is crucial for avoiding germline variation contamination in somatic mutation detection (Supplementary Fig. 17B).

### Benchmarking SV using publicly available gold standard

Using short-read data to build the SV gold standard is a challenge due to technical limitations of SRS to detect the full set of SVs in the genome. Previously, a COLO829 SV truth set SV, including 68 SVs, was thoroughly characterized with multiple sequencing platforms, including short-read and long-read technologies [43]. We benchmarked our long-read SVs to this truth set to evaluate how many SVs that were missed by our SR gold standard can be detected by LRS. The recall rates are slightly lower than the results when benchmarking with our SR gold standard (Supplementary Fig. S18). This suggests our “gold standard” captured most SVs; however, differences exist because our “gold standard” was constructed using single-platform sequencing data and also genetic changes may accumulate in cell line passaging causing cells from different organizations to increasingly differ across time.

## Discussion

In this study, we sequenced a series of *in vitro* cell line mixtures with different tumour purity levels from two cancer types and compared the performance of long-read somatic variant detection tools, including SNVs, indels, and SVs (≥50 bp). Additionally, our *in silico* sequencing depth down-sampling allowed us to evaluate the impact of sequencing read depths of tumour and normal samples on variant detection. Our results provide a practical guide for cancer genomic sequencing of clinical samples, and the dataset of sequenced mixtures could be valuable for benchmarking the robustness of variant calling tools under different tumour purity settings.

Previous studies have benchmarked LRS for variant calling in a range of genomic contexts, but many focused on germline-specific variants or prioritized specific genomic features such as SVs [27], CpG methylation [44], and mRNA [45], some with the impact of tumour purity addressed by the *in silico* mixing dataset [29]. However, there remains a critical need to benchmark LRS for variant calling in the context of tumour genomics. Unlike germline variants, somatic mutations in tumours exhibit substantial heterogeneity, both within and across cancer types, posing unique challenges for accurate detection [46]. Through experimental mixing DNA of tumour and normal cell lines and *in silico* down-sampling data, we showed that LRS tools can detect >50% of the variants of all kinds from a short-read “gold standard” at tumour purities as low as 20% and for 30x–60x sequencing depth in tumour. Importantly, our data show that having enough depth in germline sample (>15x) is critical for recall and precision of somatic mutations. These findings can guide parameters for clinical sequencing protocols.

As expected, tumour purity significantly affects somatic mutation detection, with lower purity reducing sensitivity across SNVs, indels, and SVs. This highlights the challenge of identifying rare somatic alleles in samples with significant stromal cell content. However, even at low tumour purity (e.g., 20%), LRS, especially with Severus for SVs, maintains a reasonable recall rate, demonstrating its utility in low-cellularity samples. Although Nanopore sequencing has lower base qualities (Q20–Q30) than the current SRS (Q30–Q40), deep-learning-based SNV callers like ClairS and DeepSomatic effectively mitigate sequencing errors. ClairS combines pileup and full-alignment models using Bi-GRU and ResNet architectures to classify variants as germline, somatic, or artefacts [32]. DeepSomatic, using a CNN-based model, processes alignment pileups as images [47]. These architectural differences likely drive performance differences. DeepSomatic’s higher precision for SNVs and indels suggests a more conservative, error-resistant approach, possibly due to its training data and algorithm design. While ClairS achieves comparable SNV recall and better indel recall, it has lower precision, suggesting it requires stringent downstream filtering of variants based on biological context. Notably, ClairS detects variants in centromeric and telomeric regions, indicating its potential to identify mutations in genomic regions traditionally inaccessible to short reads. These results suggest that despite lower base qualities, LRS is able to accurately call somatic mutations even in samples with low tumour content.

Among the four long-read SV detection tools evaluated, Severus consistently outperformed Delly, nanomonsv, and SAVANA across varying tumour purities and sequencing depths. Severus maintained high recall even at low tumour purity and sequencing depth (15×), suggesting its ability to detect tumour-specific signals with minimal interference from germline contamination. SAVANA’s performance declined at lower tumour purities and showed high sensitivity to reduced sequencing depth, potentially limiting its applicability in clinical settings. However, its recent integration of tumour purity, ploidy, and copy number alteration inferences may address these problems [34]. Nanomonsv performed similarly to SAVANA but remained more robust at lower tumour purities and sequencing depths. Its use of an internal human germline SV reference panel makes it more conservative in SV detection, which aligns with previous findings [27, 48].

LRS inherently offers advantages for SV detection over short-read approaches. Given that SNV and small indel detection show comparable performance [49] between long- and short-read technologies [50], a key question is whether LRS enhances SVs discovery by detecting variants missed by SRS. While previous studies suggested that most large SVs in tumour genomes are detectable by short reads, our findings indicate that long reads uniquely capture numerous short-to-intermediate SVs (<10 kb), which were underrepresented in earlier analyses [51]. This further underscores the importance of LRS in tumour genomics, particularly for identifying novel SV markers beyond the resolution of short-read methods.

To enable future cancer genomics studies and translate LRS into the clinic, we need to know what read depth is required to reliably detect somatic mutations. Our sequencing depth down-sampling experiment suggests that balancing coverage between tumour and normal samples can improve somatic variant detection potentially by minimizing germline contamination. Furthermore, sequencing depths of 15x or less for the germline sample significantly harm somatic variant calling and should be avoided. In our study, we sequenced both tumour and normal samples using two flow cells aim for a read depth of 60x. While we obtain data of ∼30x genomic sequencing coverage per flow cell, a study has shown that a single Nanopore PromethION flow cell can generate >33× human genome coverage with ultra-long reads (N50 >35 kb) with optimized DNA processing and library preparation [52]. Assuming that sequencing with a shorter (10 kb) read length N50 produces higher yields per flow cell [49], in the future we can reduce sequencing costs by using only one flowcell each for tumour and normal.

Although long reads allow improved mapping in genomic regions of low complexity, the alignment accuracy remains imperfect [53]. Some suggest the reason to be low read accuracy; however, the main factor could be the genome assembly [54]. We used the GRCh38 assembly in this study as it provides more comprehensive clinical annotations for the downstream analyses; however, GRCh38 remains incomplete with unresolved gaps and repetitive sequences of unknown size. Recent studies suggest that using the CHM13-T2T [14] assembly results in better genetic variant detection from more precision alignment, especially in highly repetitive regions such as centromeres and telomeres [55, 56]. Future studies exploring the impact of reference assemblies on variant calling performance may provide valuable insights into cancer genome sequencing.

The “gold standard” used in this study was constructed with the SRS with all the limitations that imply. Previously, researchers have utilized the synthetic mixtures of two fully homozygous human cell lines to mock somatic mutations [57], and apply read simulators such as NanoSim [58] or pbsim2 [59] to upsample data for validation of variant detection. While this is a good strategy to explore variant calling performance under extreme scenarios, differences in variation calling performance were found when using the real tumour cell line [33], which underscores the heterogeneous nature of cancer samples [60]. A major output of our study is the creation of reference data (with a range of tumour purities) that can be used by research community. To this end, we deliberately selected two cancer types, one with a high SNV burden, the other with a high SV burden. Establishing references for other key features of cancer genomes, such as subclone mutations, tandem repeats, and complex SVs, will largely facilitate the development of better computational tools [61, 62].

This study explored the optimal experimental design for cancer genome sequencing using LRS through creation of tumour purity mixtures. Many LRS analysis tools are still under active development, and while we are far from a definitive “best practice” for cancer variant calling using LRS, we have investigated key aspects key and highlighted directions for improvement. Importantly, we provide a publicly available dataset of LRS cancer cell line mixtures, offering a valuable resource for future tool development and benchmarking in this fast-evolving field.

## Materials and methods

### Ethical statement

This work involves the use of human cell lines, which were approved by the QIMR Berghofer Human Research Ethical Committee (P3577 and P3527).

### Cell culture, DNA extraction, and mixing

COLO829 (RRID:CVCL_1137), COLO829_BL (RRID:CVCL_1999), HCC1937 (RRID: CVCL_0290), and HCC1937_BL (RRID:CVCL_3281) were maintained in RPMI +10% FBS. All cell lines were tested for mycoplasma and STR profiling. Cells were harvested between passages 8 and 10. Cells were dissociated using 0.25% trypsin at 37°C for 5 min and spun at 1,200 rpm for 5 min. Cells were washed once with PBS and kept at –80°C until DNA extraction. DNA was extracted using AllPrep DNA/RNA Mini Kit (802024, Qiagen) according to the manufacturer’s protocols and eluted in TE buffer. To prepare samples with various tumour purities, both tumour and normal DNA were diluted to 50 ng/µl using the TE buffer. The tumour and its corresponding normal DNA were mixed in specific ratios to create 10 samples that represent tumour purities ranging from 0 to 100% with increments of 10%.

### Library preparation and Oxford nanopore sequencing with PromethION

DNA quantity and quality were assessed using an Implen NanoPhotometer, then run on a TapeStation (Agilent) to evaluate fragment size. High molecular weight DNA was sheared to 10–12 kb using Covaris g-tubes. Approximately 1 µg of sheared genomic DNA was used to make libraries using the ONT ligation sequencing kit (SQK-LSK114), according to the manufacturer’s instructions, except that ligation was extended to 30 min. Each library was loaded at approximately 10 fmol on two separate R10.4.1 PromethION flow cells and sequenced for 72 h on a PromethION P24 sequencer within QIMR Berghofer Scientific Services.

### Basecalling, methylation calling, and alignment

The raw POD5 files from the sequencer were basecalled using Dorado (v0.5.1) [63] with the super accuracy (SUP) model for the R10 flow cell (dna_r10.4.1_e8.2_400bps_sup@v4.3.0). The 5mC and 5hmC methylation were also detected using Dorado with the matched Remora model (dna_r10.4.1_e8.2_400bps_sup@v4.3.0_5mCG_5hmCG@v1). SAMtools (v1.17) [64] was used to convert the unaligned basecalled BAM file from basecalling to FASTQ format and filter out reads with average quality (QS) <10 before alignment, and minimap2 (v2.26) [65] was used to align reads to the GRCh38 genome assembly (-y –MD -ax map-ont). The aligned BAM files were sorted and indexed using SAMtools. The percentage of 5mC methylated reads at CpG sites was calculated using modkit (v2.4.0) pileup based on the MM and ML tags of reads from both strands in the BAM files (–combine-strands –cpg).

### Read depth simulation

To evaluate the performance of variant calling under sequencing strategies, we used Sambamba (v1.0.1) [66] to downsample data from the tumour and the matched B lymphoblast BAM files with 60x, 45x, 30x, and 15x read depths in samples with 100, 80, 60, and 40% tumour purities. Reads were randomly sampled from the aligned BAM files at the proportion based on the expected and original depth. If a BAM file did not reach a 60x read depth, we used the original BAM file (53.42x for the library of HCC1937 cell line at 40% tumour purity). In total, we generated nine combinations of sequencing depths with either higher read depths in tumour samples (60x-45x, 60x-30x, 45x-30x, 45x-15x, 30x-15x) or equal read depths in both tumour and normal (60x-60x, 45x-45x, 30x-30x, 15x-15x). The depth of subsampled BAM files was confirmed with mosdepth (v0.2.9) [67] to check the potential effect of varied read lengths.

### Somatic SNV, indel, and SV detection

We used ClairS (v0.1.7) and DeepSomatic (v1.6.0) for SNV and short indel detection in tumour cells. These tools were selected as they are both actively updated and designed to call somatic SNVs and indels using a pair of tumour and normal BAM files to predict tumour-specific variants using pre-trained deep-learning models. For ClairS, we used the ont_r10_dorado_sup_5khz model for somatic SNV detection and the r1041_e82_400bps_sup_v430 model from rerio for Clair3. For DeepSomatic SNV calling, we used default parameters with an internal ONT model provided by Google Health. The final results in VCF were filtered for somatic SNVs with GT=”1/1” and FILTER=”PASS” using BCFtools (v1.19) [68] as described in the DeepSomatic document.

We used four tools (Delly (v1.2.6) [69], nanomonsv (v0.7.1) [70], SAVANA (v1.0.5) [34], and Severus (v1.0) [33]) to call SV using long reads. Each tool uses a pair of tumour and normal BAM files and runs with default parameters with SV length less than 50 bp were excluded. In addition, nanomonsv included an extra reference panel of 30 human genomes to filter somatic SVs. As part of the nanomonsv pipeline, SV events that overlapped with simple repeats were filtered and the SV type was annotated using the sv_type.py script of the package. Phased and haplotagged BAM files were provided to Severus, and variable number tandem repeat regions were used in the analysis. WhatsHap (v1.4) [71] was used to phase genomics variants in germline VCF files and add tags to each read in BAM files with known haplotypes.

### Short-read sequencing for cancer cell lines

DNA was extracted for COLO829 and HCC1937 cells and matched B lymphoblastoid cells using the Qiagen AllPrep DNA mini kit according to the manufacturer’s protocol (Qiagen, Germany). Whole genome analysis was performed on DNA from tumour and BL cells using DNA PCR-free prep and sequenced on a NovaSeq (Illumina).

### The short-read gold standard for SNVs and indels

We used the concordant high-confident somatic SNV calls based on three high-depth short-reads sequencing of COLO829 and HCC1937 as our “gold standard.” The cell lines were cultured from the same purchase within a few passages and thus can avoid extensive sub-clonal somatic mutations. The short-read data of three biological replicates were analysed with our in-house standard Cromwell pipeline to detect somatic SNVs with GATK (v4.0.4.0) [72] and qSNP (v2.1.4) [73] and somatic small indels with GATK. For merging SNVs, we first split multi-base variants into consecutive SNVs and also split multiallelic sites into multiple entries using the BCFtools norm module. Next, the intersection of high-confidence SNVs for three libraries was calculated using the BCFtools isec command. Finally, the three VCF files were merged and annotated with the intersection results using BCFtools merge. SNVs that are detected in at least two libraries were used as a short-read “gold standard.” The indel gold standard was generated similarly to SNV but without atomization.

### The short-read gold standard for SVs

For each library of COLO829 and HCC1937, we used Delly, GRIDSS (v2.13.2) [74], and LUMPY (v0.3.1) [75] to call SV from short-read data. The somatic SVs were extracted from each tool and merged within each library. The SVs that were detected by at least two tools were further merged between libraries for each cell line. Finally, SVs that were shared by at least two libraries were used as the SV gold standard. The merging of SVs is not as straightforward as SNVs. The output from each tool was first converted into a simple VCF format with five SV types (INS, INV, DEL, DUP, and TRA), and Jasmine [76] was used to merge multiple VCF files and to determine the overlapping.

### EPIC array data analysis

The intensity data (IDAT) files of each cell line were loaded and analysed with the minfi (v1.48.0) [77] R package. Specifically, the raw signals were transformed and normalized as beta values at each probe CpG position, and the probes that contained an SNP at the CpG interrogations or the single-nucleotide extension were excluded. To compare long-read methylation with EPIC array data, we performed liftover for EPIC array results from hg19 to hg38 using R packages GenomicRanges (v1.54.1) [78] and rtracklayer (v1.62.0) [79] with the chain file from the UCSC Genome Browser [80]. The methylation data were also used to confirm the tumour purity levels within the serial diluted samples. Specifically, differentially methylated CpG sites between the tumour and blood lymphocyte cell lines were determined as probes with beta values ≥0.7 in tumour and ≤0.3 in BL, or ≤0.3 in tumour and ≥0.7 in BL. The distribution of these CpG sites was then assessed in the samples with different tumour purities for COLO829 and HCC1937.

### Benchmarking

We benchmarked the somatic SNVs and indels calling using the Python script compare.py of ClairS. We also used hap.py for comparison, which generated similar results. All SNVs and indels with PASS filter were used as the target to calculate the recall, precision, and F1 values with the corresponding gold standard for each cell line and variant type as the truth set. For SV benchmarking, tools had different formats to represent SV events. We converted the breakpoint pairs from different tools into the simple type VCF format and merged them with the short-read gold standard using Jasmine with default parameters, and only SVs with a length greater than 50 bp were considered. The public COLO829 SV truth set (Version v4) was downloaded from Zenodo (10.5281/zenodo.7515830) and compared with the results for a 100% tumour purity sample. The recall and the precision rates were calculated as


\begin{eqnarray*}
\mathrm{ Recall} = \frac{\mathrm{ overlap\ calls\ between\ LRS\ and\ SRS}}{\mathrm{total\ calls\ from\ SRS}},
\end{eqnarray*}

\begin{eqnarray*}
{\mathrm{ Precision}} = \frac{{{\mathrm{ overlap}}\ {\mathrm{ calls}}\ {\mathrm{ between}}\ \mathrm{ LRS}\ \mathrm{ and}\ \mathrm{ SRS}}}{{{\mathrm{ total}}\ {\mathrm{ calls}}\ {\mathrm{ from}}\ \mathrm{ LRS}}},
\end{eqnarray*}

\begin{eqnarray*}
\mathrm{ FN }= {\mathrm{ unique}}\ {\mathrm{ calls}\mathrm{ }}\ \mathrm{ in}\ \mathrm{ SRS},
\end{eqnarray*}

\begin{eqnarray*}
\mathrm{ FP }= {\mathrm{ unique}}\ {\mathrm{ calls}}\ \mathrm{ in}\ \mathrm{ LRS}.
\end{eqnarray*}

### Genome regions and stratifications

To investigate the genomic distribution of variants identified as TP, FP, and FN, genome stratifications for GRCh38, including low complexity, segmental duplications, low mappability, and GC content outliers, were downloaded from the genome-in-a-bottle consortium (v3.4) [8]. The overlapping status of each variant in regions of different categories was determined in R with the GenomicRanges and VariantAnnotation (v1.48) [81] packages. The genome-wide distribution of TP, FP, and FN SNVs was visualized using rainfall plots, which were aligned with chromosome ideograms annotated with cytogenetic banding patterns (gieStain) obtained from the UCSC Genome Browser [82].

### Mutational signatures analysis

All SNV somatic mutations were converted into a 96-channel mutational profile (SBS-96 vector) and were subsequently decomposed into contributions of COSMIC reference catalogue (v2.0, GRCh38) using the R package deconstructSigs (v1.8.0) [83]. To evaluate whether LR-specific variants, classified as false positives, might represent genuine mutational events, we compared their mutational spectra with the short-read gold standard set. Specifically, a cosine similarity was computed between the two profiles.

### Germline leakage estimate

To assess the extent of germline contamination in somatic variant calling, we quantified the proportion of SNVs commonly observed in the general population. Population allele frequencies were retrieved using BCFtools, which was used to query each variant against the gnomAD VCF files (version 4.1) [42]. SNVs with a population allele frequency (AF_grpmax) greater than 0.1 were classified as germline variants.

### Visualization and IGV check

Data wrangling was implemented in R (v4.3.1) with tidyverse (v1.2.1) [84] and visualized using ggplot2 (v3.4.4) [85] and ggh4x (v0.2.8) [86]. The long-read- and short-read-specific SV events were extracted as BEDPE files, and the IGV view of regions was generated using igv-reports (v1.12.0).

### An *in silico* simulation with simulated COLO829 Nanopore sequencing data

Chromosome 22 was extracted from GRCh38 genome and integrated with gold standard germline SNVs and somatic SNVs of COLO829 and COLO829_BL using SAMtools and BCFtools. Nanopore reads were simulated using NanoSim (v3.2.3) trained on a subset of empirical data of COLO829_BL to approximate realistic read length and error characteristics. Matched tumour-normal datasets were generated based on the synthetic tumour and germline genomes at tumour purity 10, 20, 40, 60, 80, and 100% and sequencing depths of 15x, 30x, 45x, and 60x, respectively. These datasets were processed using the same alignment and variant-calling workflow as the empirical mixtures, and performance was evaluated against the known truth set.

## Availability of source code and requirements

Project name: nanopore_celllines_benchmark.

Project homepage: https://github.com/bakeronit/nanopore_celllines_benchmark.

Operating system: Linux (Rocky Linux 9.7).

Programming language: Python, R, Bash.

Other requirements: Snakemake≥8.2.0, tidyverse≥2.0.0.

License: BSD-3-Clause license.

## Additional files

**Supplementary Figure S1**. The quality of sequence data and alignment for tumour mixtures and matched normal samples. (A) Dot plot showing the total data yield (gb, gigabases) and read length N50 kb (kilobases) for each flow cell. Samples are ordered by sample type, then gb of sequence. (B) Total sequencing depth for each sample, with the *x*-axis showing the sample tumour purity and the *y*-axis indicating the sequence read depth. (C) The proportion of genome assembly covered by at least one read in each sample. (D) Alignment length N50 for each sample. Samples are coloured by type (blue: B lymphoblast non-tumour and red: tumour mixture).

**Supplementary Figure S2**. Creation of short-read “gold standard” somatic mutation calls for the COLO829 and HCC1937 cell lines. The COLO829 and HCC1937 tumour-derived cell lines and their matched non-tumour cell lines were sequenced in triplicate in short reads. For COLO829, the UpSet plots show the number of somatic (A) single-nucleotide variants (SNVs), (B) insertion and deletion (indels), and (C) structural variant (SV) events detected uniquely in each replicate and shared by three or two replicates. For HCC1937, UpSet plots show the number of somatic (D) SNVs, (E) indels, and (F) SV events detected uniquely in each replicate and shared by three or two biological replicates. Blue bars represent events shared by at least two replicates, which were used to define the gold standard.

**Supplementary Figure S3**. The distribution of variant allele frequency (AF) and methylation in samples with different tumour purities. (A) The distribution of variant allele frequency of SNVs at the genomic regions with a copy number equal to two in COLO829 and HCC1937. The lines are coloured by tumour purity from 100 to 10%. (B) The methylation frequency distribution in CpG sites (*x*-axis) in tumour samples with different tumour purities (*y*-axis) for COLO829 (left) and HCC1937 (right). Only the CpG sites with differential methylation frequency between the 100% tumour and blood lymphocyte cell lines are included in the plot. Plots are coloured by the methylation status in the 100% tumour sample with a high (red) or low (blue) methylation level.

**Supplementary Figure S4**. Precision-recall curves (AUPRC) for variant detection across different tumour purities. The precision (*y*-axis) and recall (*x*-axis) of long read calls against the short read “gold standard” are shown for SNVs (A), indels (B), and SVs (B). Points are coloured by tumour purity. In panels (A) and (B), points in circles shape represent ClairS results, and triangles represent DeepSomatic. In panel (C), line colour indicates SV callers. The results for COLO829 (left panel) and HCC1937 (right panel) cell lines are shown.

**Supplementary Figure S5**. Read depth and quality scores for somatic SNV calls. (A) Box plot showing read depth in tumour samples with varying tumour purity for TP (yellow) and FP (blue) SNVs called by ClairS and DeepSomatic for COLO829 and HCC1937 cell lines. (B) Box plot of QUAL scores from ClairS and DeepSomatic for TP and FP SNV calls in samples with varying tumour purity for COLO829 and HCC1937. The line in the box is the median of the data, the whiskers extend to the minimum and maximum values within 1.5 times the interquartile range (IQR) from the quartiles. Data points beyond the whiskers are shown as individual outliers. FP: false positive; TP: true positive; QUAL: quality.

**Supplementary Figure S6**. Boxplots of read depth and quality scores for TP and FP indel calls in tumour samples. (A) Read depth in tumour samples with varying tumour purity for TP (yellow) and FP (blue) indels called by ClairS and DeepSomatic for COLO829 and HCC1937 cell lines. (B) QUAL scores from ClairS and DeepSomatic for TP and FP indel calls in samples with varying tumour purity for COLO829 and HCC1937. The line in the box is the median of the data, the whiskers extend to the minimum and maximum values within 1.5 times the interquartile range (IQR) from the quartiles.

**Supplementary Figure S7**. Precision and recall of SV calling using four tools in samples with varied tumour purity for COLO829 and HCC1937. The bar height represents the total number of SV events detected by each tool. Bar colour indicates SV call concordance: blue for FP events; yellow for TP events; red for FN events. Precision and recall rates are labelled to the right and left of each bar, respectively.

**Supplementary Figure S8**. Variant allele frequency (VAF), read depth in tumour (DP), and quality score (QUAL) of FP and TP SNV calls from ClairS and DeepSomatic in COLO829 and HCC1937 samples with nine sequencing depth combinations. Panels are arranged with COLO829 on the left and HCC1937 on the right, and ClairS on the top and DeepSomatic on the bottom. Within each panel, results are further separated by sequencing depth combinations ranging from 15x–15x to 60x–60x (tumour—normal). FP calls are shown in blue and TP calls in yellow.

**Supplementary Figure S9**. Recall and precision of Indel calling across sequencing depths and tumour purities. Each panel displays the number of indels classified as true positives (TP, yellow), false positives (FP, blue), and false negatives (FN, red) across nine combinations of tumour-normal sequencing depths and four tumour purities (100, 80, 60, and 40%). Precision and recall are displayed to the right and left of each bar, respectively.

**Supplementary Figure S10**. Recall and precision of SV calling using four tools for nine different tumour-normal read depth combinations in COLO829 and HCC1937. Each of the eight blocks represents results from an SV caller for a cell line. Within each block, the *y*-axis indicates the read depth combinations, and the *x*-axis represents the number of discordant or concordant SV calls.

**Supplementary Figure S11**. The rainfall plots of FN, FP, and TP SNVs of ClairS in COLO829. Chromosomes 1–22 were listed as rows, with the ideogram shown at the top. The colour in the chromosome ideograms is shown in the key. The *x*-axis presents the position on each chromosome, and the *y*-axis presents the distance between an SNV and its neighbouring SNV. Points are coloured as FN (red), FP (blue), and TP (yellow). Cytogenetic bands (Stain): gneg (light), gpos25–100 (increasingly dark), acen (centromere, red), gvar (heterochromatic regions), and stalk (secondary constriction).

**Supplementary Figure S12**. The rainfall plots of FN, FP, and TP SNVs of ClairS in HCC1937. Chromosomes 1–22 were listed as rows, with the ideogram shown at the top. The colour in the chromosome ideograms is shown in the key. The *x*-axis presents the position on each chromosome, and the *y*-axis presents the distance between an SNV and its neighbouring SNV. Points are coloured as FN (red), FP (blue), and TP (yellow). Cytogenetic bands (Stain): gneg (light), gpos25–100 (increasingly dark), acen (centromere, red), gvar (heterochromatic regions), and stalk (secondary constriction).

**Supplementary Figure S13**. The rainfall plots of FN, FP, and TP SNVs of DeepSomatic in COLO829. Chromosomes 1–22 were listed as rows, with the ideogram shown at the top. The colour in the chromosome ideograms is shown in the key. The *x*-axis presents the position on each chromosome, and the y-axis presents the distance between an SNV and its neighbouring SNV. Points are coloured as FN (red), FP (blue), and TP (yellow). Cytogenetic bands (Stain): gneg (light), gpos25–100 (increasingly dark), acen (centromere, red), gvar (heterochromatic regions), and stalk (secondary constriction).

**Supplementary Figure S14**. The rainfall plots of FN, FP, and TP SNVs of DeepSomatic in HCC1937. Chromosomes 1–22 were listed as rows, with the ideogram shown at the top. The colour in the chromosome ideograms is shown in the key. The *x*-axis presents the position on each chromosome, and the *y*-axis presents the distance between an SNV and its neighbouring SNV. Points are coloured as FN (red), FP (blue), and TP (yellow). Cytogenetic bands (Stain): gneg (light), gpos25–100 (increasingly dark), acen (centromere, red), gvar (heterochromatic regions), and stalk (secondary constriction).

**Supplementary Figure S15**. The proportion of false-positive variants that overlap with different genome stratifications across tumour purities. Four types of genome stratifications were examined for SNVs (A) and indels (B) called by ClairS and DeepSomatic in COLO829: segmental duplications, tandem repeats, short read low mappability regions, and abnormal GC% regions. The colour of tiles represents the proportion of variants that overlap with specific genome stratifications.

**Supplementary Figure S16**. The proportion of false-positive SVs that overlap with different genome stratifications in samples with 100% tumour purity. SVs specific to LRS are termed false-positive events compared to short reads. Genome stratifications were labelled on the *x*-axis with results from four tools listed on the horizontal panels for COLO829 (left) and HCC1937 (right). Bars in teal represent the SV proportions that overlap with specific genome stratifications.

**Supplementary Figure S17**. Germline leakage rate across tumour purity and sequencing depth. (A) The germline leakage rate in samples of the COLO829 and HCC1937 cell lines with tumour purity from 100% to 10% for ClairS and DeepSomatic. (B) The germline leakage rate in variant calls with different tumour and normal read depth combinations for ClairS and DeepSomatic in COLO829 and HCC1937. Only tumour samples with 100% tumour purity were shown in the plot.

**Supplementary Figure S18**. Benchmarking SV detection using the public COLO829 gold standard. (A) Heatmap showing the 68 gold standard SVs (*x*-axis) that were detected or missed by four tools across different tumour purities, with colours indicating the SV types. (B) Bar plots display the recall and precision rates calculated for each tool under different tumour purities, with colours indicating the FP, FN, or TP.

**Supplementary Table S1**. Comparison of LRS alignments between CHM13-T2T and GRCh38 human reference assemblies.

**Supplementary Table S2**. SNV detection of intersections and unions between ClairS and DeepSomatic across tumour purity.

**Supplementary Table S3**. The somatic SNV calling performance on chromosome 22 using ClairS and DeepSomatic.

## Supplementary Material

giag037_Supplemental_Files

giag037_Authors_Response_To_Reviewer_Comments_original_submission

giag037_GIGA-D-25-00177_original_submission

giag037_GIGA-D-25-00177_revision_1

giag037_GIGA-D-25-00177_revision_2

giag037_Reviewer_1_Report_original_submission Reviewer 1 -- 7/3/2025

giag037_Reviewer_2_Report_original_submission Reviewer 2 -- 7/31/2025

giag037_Reviewer_2_Report_revision_1 Reviewer 2 -- 11/3/2025

giag037_Reviewer_2_Report_revision_2 Reviewer 2 -- 2/11/2026

## Data Availability

All additional supporting data are available in the *GigaScience* repository, GigaDB [87]. The BAM files for cell line mixtures are available from the European Genome-Phenome Archive (EGA) under accession number EGAD00001015628 under study EGAS00001008107. Raw POD5 files are available on request.
