# Supplementary material for: Cancer genome standards for long-read sequencing using cancer cell line mixtures
Source: Gigascience. 2026 Apr 3;15:giag037. doi: 10.1093/gigascience/giag037 (PMC13137868; doi:10.1093/gigascience/giag037)
Supplement: giag037_Supplemental_Files [file giag037_supplemental_files.zip › Supplementary figures_revision_Clean.docx]

**Supplementary Figures**


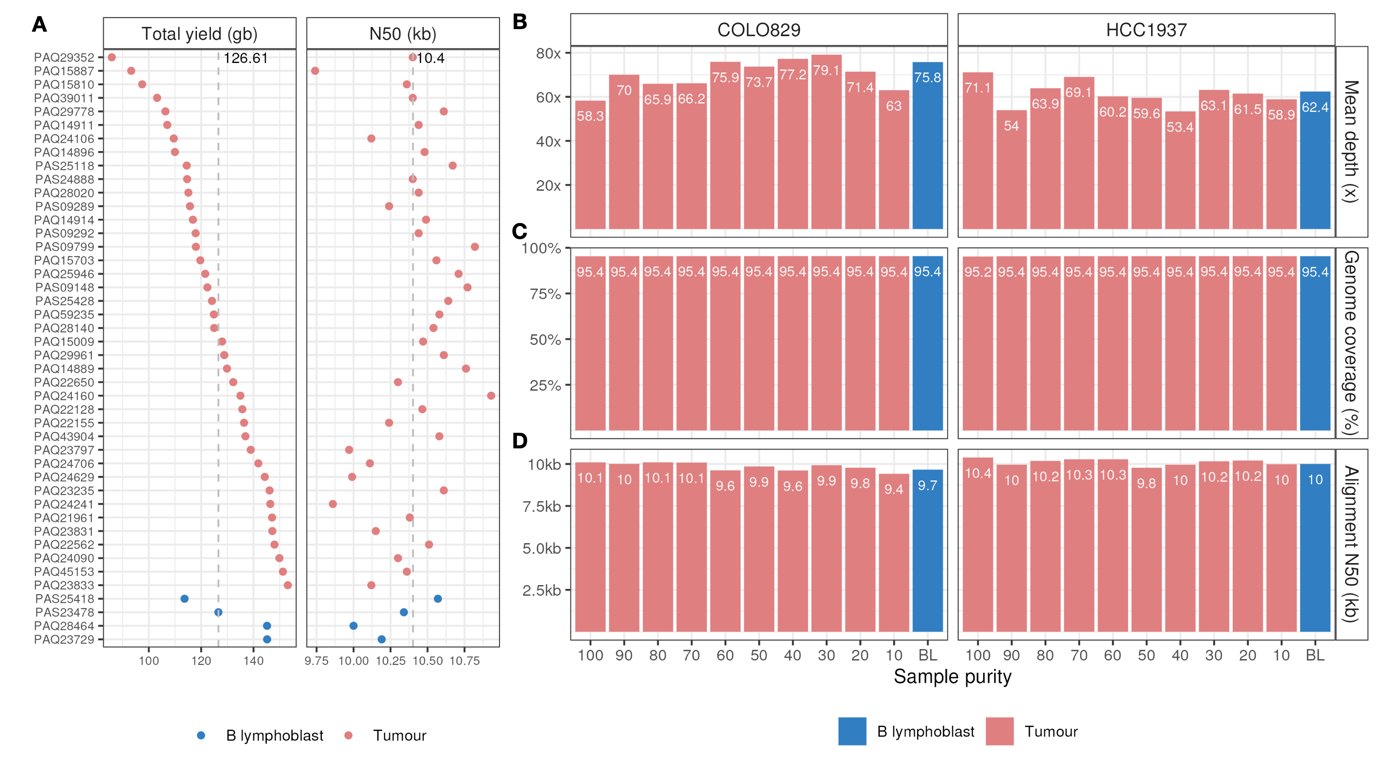


**Supplementary Figure S1. The quality of sequence data and alignment for tumour mixtures and matched normal samples. A)** Dot plot showing the total data yield (gb, gigabases) and read length N50 kb(kilobases) for each flow cell. Samples are ordered by sample type, then gb of sequence. **B)** Total sequencing depth for each sample, with the x axis showing the sample tumour purity and the y axis indicating the sequence read depth. **C)** The proportion of genome assembly covered by at least one read in each sample. **D)** Alignment length N50 for each sample. Samples are coloured by type (blue: B lymphoblast non-tumour and red: tumour mixture).


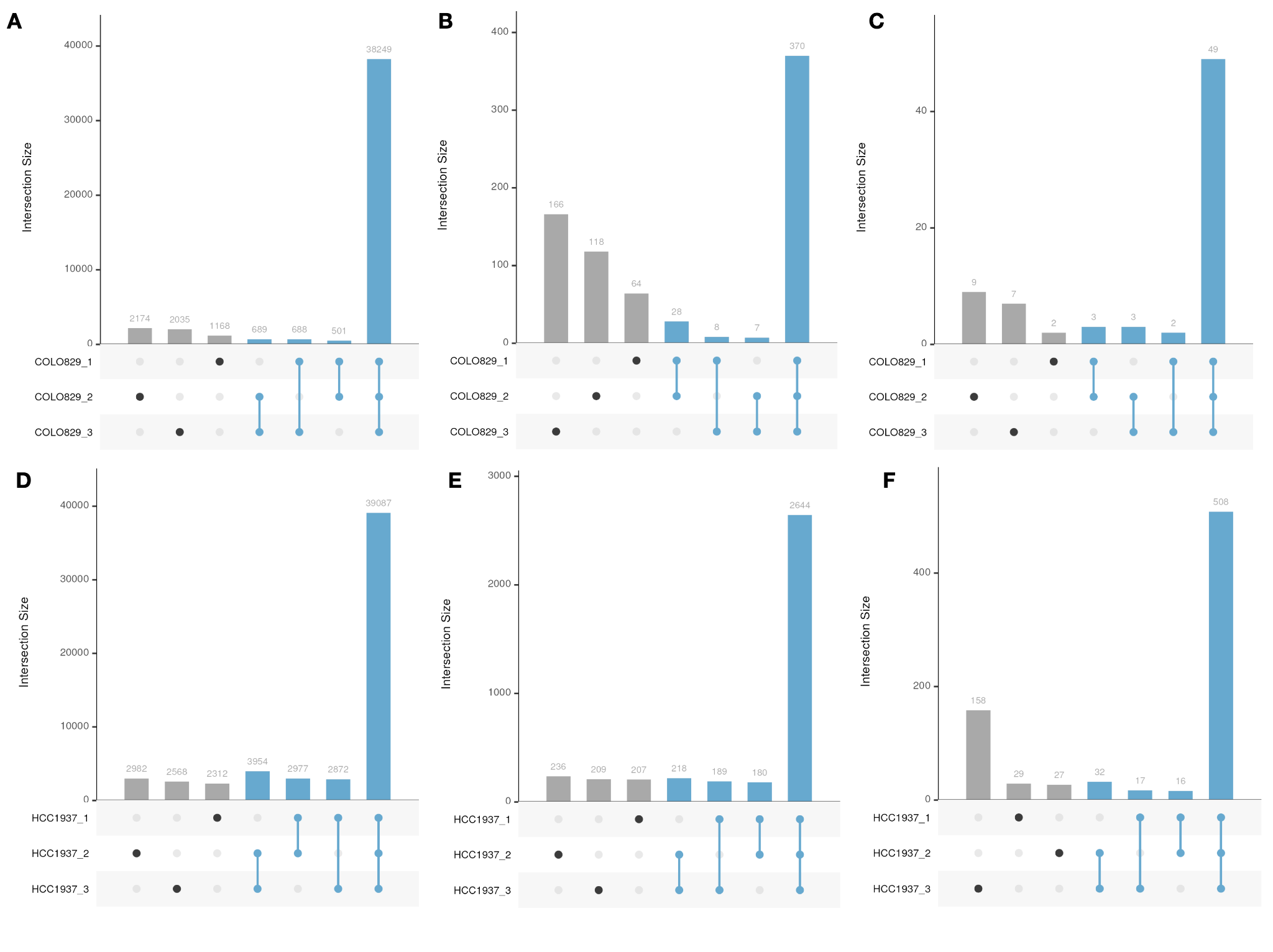


**Supplementary Figure S2. Creation of short-read “gold standard” somatic mutation calls for the COLO829 and HCC1937 cell lines.** The COLO829 and HCC1973 tumour-derived cell lines and their matched non-tumour cell lines were sequenced in triplicate in short reads. For COLO829, the UpSet plots show the number of somatic A) single-nucleotide variants (SNVs), B) insertion and deletion (indels), and C) structural variant (SV) events detected uniquely in each replicate and shared by three or two replicates. For HCC1937, UpSet plots show the number of somatic D) SNVs, E) indels, and F) SV events detected uniquely in each replicate and shared by three or two biological replicates. Blue bars represent events shared by at least two replicates, which were used to define the gold standard.


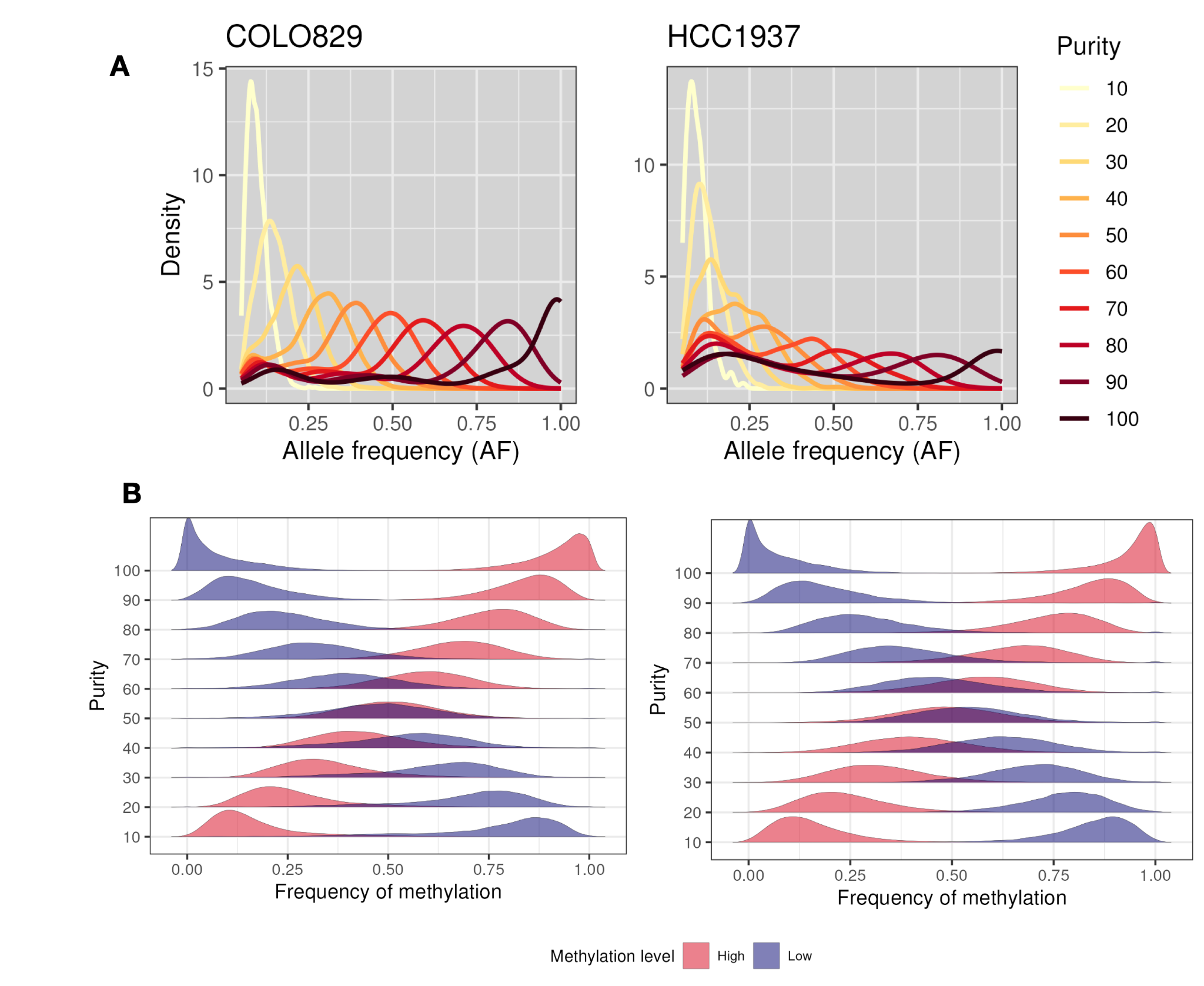


**Supplementary Figure S3. The distribution of variant allele frequency (AF) and methylation in samples with different tumour purities. A)** The distribution of variant allele frequency of SNVs at the genomic regions with a copy number equal to two in COLO829 and HCC1937. The lines are coloured by tumour purity from 100% to 10%. **B)** The methylation frequency distribution in CpG sites (x-axis) in tumour samples with different tumour purities (y-axis) for COLO829 (left) and HCC1937 (right). Only the CpG sites with differential methylation frequency between the 100% tumour and blood lymphocyte cell lines are included in the plot. Plots are coloured by the methylation status in the 100% tumour sample with a high (red) or low (blue) methylation level.


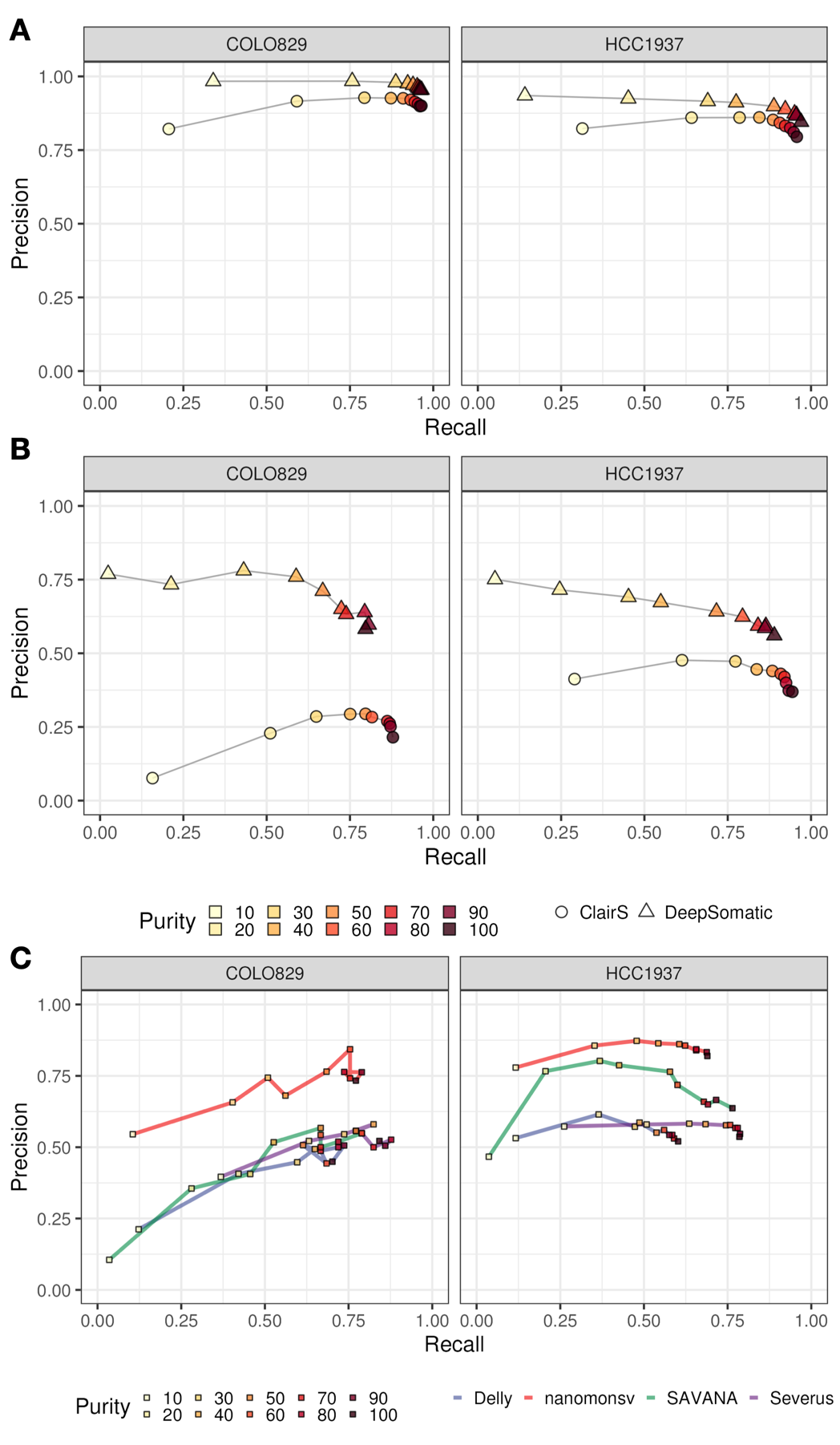


**Supplementary Figure S4. Precision-recall curves (AUPRC) for variant detection across different tumour purities.** The precision (y-axis) and recall (x-axis) of long read calls against the short read “gold standard” are shown for SNVs (A), indels (B) and SVs (B). Points are coloured by tumour purity. In panel A and B, Points in circles shape represent ClairS results and triangles represent DeepSomatic. In panel C, line colour indicates SV callers. The results for COLO829 (left panel) and HCC1937 (right panel) cell lines are shown.


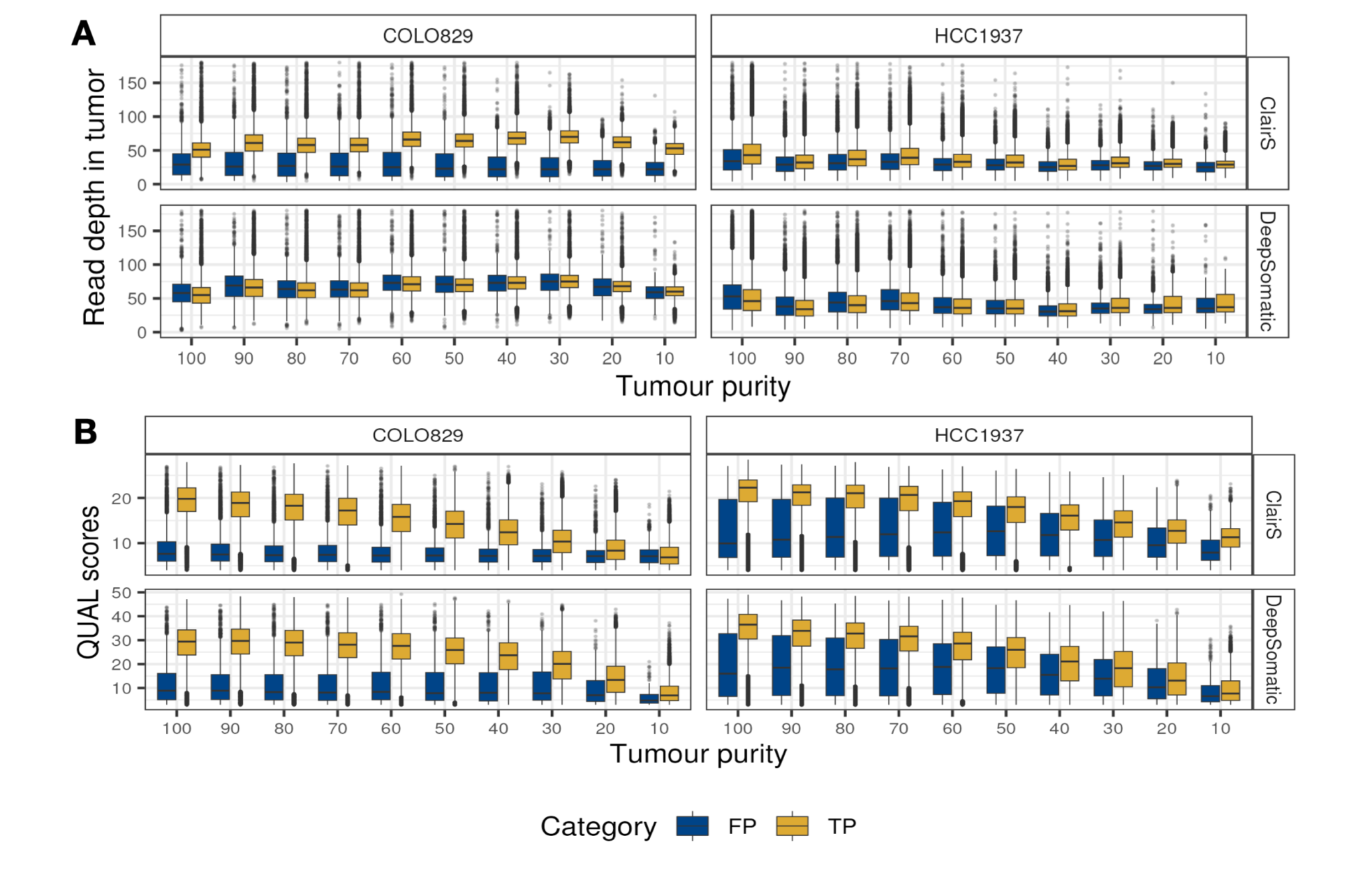


**Supplementary Figure S5. Read depth and quality scores for somatic SNV calls.** a) Box plot showing read depth in tumour samples with varying tumour purity for TP (yellow) and FP (blue) SNVs called by ClairS and DeepSomatic for COLO829 and HCC1937 cell lines. b) Box plot of QUAL scores from ClairS and DeepSomatic for TP and FP SNV calls in samples with varying tumour purity for COLO829 and HCC1937. The line in the box is the median of the data, the whiskers extend to the minimum and maximum values within 1.5 times the interquartile range (IQR) from the quartiles. Data points beyond the whiskers are shown as individual outliers. FP: false positive; TP: true positive; QUAL: quality.
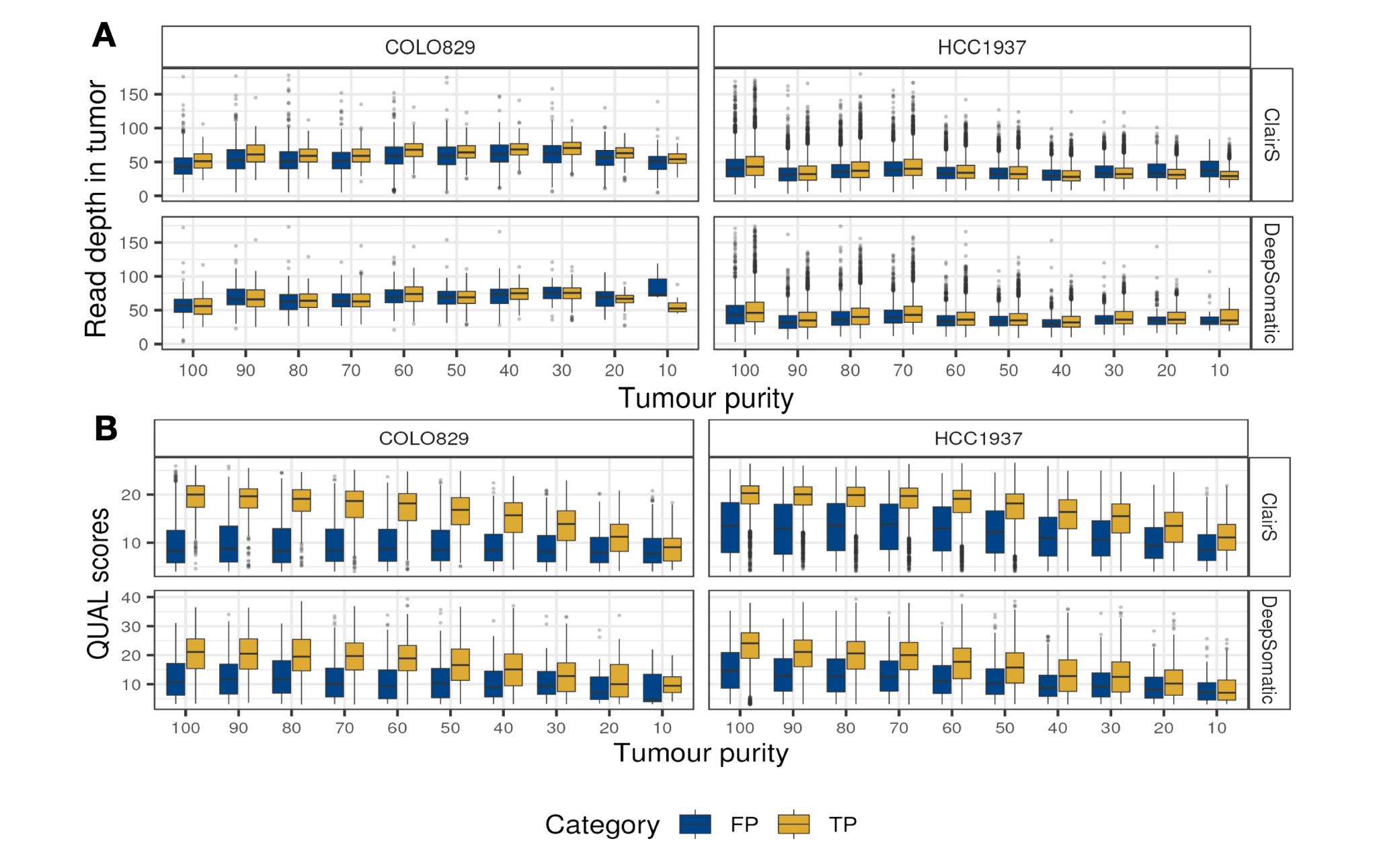


**Supplementary Figure S6. Boxplots of read depth and quality scores for TP and FP indel calls in tumour samples.** a) Read depth in tumour samples with varying tumour purity for TP (yellow) and FP (blue) indels called by ClairS and DeepSomatic for COLO829 and HCC1937 cell lines. b) QUAL scores from ClairS and DeepSomatic for TP and FP indel calls in samples with varying tumour purity for COLO829 and HCC1937. The line in the box is the median of the data, the whiskers extend to the minimum and maximum values within 1.5 times the interquartile range (IQR) from the quartiles.


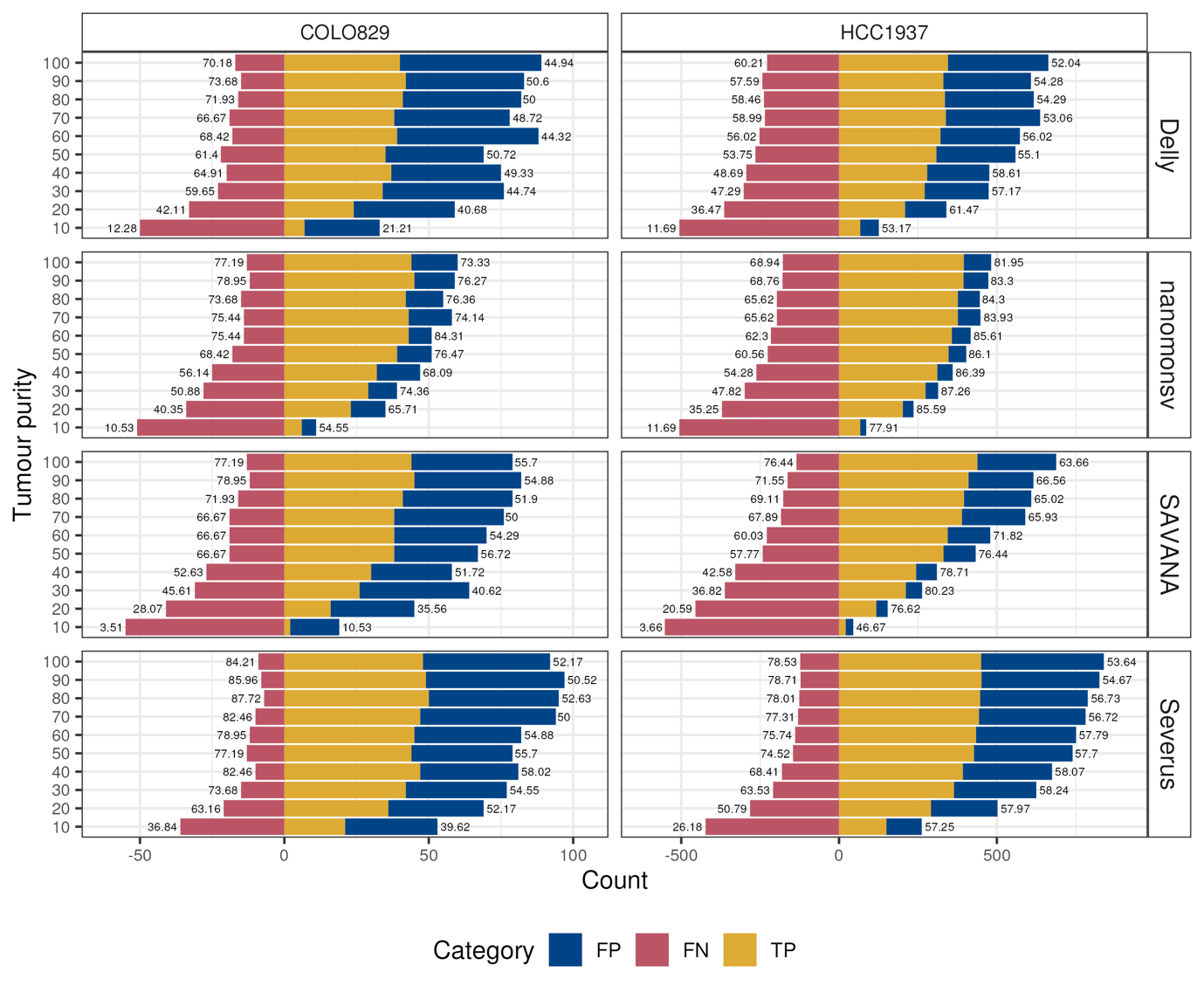


**Supplementary Figure S7. Precision and recall of SV calling using four tools in samples with varied tumour purity for COLO829 and HCC1937.** The bar height represents the total number of SV events detected by each tool. Bar colour indicates SV call concordance: blue for FP events; yellow for TP events; red for FN events. Precision and recall rates are labelled to the right and left of each bar, respectively.


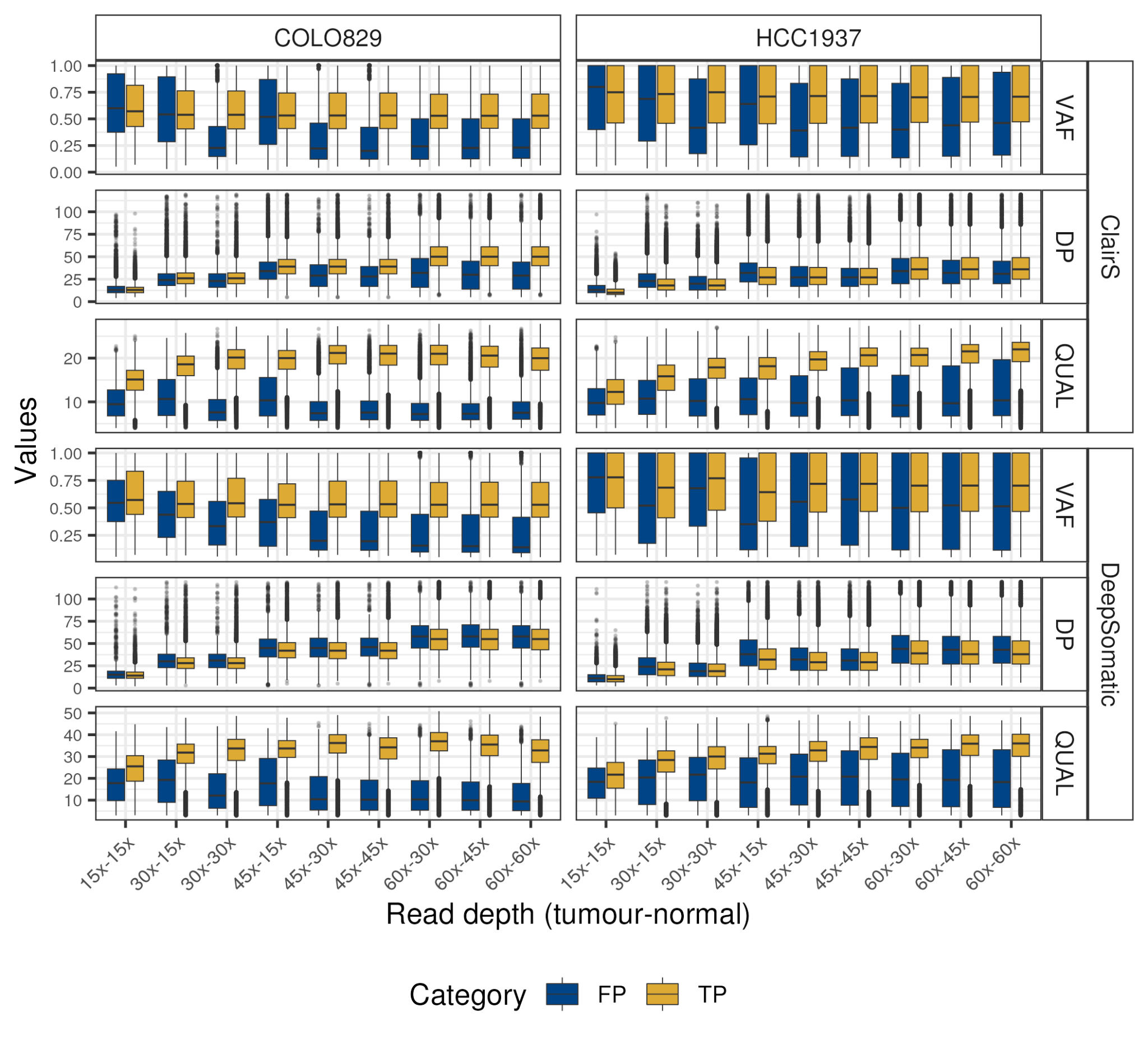


**Supplementary Figure S8. Variant allele frequency (VAF), read depth in tumour (DP) and quality score (QUAL) of FP and TP SNVs calls from ClairS and DeepSomatic in COLO829 and HCC1937 samples with nine sequencing depth combinations.** Panels are arranged with COLO829 on the left and HCC1937 on the right, and ClairS on the top and DeepSomatic on the bottom. Within each panel, results are further separated by sequencing depth combinations ranging from 15x -15x to 60x - 60x (tumour - normal). FP calls are shown in blue and TP calls in yellow.


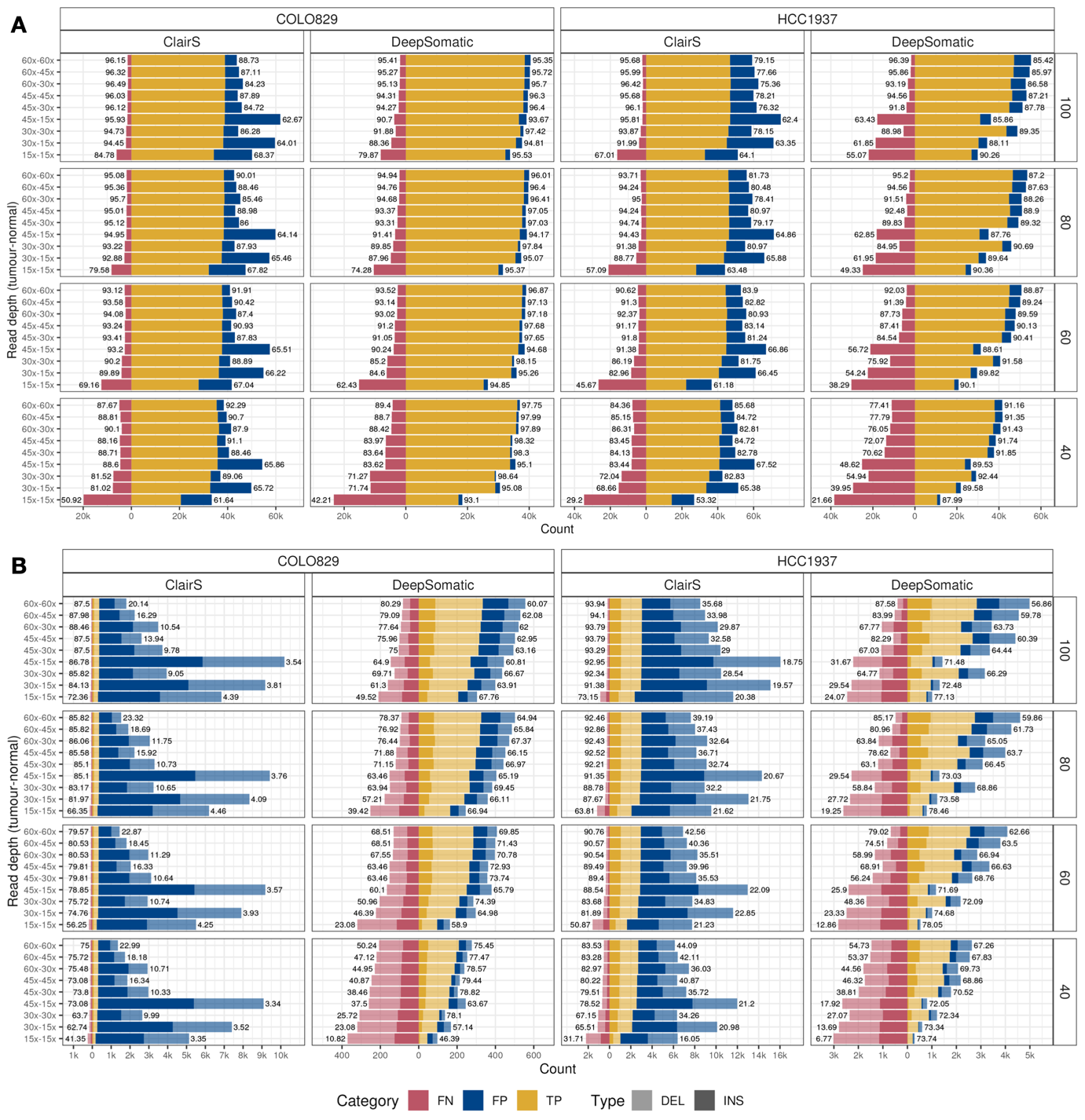


**Supplementary Figure S9. Recall and precision of Indel calling across sequencing depths and tumour purities.** Each panel displays the number of indels classified as true positives (TP, yellow), false positives (FP, blue), and false negatives (FN, red) across nine combinations of tumour-normal sequencing depths and four tumour purities (100, 80, 60 and 40%). Precision and recall are displayed to the right and left of each bar, respectively.


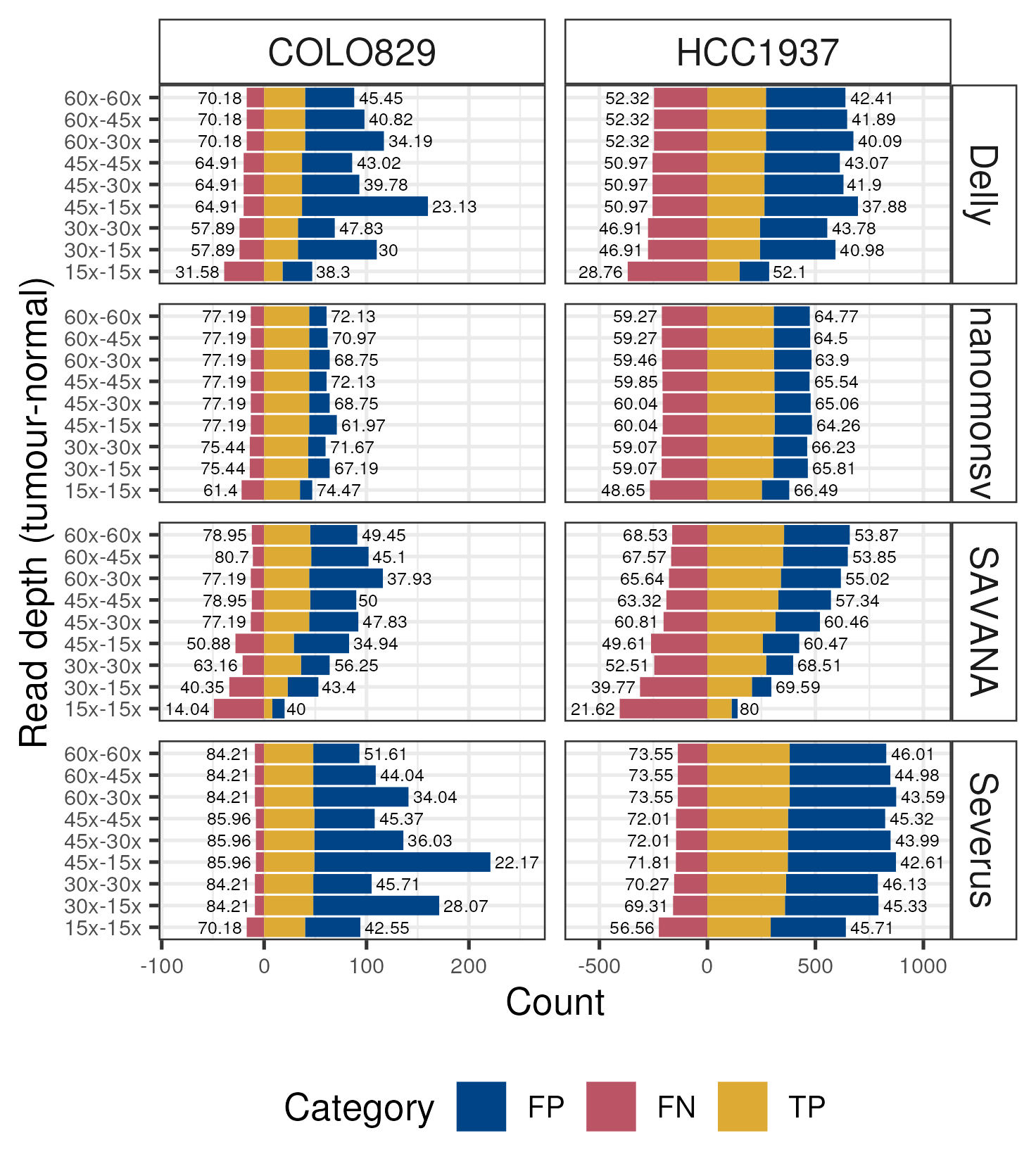


**Supplementary Figure S10. Recall and precision of SV calling using four tools for nine different tumour-normal read depth combinations in COLO829 and HCC1937.** Each of the eight blocks represents results from an SV caller for a cell line. Within each block, the y-axis indicates the read depth combinations and the x-axis represents the number of discordant or concordant SV calls.


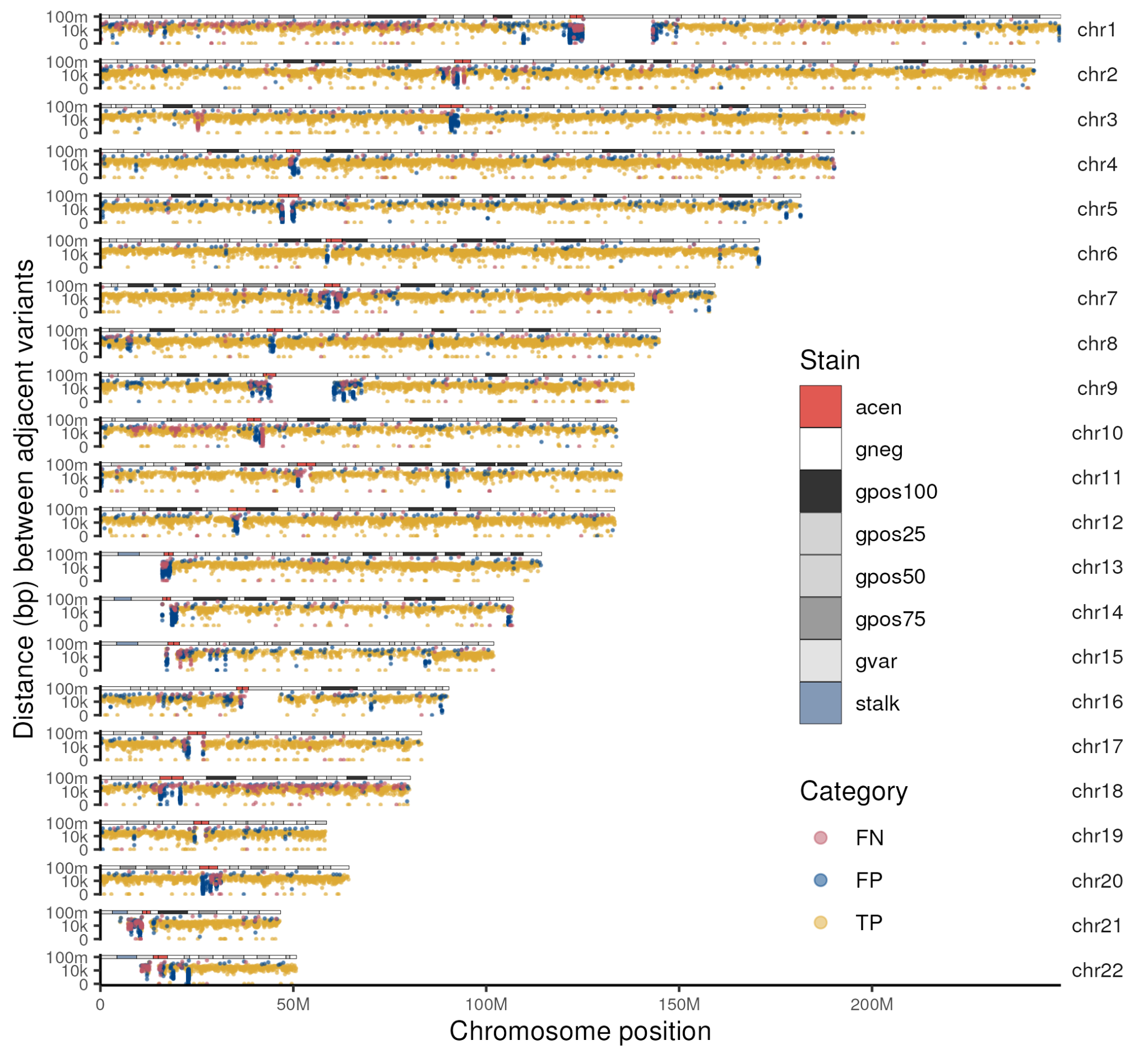


**Supplementary Figure S11**. **The rainfall plots of FN, FP and TP SNVs of ClairS in COLO829.** Chromosomes 1 to 22 were listed as rows, with the ideogram shown at the top. The colour in the chromosome ideograms is shown in the key. The x-axis presents the position on each chromosome, and the y-axis presents the distance between an SNV and its neighbouring SNV. Points are coloured as FN (red), FP (blue) and TP (yellow). Cytogenetic bands (Stain): gneg (light), gpos25–100 (increasingly dark), acen (centromere, red), gvar (heterochromatic regions), stalk (secondary constriction).


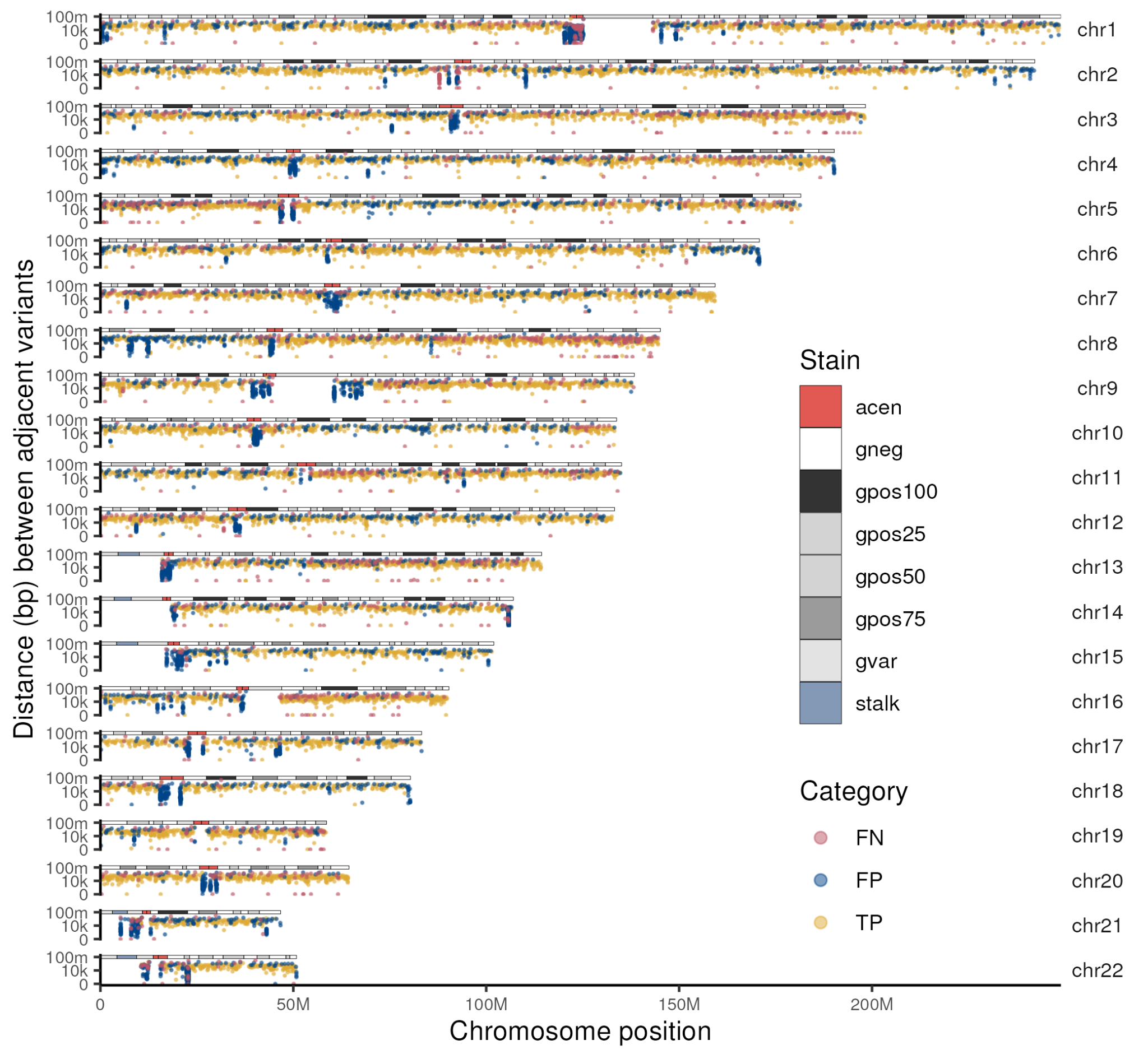


**Supplementary Figure S12.** **The rainfall plots of FN, FP and TP SNVs of ClairS in HCC1937.** Chromosomes 1 to 22 were listed as rows, with the ideogram shown at the top. The colour in the chromosome ideograms is shown in the key. The x-axis presents the position on each chromosome, and the y-axis presents the distance between an SNV and its neighbouring SNV. Points are coloured as FN (red), FP (blue) and TP (yellow). Cytogenetic bands (Stain): gneg (light), gpos25–100 (increasingly dark), acen (centromere, red), gvar (heterochromatic regions), stalk (secondary constriction).

**
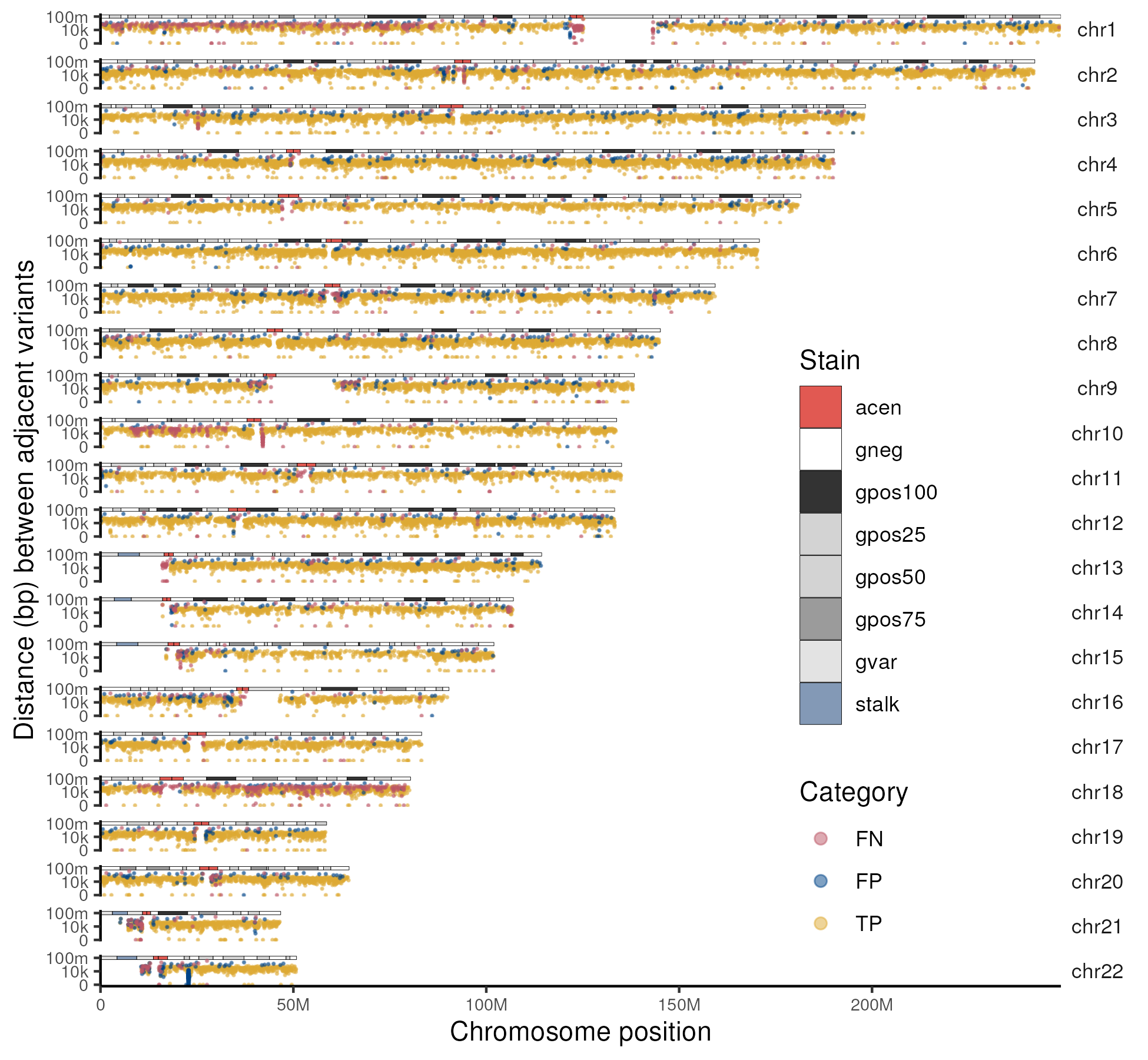
**

**Supplementary Figure S13. The rainfall plots of FN, FP and TP SNVs of DeepSomatic in COLO829.** Chromosomes 1 to 22 were listed as rows, with the ideogram shown at the top. The colour in the chromosome ideograms is shown in the key. The x-axis presents the position on each chromosome, and the y-axis presents the distance between an SNV and its neighbouring SNV. Points are coloured as FN (red), FP (blue) and TP (yellow). Cytogenetic bands (Stain): gneg (light), gpos25–100 (increasingly dark), acen (centromere, red), gvar (heterochromatic regions), stalk (secondary constriction).

**
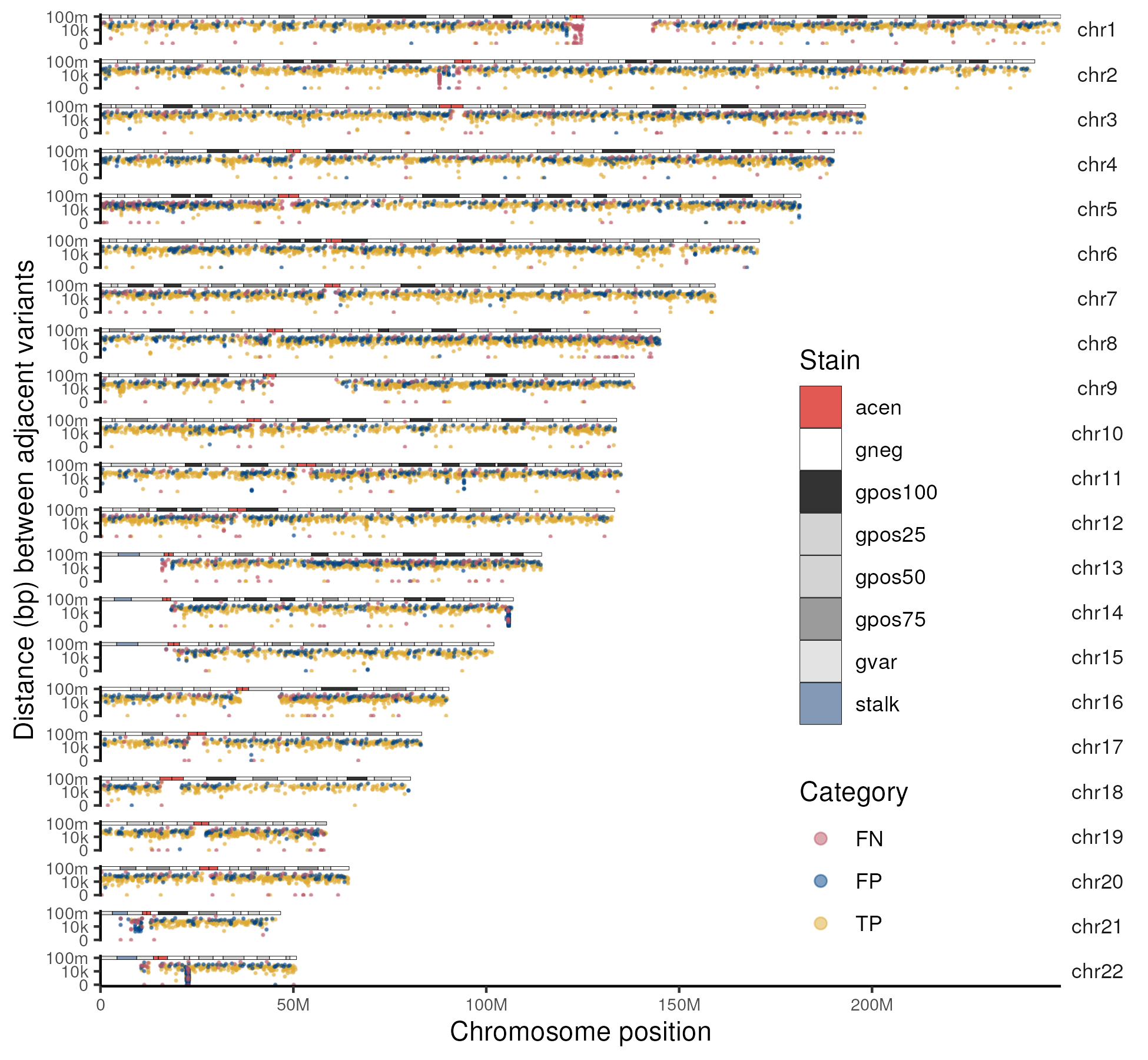
**

**Supplementary Figure S14. The rainfall plots of FN, FP and TP SNVs of DeepSomatic in HCC1937.** Chromosomes 1 to 22 were listed as rows, with the ideogram shown at the top. The colour in the chromosome ideograms is shown in the key. The x-axis presents the position on each chromosome, and the y-axis presents the distance between an SNV and its neighbouring SNV. Points are coloured as FN (red), FP (blue) and TP (yellow). Cytogenetic bands (Stain): gneg (light), gpos25–100 (increasingly dark), acen (centromere, red), gvar (heterochromatic regions), stalk (secondary constriction).


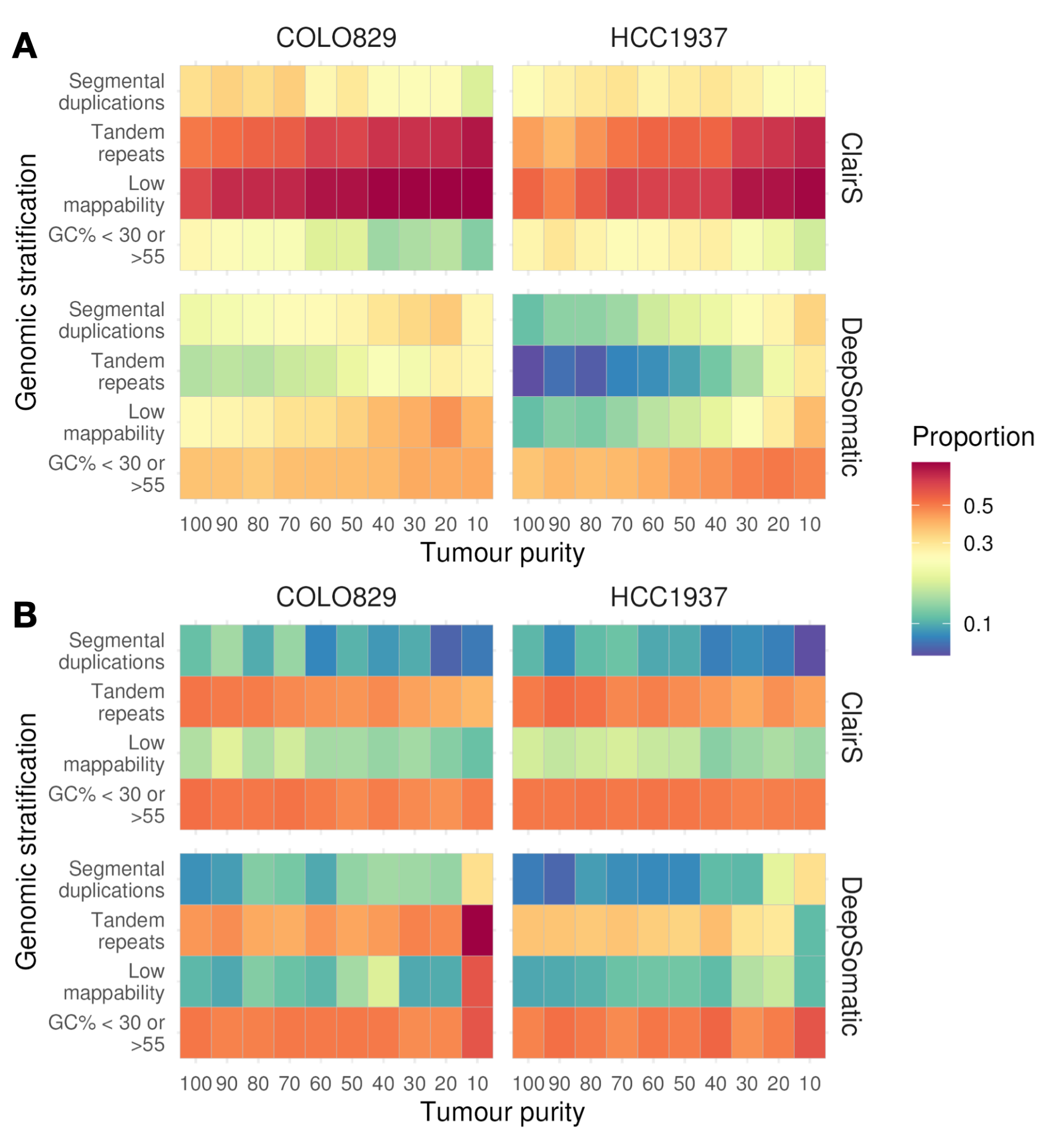


**Supplementary Figure S15. The proportion of false-positive variants that overlap with different genome stratifications across tumour purities.** Four types of genome stratifications were examined for SNVs (A) and indels (B) called by ClairS and DeepSomatic in COLO829: Segmental duplications, tandem repeats, short read low mappability regions, and abnormal GC% regions. The colour of tiles represents the proportion of variants that overlap with specific genome stratifications.


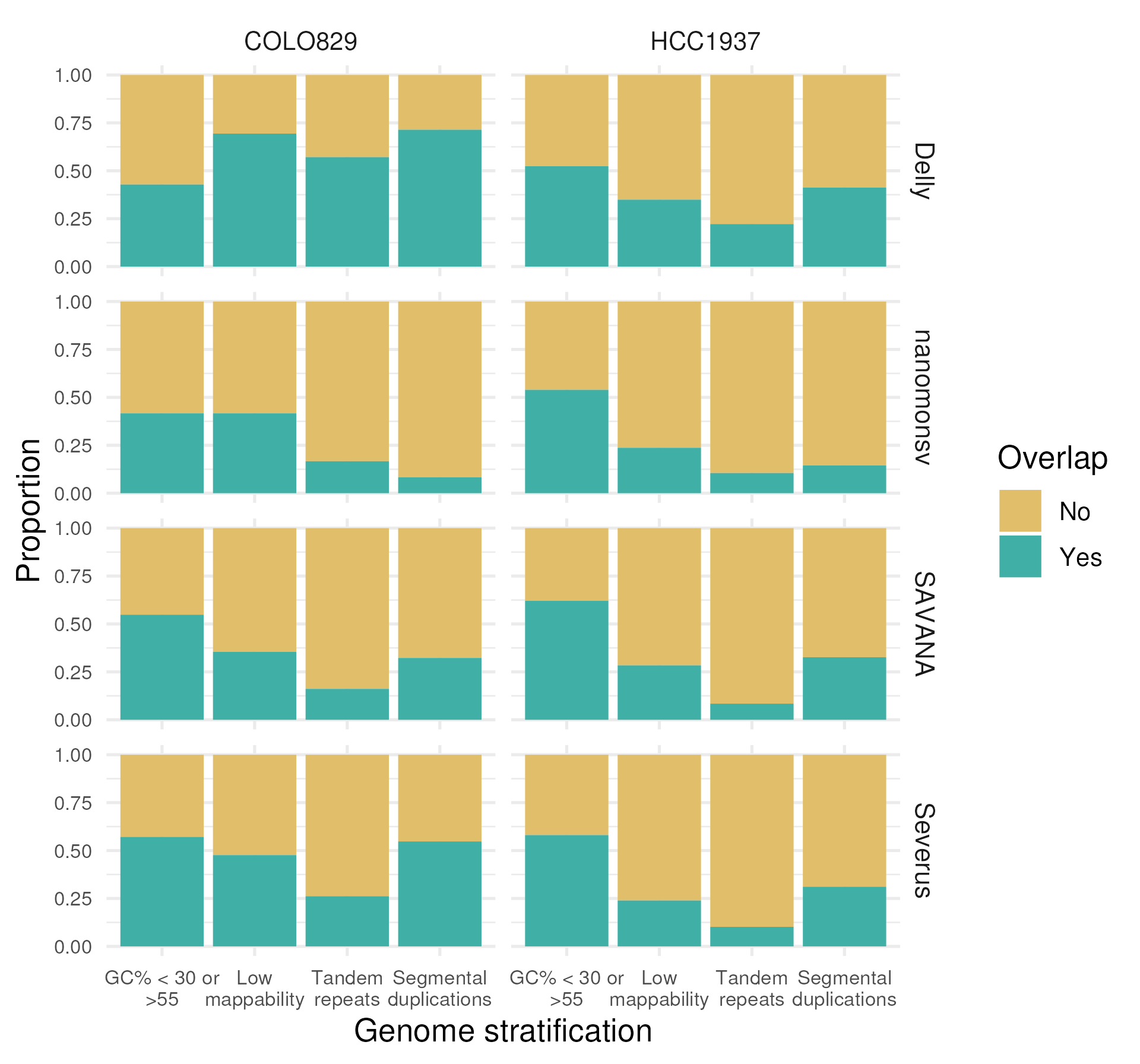


**Supplementary Figure S16. The proportion of false-positive SVs that overlap with different genome stratifications in samples with 100% tumour purity.** SVs specific to LRS are termed false-positive events compared to short read. Genome stratifications were labelled on the x-axis with results from four tools listed on the horizontal panels for COLO829 (left) and HCC1937 (right). Bars in teal represent the SV proportions that overlap with specific genome stratifications.


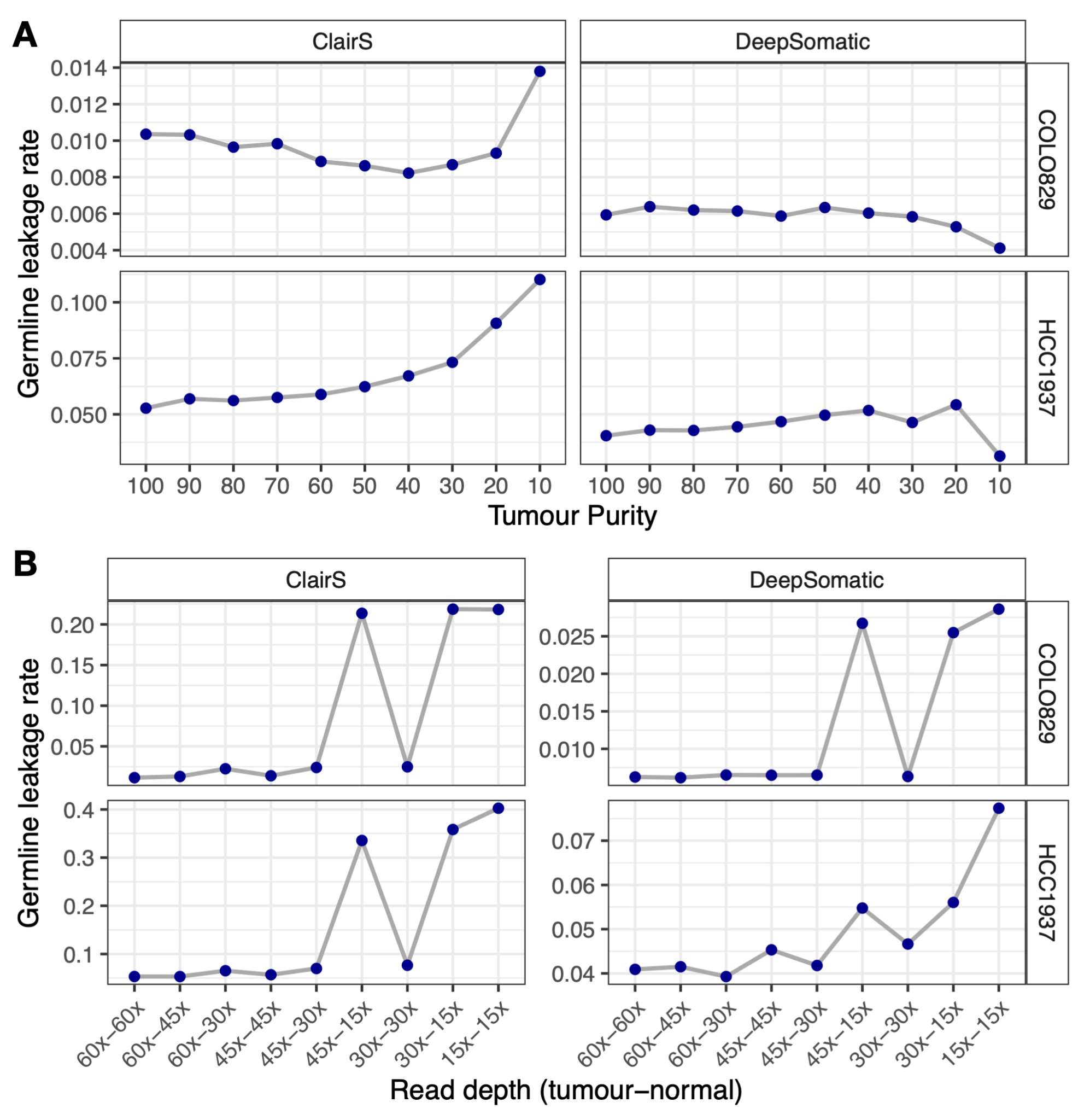


**Supplementary Figure S17. Germline leakage rate across tumour purity and sequencing depth.** (A) The germline leakage rate in samples of the COLO829 and HCC1937 cell lines with tumour purity from 100% to 10% for ClairS and DeepSomatic. (B) The germline leakage rate in variant calls with different tumour and normal read depth combinations for ClairS and DeepSomatic in COLO829 and HCC1937. Only tumour samples with 100% tumour purity were shown in the plot.


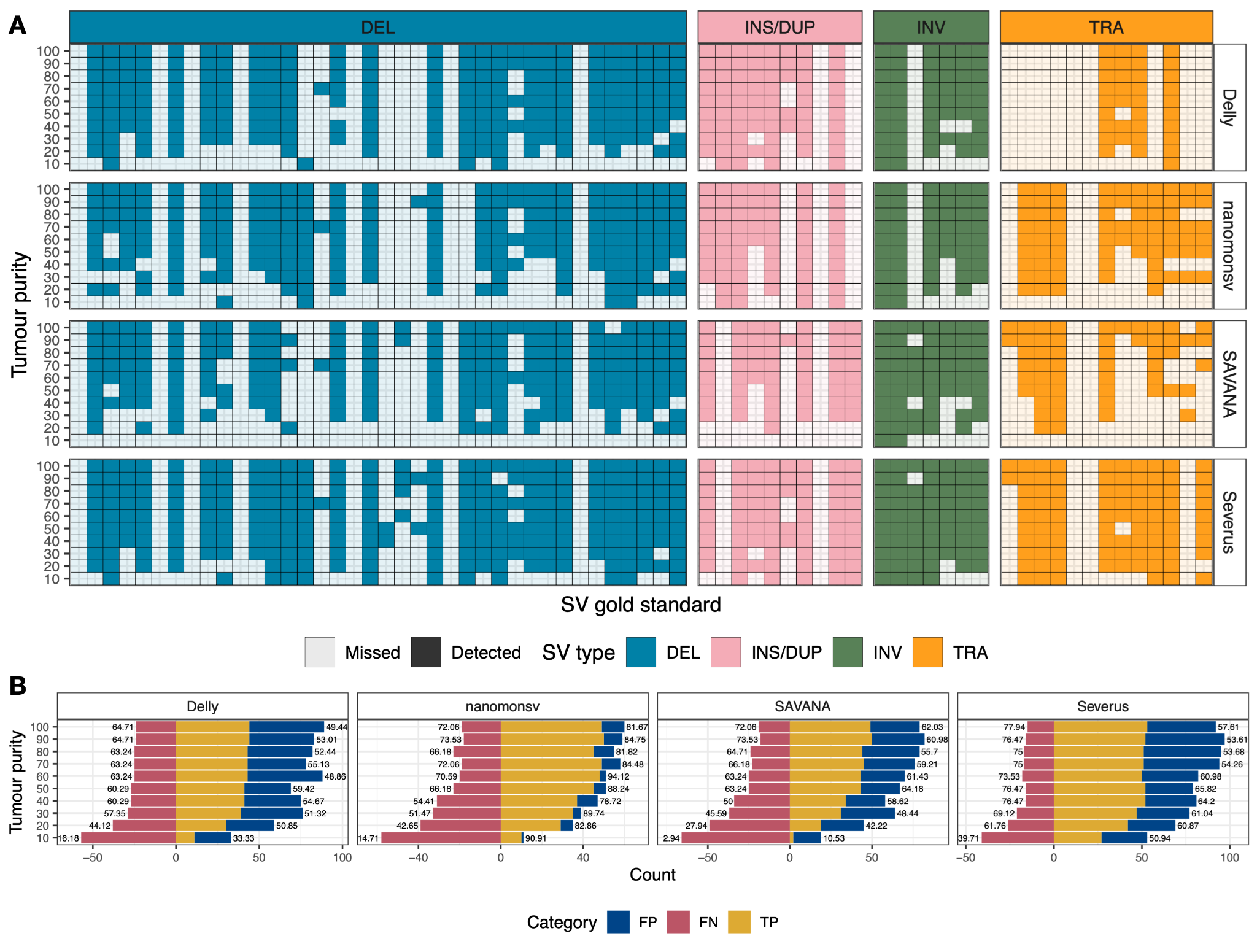


**Supplementary Figure S18.** **Benchmarking SV detection using the public COLO829 gold standard. a.** Heatmap showing the 68 gold standard SVs (x-axis) that were detected or missed by four tools across different tumour purities, with colours indicating the SV types. **b.** Bar plots display the recall and precision rates calculated for each tool under different tumour purities, with colours indicating the FP, FN or TP.
