# Supplementary material for: Cancer genome standards for long-read sequencing using cancer cell line mixtures
Source: Gigascience. 2026 Apr 3;15:giag037. doi: 10.1093/gigascience/giag037 (PMC13137868; doi:10.1093/gigascience/giag037)
Supplement: giag037_Supplemental_Files [file giag037_supplemental_files.zip › Supplementary Tables.pdf]

Supplementary Table S1. Comparison of LRS alignments between CHM13-T2T and GRCh38 human reference assembly

Assembly	Sample	Sequence Divergence	Mapping Rate (%)
CHM13-T2T	COLO829	0.0201259	99.86%
GRCh38	COLO829	0.0310062	98.31%
CHM13-T2T	COLO829_BL	0.0184784	99.84%
GRCh38	COLO829_BL	0.0275728	98.53%
CHM13-T2T	HCC1937	0.0165879	99.91%
GRCh38	HCC1937	0.0238883	99.03%
CHM13-T2T	HCC1937_BL	0.0170962	99.82%
GRCh38	HCC1937_BL	0.0254134	98.77%

Supplementary Table S2. SNV detection of intersections and unions between ClairS and DeepSomatic across tumour purity

Sample	Intersected SNV	ClairS Unique	DeepSomatic Unique	Total SNV	Intersected Precision	Intersected Recall	Union Recall	Tumour Purity (%)	% SNVs in Intersection
COLO829_10	7276	2797	6582	16655	0.99	0.18	0.37	10	43.69
COLO829_20	23337	2532	7527	33396	0.99	0.58	0.77	20	69.88
COLO829_30	31897	2423	4453	38773	0.99	0.79	0.89	30	82.27
COLO829_40	35250	2571	2708	40529	0.99	0.87	0.93	40	86.97
COLO829_50	36837	2585	1987	41409	0.98	0.90	0.95	50	88.96
COLO829_60	37851	2781	1675	42307	0.98	0.93	0.96	60	89.47
COLO829_70	38472	3030	1395	42897	0.98	0.94	0.96	70	89.68
COLO829_80	38923	3275	1253	43451	0.98	0.95	0.97	80	89.58
COLO829_90	39274	3562	1301	44137	0.97	0.95	0.97	90	88.98
COLO829	39491	3487	960	43938	0.97	0.95	0.97	100	89.88
HCC1937_10	5969	12671	1416	20056	0.94	0.12	0.34	10	29.76
HCC1937_20	21423	15047	2459	38929	0.93	0.41	0.69	20	55.03
HCC1937_30	34108	10531	2734	47373	0.92	0.64	0.83	30	72.00
HCC1937_40	39632	8353	1951	49936	0.92	0.75	0.87	40	79.37
HCC1937_50	45731	5091	2624	53446	0.91	0.85	0.92	50	85.56
HCC1937_60	47982	4623	2801	55406	0.90	0.89	0.94	60	86.60
HCC1937_70	49754	4424	3418	57596	0.90	0.91	0.96	70	86.38
HCC1937_80	50908	4651	2888	58447	0.89	0.93	0.96	80	87.10
HCC1937_90	51777	5423	2108	59308	0.89	0.94	0.97	90	87.30
HCC1937	53169	5700	2918	61787	0.88	0.95	0.98	100	86.05
