# Supplementary material for: Cancer genome standards for long-read sequencing using cancer cell line mixtures
Source: Gigascience. 2026 Apr 3;15:giag037. doi: 10.1093/gigascience/giag037 (PMC13137868; doi:10.1093/gigascience/giag037)
Supplement: giag037_GIGA-D-25-00177_revision_1 [file giag037_giga-d-25-00177_revision_1.pdf]

GigaScience

Cancer genome standards for long-read sequencing using cancer cell line mixtures

--Manuscript Draft--

**Manuscript Number:**	GIGA-D-25-00177R1
**Full Title:**	Cancer genome standards for long-read sequencing using cancer cell line mixtures
**Article Type:**	Research
**Funding Information:**	National Health and Medical Research Council (2018244) — Dr Nicola Waddell<br>Cancer Council Queensland (000000027) — Dr Nicola Waddell<br>Ian Potter Foundation — Mr John V. Pearson<br>Australian Cancer Research Foundation — Mr John V. Pearson, Dr Nicola Waddell
**Abstract:**	Long-read sequencing (LRS) improves genome alignment and facilitates resolving variants in genomic regions of low complexity, making it a promising approach for cancer variant detection and biomarker discovery. Here, we evaluate the performance of LRS using Oxford Nanopore sequencing to detect somatic variants across different tumour purities and sequencing depths by comparing to somatic variants from short-read sequencing. We generated experimental mixtures of cancer cell lines and matched normal cell lines to simulate 10 tumour purities (ranging from 0 to 100% tumour content). This resulted in 22 samples which were sequenced using whole genome LRS to a targeted depth of 60x and somatic variants identified. We down-sampled data to explore the optimal read depth for somatic variant detection. Our results show long-read variant calling tools achieve recall rates comparable to short-read gold standards. While tumour sequencing depth of 30x to 60x is generally sufficient for detecting common variants, particularly structural variants, sequencing the matched normal sample at adequate depth is crucial for accuracy. Notably, we found variants unique to LRS that may represent real and previously undetected events. This study highlights key factors for optimising cancer genome sequencing with LRS and provides a comprehensive dataset of cell line mixtures for future research and tool development.
**Corresponding Author:**	Jia Zhang, Ph.D.<br>QIMR Berghofer: QIMR Berghofer Medical Research Institute<br>Brisbane, QLD AUSTRALIA
**Corresponding Author Secondary Information:**	
**Corresponding Author's Institution:**	QIMR Berghofer: QIMR Berghofer Medical Research Institute
**Corresponding Author's Secondary Institution:**	
**First Author:**	Jia Zhang, Ph.D.
**First Author Secondary Information:**	
**Order of Authors:**	Jia Zhang, Ph.D.
	Ho Yi Wong, Ph.D.
	Lingchen Liu
	Lambros T. Koufariotis
	Scott Wood
	Nadine Fitzpatrick
	Jenny Quiatchon

Powered by Editorial Manager® and ProduXion Manager® from Aries Systems Corporation

	Paul Collins
	John V. Pearson
	Nicola Waddell
**Order of Authors Secondary Information:**	
**Response to Reviewers:**	Thank you for the time and effort you spent on this manuscript. We would like to thank both referees for their thoughtful, in-depth reviews which have helped improve our manuscript. Based on their comments, we have revised the manuscript. Changes within the manuscript are highlighted as tracked changes. Please see below for a point-by-point response to the reviewers' comments.<br><br>Reviewer #1<br><br>Reviewer Comment:<br>The study measured how somatic variant classification with long-read sequencing (LRS) technology was affected by data quality: tumor sample purity and read depth. ……….., public release of the variable purity and sequencing depth dataset would enable further research into how data quality impacts somatic variant calling.<br><br>Response: We thank Reviewer 1 for the summary and highlighting the impact of our research. We also agree that the variable tumour dataset will enable further research. Therefore, we have deposited the BAM files of these data in the EGA under accession number EGAD00001015628 under study EGAS00001008107. The POD5 files are available on request. For the variable sequencing depth, these data were created from the sequence data which has been deposited in the EGA.<br><br>Reviewer Comment:<br>Strengths<br>1. The impact of the work included 1) informing data quality standards for somatic variant analysis, 2) hypothesizing somatic variants distinct to LRS methods and missed by SRS methods, and 3) a nuanced evaluation of various somatic variant calling tools in different data quality scenarios.<br>2. The analysis was novel compared to previous germline variant LRS benchmarks since, here, the experiments evaluated data quality aspects distinct to cancer datasets: tumor purity and sequencing depth of paired tumor and normal samples.<br>3. Interpretation of experimental results was mostly correct. And, the discussion section accurately summarized key findings regarding the effect of tumor purity and sequencing depth on somatic variant calling.<br><br>Response: We appreciate these positive comments and highlighting the novelty of the work.<br><br>Reviewer Comment:<br>Weakness<br>1. The lack of analysis for overlapping true positives or false positives across methods was a missed opportunity to understand the potential impact of ensemble methods. Albeit, an analysis of overlapping calls is slightly out of scope of this work. Therefore, I do not see overlap analysis as a necessary revision.<br><br>Response: We expect that an ensemble callset derived from ClairS and DeepSomatic to have higher precision as events detected by both methods are less likely to be false positives. Meanwhile, this comes at the cost of lower recall, since some true positive events may only be captured by a single method. To test this, we performed an inspection of SNV that were called by ClairS and DeepSomatic using samples from our tumour purity experiments.<br>Changes to manuscript: The results of this overlap analysis are provided in a new Supplementary table (See Supplementary Tables) and we included new text in Results section 2.2 (Page 6):<br>"The majority of the SNVs were detected by both approaches, with 90% of SNV being detected by ClairS and DeepSomatic for the COLO829 non-diluted sample and 87% for HCC1937 (Supplementary Table S2). The union of the two approaches achieved higher recall at the cost of introducing more FP events."

Powered by Editorial Manager® and ProduXion Manager® from Aries Systems Corporation

	Reviewer Comment:<br>2. The argument for filtering ClairS by a singular variant statistic should be cautioned by the implied decrease in recall when applying filters. From the results figures, it's unclear whether any threshold could be applied for a singular variant statistic (e.g., sequencing depth, mapping quality, or variant allele frequency) without a significant reduction in recall. A safer argument is to filter by a combination of these statistics.<br><br>Response: We agree with the referee regarding the statement of using a combination of statistics to apply filters.<br>Changes to manuscript: We have revised the statement in the Results section 2.2 (Page 6) to improve clarity. The updated text now reads:<br>“The different patterns observed in allele frequency, read depth, and quality score distributions between TP and FP events suggest that integrating these measures could serve as an efficient filter to reduce FP calls.”<br><br>Reviewer Comment:<br>Minor comments<br>Interpretation of SF16 results seems incorrect. The majority of LRS FPs overlap with high/low GC content regions for most methods, not low mappability regions. This argument is mentioned on Pg 9, sec 2.4, paragraph 2, lines 4-5.<br><br>Response: Thank you for the correction.<br>Changes to manuscript: We have revised the text to clarify the interpretation. The corrected sentence now reads:<br>“In support of this, we found that a substantial fraction of the LR-specific SVs are located at genomic regions of extreme GC content (GC%<30 or >55) or within low mappability for short reads”.<br><br>Reviewer Comment:<br>Regarding the Figure 5A analysis, I do not understand how SBS vectors are set up for cosine similarity measurement. Please make it clear how SBS vectors are set-up/aggregated from variants. Also, a baseline reference of the expected cosine similarity at random would make this result more impactful and interpretable.<br><br>Response: SBS vectors refers to the single base substitution (SBS) profile categorised by the base change type (C>A, C>G, C>T, T>A, T>C, and T>G) within a trinucleotide context (the immediate 5’ and 3’ neighbours). This results in a 96-dimensional vector (SBS-96), which is the standard framework for describing mutational signatures in cancer (as described in [1]). These SBS signatures can provide some insight about how tumours develop. For example, in the COLO829 cell line, which is derived from a sun exposed melanoma sample, a high proportion of the SBS somatic mutations are linked to signatures associated with UV exposure (SBS7). Therefore, in Figure 5A, we tested whether the mutational profiles of LR variants classified as FP SNVs resemble those in short-read “gold standard” – and thus more likely to be true events.<br>Cosine similarity is a common approach to compare signatures (eg. [2,3]) and was used as it is insensitive to the absolute scale of counts, the shape of mutational spectrum across 96 channels is what matters. The high cosine similarity (0.79-0.97) we observe indicates that the FP calls recapitulate the same mutational patterns as the gold-standard set, suggesting that many of these calls may indeed reflect real biological signatures rather than random sequencing artefacts. This is because a cosine similarity score >0.85 suggests the sample’s mutational profile strongly matches the reference, 0.6-0.8 represents partial similarity and <0.5 score suggests little resemblance. Our random simulation based on random sampling 96-SBS channels showed a background similarity of approximately 0.35.<br>Changes to manuscript: We agree that Figure 5A is rather a complicated figure and realised the main reason for the complexity is that we missed the method description for these results. We apologise for this omission and have now included a new section in the Methods (Section 4.13) which reads:<br>“4.13 Mutational signatures analysis<br>All SNV somatic mutations were converted into a 96-channel mutational profile (SBS-96 vector) and were subsequently decomposed into contributions of COSMIC reference catalogue (v2.0, GRCh38) using the R package deconstructSigs (v1.8.0) [83]. To evaluate whether LR specific variants, classified as false positives, might represent genuine mutational events, we compared their mutational spectra with the

short-read gold-standard set. Specifically, a cosine similarity was computed between the two profiles."

Reviewer Comment:
The results of supplementary figure (SF) 3 seemed unrealistic to me. Specifically, I am surprised that high/low methylation always gets reversed as a function of purity. Please provide some intuition for why this result is (un)-expected, and double-check this result.

Response: The methylation frequency distribution plot (Supplementary Figure S3B) only shows the CpG sites with differential methylation frequency between tumour and blood lymphocytes cell lines. These sites were identified from EPIC Array data by extracting sites with tumour-specific methylation status where the beta values (methylation frequency) are ≥0.7 in tumour and ≤0.3 in BL, or ≤0.3 in tumour and ≥0.7 in BL. Consequently, in the mixing experiments, highly methylated sites show progressive decreases in methylation frequency, while demethylated sites display increases as the proportion of BL DNA rises. To validate that our cell line mixing recapitulated the expected tumour purity levels, we used both variant allele frequency (VAF) distributions and methylation frequency curves as orthogonal measures.
Changes to manuscript: We have included additional text in the Supplementary Figure 3 legend:
"Only the CpG sites with differential methylation frequency between the 100% tumour and blood lymphocyte cell lines are included in the plot."
We have also amended the results text to clarify the findings:
"The shifted distributions of variant mutation allele frequency (VAF) for SNVs (Supplementary Figure S3A) and methylation frequency at CpG sites that are differentially methylated between the tumour and normal cell line (Supplementary Figure S3B) supports the serial dilutions of tumour DNA by normal cell DNA."
We have also clarified the approach in a revised Methods section (4.10), which now reads:
"The methylation data was also used to confirm the tumour purity levels within the serial diluted samples. Specifically, differentially methylated CpG sites between the tumour and blood lymphocyte cell lines were determined as probes with Beta values ≥0.7 in tumour and ≤0.3 in BL, or ≤0.3 in tumour and ≥0.7 in BL. The distribution of these CpG sites was then assessed in the samples with different tumour purities for COLO829 and HCC1937."

Reviewer Comment:
When reading the paper top-down, it is confusing why the definitions of FP, TP, and FN were switched to LRS variants, overlapping variants and SRS variants in SF7. The analysis of SF7 preceded the evidence of LRS FPs being missed by the SRS standard shown later in Fig 5. Overall, the foreshadowing of LRS FPs being missed calls made me skeptical until there was evidence supporting this hypothesis later in the text.

Response: We hold a premise that long reads can improve variant calling in genomic regions that are difficult for short reads to align, especially for structural variation. For this reason, we emphasised this point in the SV benchmarking and originally referred to FP as LR novel and FN as SR specific events in Supplementary Figure S7 and S10. However, as pointed out by the reviewer, we acknowledge that introducing this terminology before Figure 5 may have caused confusion.
Changes to manuscript: We have now updated Supplementary Figure S7 and S10 to use the standard FP, FN and TP labels for consistency.

Reviewer Comment:
The supplementary figure 8 legend is incorrect. Column and row descriptions do not match the figure. And, what does DP stand for?

Response: Thank you for bringing this error to our attention.
Changes to manuscript: The column and row descriptions in the Supplementary Figure S8 legend have been corrected, and we have clarified that DP refers to the read depth in the tumour sample. The figure legend now reads:
"Supplementary Figure S8. Variant allele frequency (VAF), read depth in tumour (DP) and quality score (QUAL) of FP and TP SNVs calls from ClairS and DeepSomatic in COLO829 and HCC1937 samples with nine sequencing depth combinations. Panels are arranged with COLO829 on the left and HCC1937 on the right, and ClairS on the

Powered by Editorial Manager® and ProduXion Manager® from Aries Systems Corporation

top and DeepSomatic on the bottom. Within each panel, results are further separated by sequencing depth combinations ranging from 15x -15x to 60x - 60x (tumour - normal). FP calls are shown in blue and TP calls in yellow.”

Reviewer Comment:
A direct quantification, such as the difference of mean recalls across methods, is missing between SRS standard and secondary callset.
Response: Thank you for this suggestion. In the Results section, we have reported mean recall and precision values for both SNV and indel calling to the text.
The text now reads:
Changes to manuscript: “In terms of recall, DeepSomatic had a higher mean recall at all tumour purity levels except the sample with 100% tumour purity compared to ClairS for COLO829 (ClairS 81.27%, DeepSomatic 86.39%), however, ClairS had a higher mean recall than DeepSomatic in HCC1937 in samples with lower tumour purity (<50%) (ClairS 81.43%, DeepSomatic 77.02%).”

Reviewer Comment:
The abstract argues that LRS better aligns to "complex" regions (abstract, line 2). The intro discusses how SRS is bad in low complexity regions (introduction, lines 7&8) … later in the intro, LRS is again discussed as effectively "spanning complex genomic regions" (introduction, line 11). I believe the argument attempting to be made is that LRS better aligns to low complexity regions than SRS. Please make this argument consistent and clear.

Response: Thank you for pointing out this inconsistency. Our intended argument is that longer reads provide improved alignment and variant detection in low complexity regions, which usually contain highly repetitive sequences.
Changes to manuscript: We have revised the wording in the abstract and introduction to ensure consistency and clarity. The updated wording now consistently refers to “low complexity regions” or “highly repetitive regions” rather than “complex regions”.

Reviewer Comment:
For analysis of SF10 in section 2.3, the consistently high precision of nanomonsv seems worth mentioning. This is mentioned in the discussion, but I think it should be mentioned in the results section, too.
Changes to manuscript: We have included new text highlighting the consistently high precision of nanomonsv in the Results section (Section 2.3). The revised text reads:
“Nanomonsv consistently demonstrated the highest precision across tumour purities, underscoring the importance of incorporating a reference panel to achieve accurate variant detection.”

Reviewer Comment:
It would be helpful to indicate which stains are telomere and centromere regions in supplementary figure 11-14 keys.

Response: In the chromosome ideograms, centromeres are coloured in red (acen), but there is no specific code for telomeres as they are the natural ends of chromosomes.
Changes to manuscript: We have revised the key in Supplementary Figures S11-14 to include details of the giemsa staining code and have revised the Figure legends accordingly.
In addition, we have expanded the information for the rainfall plot and chromosome diagram band colouring in the Methods section 4.12 to provide further clarification on the rainfall plots and chromosome ideogram tracks. The new text reads:
“The genome-wide distribution of TP, FP and FN SNVs was visualised using rainfall plots which were aligned with chromosome ideograms annotated with cytogenetic banding patterns (gieStain) obtained from the UCSC genome browser”

Reviewer Comment:
"N50 is" -> "N50 was" (results, paragraph 1, last line)
Make capitalization consistent for the sub-figure character labels in supplementary figure 1.
At section 2.2, paragraph 5, line 2 change plural "indels" to singular "indel"
Discussion, p14, para 8, line 7, "on" -> "one"
Conf of interest, P20, l1, "Nanopre" -> "Nanopo

Powered by Editorial Manager® and ProduXion Manager® from Aries Systems Corporation

Response: These have been fixed. Thank you for finding the errors.

Reviewer #2:

Reviewer Comment: This study assesses the accuracy of long-read sequencing with Oxford Nanopore Technology (ONT) for detecting somatic variants in tumor cell line mixtures, offering valuable insights for optimizing cancer genomic analyses, particularly regarding sequencing depth, tumor purity, and comparisons with short-read technologies. Overall, the manuscript is well-written, clearly structured, and presents significant findings in a highly relevant area of research.

Response: We thank Reviewer 2 for these positive comments and recognition of the quality and value of our research.

Reviewer Comment:
However, the code repository for this study (https://github.com/bakeronit/nanopore_celllines_benchmark) is currently unavailable. It is unclear whether the repository is private or has been renamed. Considering that GigaScience strongly emphasizes reproducibility, the inability to access and verify the repository significantly limits this review.

Response: Thank you for bringing this to our attention. We apologize for the inconvenience caused by the repository being private at the time of review. We did upload the whole github repository as a zipped package to GigaDB FTP server with all intermediate data prior to the review stage. The github repository was initially kept private to ensure that the full code and workflow would be presented in its proper scientific context along with the publication of this manuscript. We fully recognize the importance of transparency and reproducibility. To further facilitate reproducibility, the GitHub repository has now been made publicly accessible, with branch rev has been created to reflect all updates and changes made during our revision. This repository can now be accessed at:https://github.com/bakeronit/nanopore_celllines_benchmark. We hope this resolves the concern and demonstrates our commitment to open and reproducible research.

Reviewer Comment:
The manuscript mentions the use of downsampling to synthetically reduce coverage depth, but upsampling-based simulations were not explored. I strongly recommend including synthetic datasets, as they can substantially enhance both experimental design and result validation. For example: PBSIM2 for PacBio and ONT (https://github.com/yukiteruono/pbsim2), NanoSim for ONT (https://github.com/bcgsc/NanoSim). Such simulations offer several advantages: Validation of the reconstruction methodology; Full control over somatic variants (gold standard); Tunable sequencing coverage; Exploration of extreme scenarios; Cross-validation of real data (false positives/negatives); Systematic technology comparisons; Benchmarking against real datasets. Similarly, short-read simulations could be performed using ART (https://www.niehs.nih.gov/research/resources/software/biostatistics/art) or art_modern (https://github.com/YU-Zhejian/art_modern).

Response: We appreciate this suggestion and did spend some time reviewing NanoSim and pbsim2 as they seem like useful approaches. We tried to use nanosim to simulate ONT reads, however, even with 1% of reads sampled, the model training took more than 48 hours with 30 CPUs and 50Gb RAM. Furthermore, we feel that these approaches are not a good fit for our study because they are unable to capture all sequence artefacts and account for rearranged cancer genomes. We deliberately chose to generate sequence data using in vitro mixing of DNA samples. Although we agree that our DNA mix simulations do not cover all extreme scenarios, we believe that as we sequenced each DNA mixture, they reflect the expected performance of the technology over the majority of common strategies.
While upsampling data can be useful for controlled experiments, the premise of generating new ONT data based on profiles learned from a subset of data is less than ideal in our case. Therefore, we elected to sequence samples quite deeply (>60x) then used downsampling of these data to determine whether less data would still allow

Powered by Editorial Manager® and ProduXion Manager® from Aries Systems Corporation

variant detection. Our downsampling approach was a random process of generating less reads from a sequencer, with every read from the real genome from tumour.
A challenge we are trying to address in this study is the lack of “gold standard” sequence variant data. In particular there is a lack of perfect gold standards for tumour samples to validate variant detection pipelines. Therefore, we hope the data generated in this manuscript will be a useful resource to others.
Changes to manuscript: We have included new text in discussion, acknowledge the potential use of read simulators to assess the extreme scenarios for variant calling.
Now reads:
“Previously researchers have utilised the synthetic mixtures of two fully homozygous human cell lines to mock somatic mutation, and apply read simulators such as NanoSim or pbsim2 to upsample data for validation of variant detection. While this is a good strategy to explore variant calling performance under extreme scenarios, differences in variation calling performance were found when using the real tumour cell line, which underscores the heterogeneous nature of cancer samples.”

Reviewer Comment:
Sequencing technology strongly impacts the interpretation of long-read studies, as differences in error profiles, read length, required depth, and compatible tools directly influence sensitivity, precision, and the types of variants that can be reliably detected. This study exclusively reports ONT-based results, and some conclusions may not generalize to other long-read platforms. It would be more informative to explicitly mention the sequencing technology in the abstract and possibly in the article title.

Response: We acknowledge that our analysis and conclusions are most directly applicable to ONT and may not generalize fully to other long-read technologies such as PacBio HiFi.
Changes to manuscript: To make this scope clear to readers, we have revised the abstract to explicitly mention that the results are based on Oxford Nanopore sequencing.

Reviewer Comment:
The use of GRCh38 is suboptimal when compared to CHM13-T2T, which resolves previously unresolved repetitive, telomeric, and centromeric regions-areas that the study itself identifies as relevant to accuracy. Incorporating CHM13-T2T in the current analysis could significantly strengthen the study, and would also make Supplementary Figures 11-14 more informative.

Response: This is an excellent point. In the original manuscript, we had already raised this point in discussion referencing recent publications that demonstrate the value of CHM13-T2T eg.,[4]. However, GRCh38 remains the current most widely adopted reference in cancer genomics research and clinical use, because of its extensive functional and clinical annotations, integration with large-scale resources (e.g. gnomAD, TCGA), and compatibility with the majority of computational approaches. While we appreciate the value of CHM13-T2T assembly, our main goal for this study is to evaluate the LRS in the current most common clinical cancer genomics setting, which is based on GRCh38.
Even so, we have now conducted new analysis using the COLO829 and HCC1937 cell lines, where we evaluate genomic stratification of calls using CHM13-T2T assembly. We fully agree that the CHM13-T2T assembly represents the most complete human genome assembly to date and provides advantages over GRCh38 for variant calling, particularly in resolving repetitive, telomeric and centromeric regions. This is reflected in what we see in the reduced overall sequence divergence between aligned reads and the reference, as well as in the higher mapping rates observed when aligning to CHM13-T2T compared with GRCh38.
Changes to manuscript: We have included a new Supplementary Table S1 (See Supplementary Tables)

Meanwhile, we refer this supplementary table in the results section and provide rationale for the reason to use GRCh38 in our analysis.
“We assessed impact of alignment using different human reference assemblies, CHM13-T2T and GRCh38. Slight improvements were observed using the CHM13-T2T reference with lower sequence divergence between reads and reference sequences and higher mapping rates (Supplementary Table S1). However, in our study we used

Powered by Editorial Manager® and ProduXion Manager® from Aries Systems Corporation

	the GRCh38 assembly due to the comprehensive annotations and maximised compatibility with current tools and our short-read gold standard."<br><br>Reviewer Comment:<br>Including metrics that better capture imbalanced variant detection performance, such as AUROC and AUPRC, would provide a more comprehensive assessment of the methods.<br><br>Response: Great suggestion.<br>Changes to manuscript: We have incorporated AUPRC curves for indel and SV calling in the supplementary figure S4 alongside the AUPRC plot for SNV detection showing the trade-off between precision and recall across tumour purity.<br><br>Reviewer Comment:<br>Minor Comments<br>In the sentence: "Two approaches were used for somatic SNVs and indels detection from long-read data. ClairS [32] and DeepSomatic [31]." - consider replacing "." with a ":".<br>Changes to manuscript: We have changed the paper as suggested. This reads better after replacing "." with ":".<br><br>Reference<br>1. Alexandrov LB, Kim J, Haradhvala NJ, Huang MN, Tian Ng AW, Wu Y, et al.. The repertoire of mutational signatures in human cancer. Nature. Springer Science and Business Media LLC; 578:94–1012020;<br>2. Medo M, Ng CKY, Medová M. A comprehensive comparison of tools for fitting mutational signatures. Nat Commun. Springer Science and Business Media LLC; 15:94672024;<br>3. Jiang N, Wu Y, Rozen SG. Benchmarking 13 tools for mutational signature attribution, including a new and improved algorithm. Brief Bioinform. Oxford University Press (OUP); 26:bbaf0422024;<br>4. Paulin LF, Fan J, O'Neill K, Pleasance E, Porter VL, Jones SJM, et al.. Closing the gaps, and improving somatic structural variant analysis and benchmarking using CHM13-T2T. Genome Res. Cold Spring Harbor Laboratory; 35:621–312025;
**Additional Information:**	
**Question**	**Response**
Are you submitting this manuscript to a special series or article collection?	No
**Experimental design and statistics**<br><br>Full details of the experimental design and statistical methods used should be given in the Methods section, as detailed in our Minimum Standards Reporting Checklist. Information essential to interpreting the data presented should be made available in the figure legends.<br><br>Have you included all the information requested in your manuscript?	Yes
**Resources**	Yes

Powered by Editorial Manager® and ProduXion Manager® from Aries Systems Corporation

A description of all resources used, including antibodies, cell lines, animals and software tools, with enough information to allow them to be uniquely identified, should be included in the Methods section. Authors are strongly encouraged to cite Research Resource Identifiers (RRIDs) for antibodies, model organisms and tools, where possible.<br><br>Have you included the information requested as detailed in our Minimum Standards Reporting Checklist?	
**Availability of data and materials**<br><br>All datasets and code on which the conclusions of the paper rely must be either included in your submission or deposited in publicly available repositories (where available and ethically appropriate), referencing such data using a unique identifier in the references and in the "Availability of Data and Materials" section of your manuscript.<br><br>Have you have met the above requirement as detailed in our Minimum Standards Reporting Checklist?	Yes
GigaScience has policies and guidelines in place for the use of generative AI-writing tools such as ChatGPT. If you have used such writing tools to assist with writing the manuscript this must be declared and cited in the text. Authors should not list AI-writing tools and other AI-assisted technologies as an author or co-author and should acknowledge that they are fully responsible for text generated or refined by AI-writing tools.<p><br><br>A summary of use (particularly in the introduction or among methods) needs to be included at the end of the paper, and the outputs should also be included as a supplementary file hosted in GigaDB or other open repositories. Please read our guidelines for more information. <p>	No

By submitting to GigaScience, you are aware of the journal's AI-writing tools policy, and if you have declared use of such tools below, you have acknowledged this where appropriate in your manuscript and have made a summary of use and outputs available. <p> <b>AI-assisted writing tools have been used in the preparation of this manuscript?	

Powered by Editorial Manager® and ProduXion Manager® from Aries Systems Corporation

Manuscript_Marked
Click here to access/download;Manuscript;Zhang et al main_revision_Marked.docx

Cancer genome standards for long-read sequencing using cancer cell line mixtures

Jia Zhang[1,2], Ho Yi~~yi~~ Wong[1,2], Lingchen Liu[1,2], Lambros T. Koufariotis[1], Scott Wood[1], Nadine Fitzpatrick[1], Jenny Quiatchon[1], Paul Collins[1], John V. Pearson[1,~~2~~], Nicola Waddell[1,2]

[1]QIMR Berghofer Medical Research Institute, Brisbane, QLD, Australia

[2] School of Biomedical Sciences, ~~Faculty of Medicine~~, The University of Queensland, Brisbane, Australia.

Commented [NW1]: I think this has changed now and we are now:
School of Biomedical Sciences,

Abstract (199 words)

Long-read sequencing (LRS) improves genome alignment and facilitates resolving variants in ~~complex~~ genomic regions of low complexity, making it a promising approach for cancer variant detection and biomarker discovery. Here, we evaluate the performance of LRS using Oxford Nanopore sequencing to detect somatic variants across different tumour purities and sequencing depths by comparing to somatic variants from short-read sequencing. We generated experimental mixtures of cancer cell lines and matched normal cell lines to simulate 10 tumour purities (ranging from 0 to 100% tumour content). This resulted in 22 samples which were sequenced using whole genome LRS to a targeted depth of 60x and somatic variants identified. We down-sampled data to explore the optimal read depth for somatic variant detection. Our results show long-read variant calling tools achieve recall rates comparable to short-read gold standards. While tumour sequencing depth of 30x to 60x is generally sufficient for detecting common variants, particularly structural variants, sequencing the matched normal sample at adequate depth is crucial for accuracy. Notably, we found variants unique to LRS that may represent real and previously undetected events. This study highlights key factors for optimising cancer genome sequencing with LRS and provides a comprehensive dataset of cell line mixtures for future research and tool development.

Keywords

Long read sequencing, Oxford Nanopore Technologies, somatic mutation, tumour purity, sequencing depth

1. Introduction

Cancer is a complex and multifaceted disease characterised by diverse genetic and epigenetic alterations, such as single nucleotide variations (SNVs), structural variations (SVs), and changes in base methylation [1–3]. These genomic changes drive abnormal cellular proliferation and carcinogenesis [4], and represent targets for treatment [5] making the detection of mutations in tumour cells crucial for cancer research and precision therapy [6,7]. While high-throughput short-read sequencing (SRS) has been widely used to identify somatic mutations across various cancer types, it often fails to detect variations in low-complexity, highly repetitive, or GC-biased regions due to limited mappability [8–10]. Long-read sequencing (LRS) technologies, such as those developed by Pacific Biosciences (PacBio) [11] and Oxford Nanopore Technologies (ONT) [12], offer extended read lengths that can span highly repetitive ~~complex~~ genomic regions effectively [13]. Initially used to enhance reference genome assembly and to resolve haplotypes [14,15], LRS is increasingly applied in cancer genomics due to its ability to identify novel variations in previously unresolved regions of tumour genomes [16–20]. Recent studies using high-throughput platforms like the Nanopore PromethION have enabled rapid and comprehensive profiling of various advanced cancer cohorts [20] and central nervous system tumours [21,22], demonstrating significant potential for clinical applications. However, the use of LRS in cancer has largely been restricted to a small number of studies or the analysis of cell lines, which underscores the importance of benchmarking these technologies to optimise their clinical and research use.

The evolution of LRS technologies has led to a proliferation of approaches for analysing the resulting data [23], including basecalling, alignment and variant calling. LRS requires computational methods that are distinct from SRS methods due to its longer read length and

unique error profiles [24,25]. Benchmarking studies have evaluated various LRS tools to analyse cancer genome data, including aligners, somatic structural variation detection and methylation detection methods [26–28]. However, many of these evaluations relied on *in-silico* data or sequence data from cell-lines [29,30], meaning they did not capture complexities of tumour tissue samples such as tumour purity. Notably, some benchmarking efforts are published alongside the development of new tools, which can introduce bias as optimal parameters may not be used for each tool [31–34]. Therefore, independent benchmarking is valuable with the continuously emerging new tools and frequent version updates for existing tools [35].

Tumour purity and sequencing depth have a significant impact on the data quality and interpretation in tumour genomics studies [36–38]. Tumour purity refers to the proportion of cancer cells in a sample and it can affect the signal-to-noise ratio, particularly for detecting somatic alterations (mutations specific to the cancer cells in a sample). Low tumour purity can mask somatic mutations by lowering the somatic variant allele frequency, potentially leading to false negatives [39]. Sequencing depth determines the sensitivity for identifying low-frequency variants as well as the ability to distinguish signals from germline cells, therefore, sequencing depth is particularly crucial in heterogeneous tumours [35] where low tumour purity and sub clonal mutations are prevalent. Together, tumour purity and read depth influence the ability to detect somatic mutations, analyse clonal evolution, and understand tumour heterogeneity. Inadequate tumour purity or sequencing depth can result in the incorrect detection of clinically relevant alterations, which will negatively impact genomic discovery for cancer research and precision medicine [38,40]. The implications of tumour purity and sequencing depth for LRS methods have not been explored.

In this study, we created cancer reference samples from two pairs of matched tumour-normal cell lines, to measure the impact of tumour purity (0 to 100%) and sequence depth on somatic mutation detection. We sequenced the samples to an average read depth of >60x using Oxford Nanopore long-read sequencing. We compared the somatic variant calls from

different experimental settings against our SRS gold standard to determine optimal sequencing strategies. The results establish guidelines for expected data quality based on differing tumour purity levels and sequencing depths, providing valuable insights for optimising experimental design in tumour sequencing studies. Furthermore, the generated dataset serves as a valuable resource for the development and validation of bioinformatic tools in this rapidly evolving field.

2. Results

2.1 Study design and overview

We generated two tumour-normal paired cancer samples to simulate different tumour purities and sequence read depths to evaluate the ability of LRS to identify somatic mutations (**Figure 1**). We cultured two cell lines representing distinct tumour types (melanoma, COLO829 and breast cancer, HCC1937) and a matched non-tumour cell line for each. DNA was extracted from each cell line and matched tumour and non-tumour derived DNA were serially mixed to simulate 10 tumour purity levels at 10% increments (0 to 100%). This resulted in 10 tumour mixtures and one matched blood lymphocyte (BL, as a germline/normal control) sample per cell line pair. A sequencing library was made for each sample and sequenced with the PromethION from Oxford Nanopore technologies using two flow cells per library (R10.4.1). Basecalling was performed using Dorado (v0.5.1) with the super accuracy (SUP) model. The mean and median read quality scores were 21.8 and 23. The average yield per flow cell was 126 Gb, and the read length N50 was~~is~~ 10.4 Kb (**Supplementary Figure S1A**).

Two experiments were designed to assess the impact of common variables on somatic mutation calling in cancer samples: tumour purity and sequence read depth. To assess the impact of tumour purity, the 10 tumour cell mixtures and BL germline samples were sequenced to a mean depth of 70.61x for COLO829 (range 58.29x to 79.13x) and 61.57x for HCC1937 (range 53.42x to 71.14x) (**Supplementary Figure S1B),** resulting in >95% of the

genome being covered by in at least 1 read (**Supplementary Figure S1C**). The N50 (median aligned read length) for each library ranged from 9.4 to 10.4 kb (**Supplementary Figure S1d**). We assessed impact of alignment using different human reference assemblies, CHM13-T2T and GRCh38. Slight improvements were observed using the CHM13-T2T reference with lower sequence divergence between reads and reference sequences and higher mapping rates (**Supplementary Table S1**). However, in our study we used the GRCh38 assembly due to the comprehensive annotations and maximised compatibility with current tools and our short-read gold standard. To evaluate the performance of mutation detection under different sequencing read depths we randomly subsampled sequence reads from four tumour mixtures (100%, 80%, 60% and 40%) per tumour type (COLO829 and HCC1937) and the matched BL sequence data to create tumour and germline BAM files with 60x, 45x, 30x, and 15x sequencing read depths. This resulted in nine combinations of sequencing depths.

In both experiments, we compared multiple approaches to call somatic single nucleotide variants (SNVs), small insertions and deletions (indels) and structural variants (SVs). To assess the accuracy of the mutations detected with the LRS, we created a "gold standard" call from short-read data by sequencing DNA from the two tumour and matched non-tumour cell lines using three short-read replicate libraries. Sequencing resulted in a minimum read depth of 34x in BL and 67x in tumour samples, and we identified 40,422 SNVs, 416 indels, and 57 SVs in the "gold standard" short-read somatic calls for COLO829 and 48,982 SNVs, 3,236 indels and 537 SVs for HCC1937 (**Supplementary Figure S2A-F**).

2.2 Tumour purity impacts somatic mutation detection

Patient tumour tissues have different tumour purities, therefore, we created 10 tumour mixtures for each cell line to explore how tumour purity impacts somatic mutation detection from LRS. Two approaches were used for somatic SNVs and indels detection from long-read data. ClairS [32] and DeepSomatic [31]. The shifted distributions of variant mutation allele

frequency (VAF) for SNVs (**Supplementary Figure S3A**) and methylation frequency at CpG sites that are differentially methylated between the ~~in~~ tumour and normal cell line ~~specific methylation status~~ (**Supplementary Figure S3B**) ~~confirmed the expected gradient of descending AF and methylation frequency with decreasing tumour purity.~~ ~~reflect~~supports the serial dilutions of tumour DNA by normal cell DNA.

Overall, the number of somatic mutations detected by ClairS and DeepSomatic decreased with decreasing tumour purity, resulting in a lower recall (**Figure 2A**). Nevertheless, even at low tumour purity, the precision remained high (>79.5~~1~~%). DeepSomatic had a higher precision than ClairS for COLO829 (ClairS range from 82~~1~~.2~~4~~% to 92.8~~5~~%, DeepSomatic range 95.4% to 98.4%) and HCC1937 (ClairS range 79.5~~1~~% to 86~~5~~.1~~9~~%, DeepSomatic range 84.5% to 93.5%) (**Figure 2A** and **Supplementary Figure S4**). In terms of recall, DeepSomatic had a higher mean recall at all tumour purity levels except the sample with 100% tumour purity compared to ClairS for COLO829 (~~mean:~~ ClairS 81.27%, DeepSomatic 86.39%), however, ClairS had a higher mean recall than DeepSomatic in HCC1937 in samples with lower tumour purity (<50%) (ClairS 81.43%, DeepSomatic 77.02%). The majority of the SNVs were detected by both approaches, with 90% of SNV being detected by ClairS and DeepSomatic for the COLO829 non-diluted sample and 87% for HCC1937 ~~and the intersecting call set showed a modest increase in precision while a slight reduction in recall~~ (**Supplementary Table S2~~1~~**). The union of the two approaches achieved higher recall at the cost of introducing more FP events.

The variant allele frequency of true positive (TP, present in “gold standard”) calls decreased with decreasing tumour purity for both COLO829 and HCC1937 (**Figure 2B**) as expected. In contrast, the false positive (FP, absent from “gold standard”) calls for COLO829 do not show ~~an~~a decrease in AF, with FP having a greater range of AF in both COLO829 and HCC1937 at higher tumour purities (**Figure 2B**). Interestingly, for ClairS, the read depth for FP calls was consistently lower than for TP calls (**Supplementary Figure S5A**). This was not the case for DeepSomatic, suggesting that DeepSomatic FP variants might be TP variants

missed by SRS meanwhile read depth could be used as a filter to remove real FP events in ClairS. The quality scores were lower for FP events compared to TP for both callers (**Supplementary Figure S5B**). The different patterns observed in allele frequency, read depth and quality score distributions between TP and FP events~~,~~ suggest~~ing~~ that ~~quality score could be used~~integrating these measures could serve as an efficient~~a~~ filter to reduce~~remove~~ ~~potential~~ FP calls.

The average recall for indels (ClairS 71.~~1~~62% for COLO829, 80.28% for HCC1937; DeepSomatic 57.85~~42~~% for COLO829, 62.66~~55~~% for HCC1937) (**Figure 2C**) was lower than SNV ~~(ClairS 810.279% for COLO829, 81.431% for HCC1937; DeepSomatic 865.3976% for COLO829, 776.0288% for HCC1937)~~ (**Figure 2A**). ClairS had a higher recall for indels at all tumour purity levels than DeepSomatic. Similar to SNVs detection, the number of somatic indels detected by ClairS and DeepSomatic decreased with decreasing tumour purity, resulting in a lower recall (**Figure 2C**). The average precision was significantly lower for indels (ClairS: 24.6~~47~~% for COLO829, 42.39~~3~~% for HCC1937; DeepSomatic: 68.55~~4~~% for COLO829, 64.27~~6~~% for HCC1937) than for SNVs (ClairS: 90.57~~26~~% for COLO829, 83.61~~35~~% for HCC1937; DeepSomatic: 96.~~2~~87% for COLO829, 89.29~~7~~% for HCC1937). The ClairS approach consistently had a lower precision in all tumour purities compared to DeepSomatic for both cell lines (**Figure 2C**). There was no difference between the detection of insertion and deletion events (**Figure 2C**).

Similar to the SNVs calls, the mutation allele frequency of true positive (TP, present in “gold standard”) indel~~s~~ calls decreased with decreasing tumour purity for both COLO829 and HCC1937 (**Figure 2D**). The AF of TP indels was higher than the FP calls for ClairS but not DeepSomatic, suggesting that AF could be used as a filter to remove FP indel events in ClairS. The read depth for FP and TP calls was similar for indel events in ClairS and DeepSomatic (**Supplementary Figure S6A**), suggesting that read depth is not useful for determining TP events. The quality scores were lower for FP events compared to TP for

both callers (**Supplementary Figure S6B**), suggesting that quality could be used as a filter to remove potential FP calls.

We compared the SV calls under different purities using four SV detection tools in two cell lines. Most SV calls in the short-read “gold standard” are identified with long-read SV callers, however with 3 out of 57 in COLO829 and 87 out of 573 HCC1937 short-read “gold standard” SVs were not detected by any LR tool in all tumour purity levels (**Figure 3**), suggesting these events may be missed by LRS or false positive calls in the short-read data. Samples with higher purity usually generate more SV calls (**Supplementary Figure S7**), with a decreasing SV count as purity decreases, particularly in HCC1937, where there are more SV events (573, compared to 57 in COLO829). The Severus tool displays the best recall rates (36.84% to 84.21% in COLO829 and 26.18% to 78.53% in HCC1937) at all tumour purity levels for both cell lines. Even at 20% tumour purity, the recall for Severus is 63.16% for COLO829 and 50.79% for HCC1937. In contrast, the recall for other tools at 20% tumour purity for COLO829 and HCC1937, respectively, was 42.11% and 36.47% for Delly, 40.35% and 35.25% for nanomonsv, and 28.07% and 20.59% for SAVANA. The recall rate for SAVANA was comparable to nanomonsv in higher purity samples (>60%), however, SAVANA did not perform as well in lower purity samples (**Supplementary Figure S7**).

2.3 Sequencing depth affects somatic mutation calls

We sub-sampled tumour and germline samples to simulate different read depths (60x, 45x, 30x, and 15x) and then compared the somatic mutation detection under four purities (100%, 80%, 60%, and 40%). This resulted in nine scenarios of tumour-germline sequencing depths under 4 tumour purities (**Figure 1**).

Lower read depth in the germline sample is expected to impact somatic SNVs detection. For ClairS, this resulted in an increase in recall and a decrease in precision, whereas for DeepSomatic, it resulted in a lower recall and a higher precision. When the germline samples were sequenced at 15x sequencing depth, the performance of these approaches

was severely impacted. The precision dropped below 65% in both cell lines for ClairS, and the recall also dropped below 65% in HCC1937 for DeepSomatic (**Figure 4A**). Furthermore, we observed the average allele frequency of FP SNVs when the normal sample at 15x depth higher than other read depth combinations (**Supplementary Figure S8**). Similarly, in indel calling, lower sequencing depth in normal samples leads to decreased precision but increased recall for ClairS except when normal samples had only 15x read depth, whereas DeepSomatic results in reduced recall but improved precision (**Figure 4B**). Interestingly, ClairS demonstrates robustness to the impact in the HCC1937 cell line, while DeepSomatic shows greater robustness in COLO829. Similar results were observed for samples with lower tumour purity (**Supplementary Figure S9**).

Sequencing depth in both tumour and normal samples had a smaller impact on SVs calling than SNVs and indels calling, particularly for nanomonsv and Severus (**Supplementary Figure S10**). Nanomonsv consistently demonstrated the highest precision in four tools across tumour purities, underscoring the importance of incorporating a reference panel to achieve accurate ~~in for somatic~~ variant detection. Meanwhile, SAVANA is more sensitive not only to tumour purity but also to sequencing depth. In terms of recall, Delly outperformed SAVANA in recall when germline samples were sequenced at 15x, although its recall remained lower than that of nanomonsv and Severus. Severus exhibited the highest recall among the four tools across all depth combinations and detected more LR-specific (FP) SVs than nanomonsv, particularly in scenarios with lower depth in the normal sample. While the LR-specific SVs may represent novel SVs, they could also be read false positives.

2.4 False positives detected by LRS might be true

Despite the high recall rate in variant detection using LRS compared with SRS, we found that many LR-specific false positive calls might be real. The proportions of single substitution signatures (SBS) catalogues extracted in the false positives had high cosine similarity to our short-read gold standard with 0.79 and 0.89 for ClairS, 0.96 and 0.97 for DeepSomatic in

COLO829 and HCC1937 (**Figure 5A**). Notably, ClairS detected more T to C (A to G) type mutations with a context AA existing in FP positive calls for both cell lines. We also found that false positive SNVs from ClairS tend to cluster along the centromere and telomere regions (**Supplementary Figure S11 and Supplementary Figure S12**) along chromosomes for both cell lines compared with DeepSomatic (**Supplementary Figure S13 and Supplementary Figure S14**). This suggests that DeepSomatic has more stringent variant calling at difficult genome regions. The genome stratification of false positive SNVs shows that ClairS calls more FPs in tandem repeats and low mappability regions while DeepSomatic calls more FPs in areas of high or low GC%. Meanwhile, the genome stratification of false positive indels displays ~~the~~ a similar pattern between the two tools with a higher proportion of FPs overlapped with tandem repeats and genomic regions with GC percentages <30% or > 55% (**Supplementary Figure S15**).

The high-confidence LR SV calls presented in at least two out of four tools also contain many LR-specific SVs. While these SVs may appear as false positives, we expected some of these SVs to be real due to the technical advantage of long-read over short-read. In support of this ~~particular~~, we found that a substantial fraction of the~~ose~~ LR-specific SVs are located at genomic regions of extreme GC content (GC% <30 or >55) ~~and~~or within low mappability for short reads (**Supplementary Figure S16**). Furthermore, these events are mostly deletions, insertions and duplications with shorter lengths (<10 kb) (**Figure 5B, Figure 5C**), which means they can be sequenced by a single long read with high confidence. Meanwhile, SRS might miss these smaller SVs (<10 kb) due to the restriction of the length of reads and the uncertainty of the pair-end library sizes.

2.5 Germline leakage is more related to the sequencing depth but not tumour purity

Germline leakage refers to germline mutations being misclassified as somatic calls in the variant detection pipeline [41]. We estimate the germline leakage rate by quantifying somatic calls with a maximum population frequency above 0.1 in gnomAD (version 4.1) [42]. While

this threshold may not capture all germline variants, we used these values to compare the germline leakage across tumour purity and sequencing depth. We found that the germline leakage rates remain low across tumour purity and start to increase with tumour purity less than 30% for ClairS while decreasing for DeepSomatic (**Supplementary Figure 17A**). Notably, we found that the leakage spiked when the sequencing depth in the germline was 15x, suggesting enough depth in germline samples is crucial for avoiding germline variation contamination in somatic mutation detection (**Supplementary Figure 17B**).

2.6 Benchmarking SV using publicly available gold-standard

Using short-read data to build the SV gold standard is a challenge due to technical limitations of SRS to detect the full set of SVs in the genome. Previously, a COLO829 SV truth set SV including 68 SVs was thoroughly characterised with multiple sequencing platforms, including short-read and long-read technologies [43]. We benchmarked our long-read SVs to this truth set to evaluate how many SVs that were missed by our SR gold standard can be detected by LRS. The recall rates are slightly lower than the results when benchmarking with our SR gold standard (**Supplementary Figure S18**). This suggests our "gold standard" captured most SVs, however, differences exist because our "gold standard" was constructed using single-platform sequencing data and also genetic changes may accumulate in cell line passaging causing cells from different organisations to increasingly differ across time.

3. Discussion

In this study, we sequenced series of *in vitro* cell line mixtures with different tumour purity levels from two cancer types and compared the performance of long-read somatic variant detection tools, including SNVs, indels, and SVs (≥50bp). Additionally, our *in silico* sequencing depth down-sampling allowed us to evaluate the impact of sequencing read depths of tumour and normal samples on variant detection. Our results provide a practical guide for cancer genomic sequencing of clinical samples, and the dataset of sequenced

mixtures could be valuable for benchmarking the robustness of variant calling tools under different tumour purity settings.

Previous studies have benchmarked long-read sequencing for variant calling in a range of genomic contexts, but many focused on germline-specific variants or prioritised specific genomic features such as SVs [27], CpG methylation [44], and mRNA [45], some with the impact of tumour purity addressed by the *in silico* mixing dataset [29]. However, there remains a critical need to benchmark LRS for variant calling in the context of tumour genomics. Unlike germline variants, somatic mutations in tumours exhibit substantial heterogeneity, both within and across cancer types, posing unique challenges for accurate detection [46]. Through experimental mixing DNA of tumour and normal cell lines and *in silico* down-sampling data, we showed that LRS tools can detect >50% of the variants of all kinds from a short-read "gold standard" at tumour purities as low as 20% and for 30x to 60x sequencing depth in tumour. Importantly, our data show that having enough depth in germline sample (>15x) is critical for recall and precision of somatic mutations. These findings can guide parameters for clinical sequencing protocols.

As expected, tumour purity significantly affects somatic mutation detection, with lower purity reducing sensitivity across SNVs, indels, and SVs. This highlights the challenge of identifying rare somatic alleles in samples with significant stromal cell content. However, even at low tumour purity (e.g., 20%), LRS, especially with Severus for SVs, maintains a reasonable recall rate, demonstrating its utility in low-cellularity samples. Although Nanopore sequencing has lower base qualities (Q20–Q30) than the current SRS (Q30–Q40), deep-learning-based SNV callers like ClairS and DeepSomatic effectively mitigate sequencing errors. ClairS combines pileup and full-alignment models using Bi-GRU and ResNet architectures to classify variants as germline, somatic, or artifacts [32]. DeepSomatic, using a CNN-based model, processes alignment pileups as images [47]. These architectural differences likely drive performance differences. DeepSomatic's higher precision for SNVs and indels suggests a more conservative, error-resistant approach, possibly due to its

training data and algorithm design. While ClairS achieves comparable SNV recall and better indel recall, it has lower precision, suggesting it requires stringent downstream filtering of variants based on biological context. Notably, ClairS detects variants in centromeric and telomeric regions, indicating its potential to identify mutations in genomic regions traditionally inaccessible to short reads. These results suggest that despite lower base qualities, LRS is able to accurately call somatic mutations even in samples with low tumour content.

Among the four long-read SV detection tools evaluated, Severus consistently outperformed Delly, nanomonsv, and SAVANA across varying tumour purities and sequencing depths. Severus maintained high recall even at low tumour purity and sequencing depth (15×), suggesting its ability to detect tumour-specific signals with minimal interference from germline contamination. SAVANA's performance declined at lower tumour purities and showed high sensitivity to reduced sequencing depth, potentially limiting its applicability in clinical settings. However, its recent integration of tumour purity, ploidy, and copy number alteration (CNA) inferences may address these problems [34]. Nanomonsv performed similarly to SAVANA but remained more robust at lower tumour purities and sequencing depths. Its use of an internal human germline SV reference panel makes it more conservative in SV detection, which aligns with previous findings [27,48].

LRS inherently offers advantages for SV detection over short-read approaches. Given that SNVs and small indels detection show comparable performance [49] between long- and short-read technologies [50], a key question is whether LRS enhances SVs discovery by detecting variants missed by SRS. While previous studies suggested that most large SVs in tumour genomes are detectable by short reads, our findings indicate that long reads uniquely capture numerous short-to-intermediate SVs (<10 kb), which were underrepresented in earlier analyses [51]. This further underscores the importance of LRS in tumour genomics, particularly for identifying novel SV markers beyond the resolution of short-read methods.

To enable future cancer genomics studies and translate LRS into the clinic, we need to know what read depth is required to reliably detect somatic mutations. Our sequencing depth down-sampling experiment suggests that balancing coverage between tumour and normal samples can improve somatic variant detection potentially by minimizing germline contamination. Furthermore, sequencing depths of 15x or less for the germline sample significantly harms somatic variant calling and should be avoid. In our study, we sequenced both tumour and normal samples using two flow cells aim for a read depth of 60x. While we obtain data of ~30x genomic sequencing coverage per flow cell, a study has shown that a single Nanopore Promethion flow cell can generate >33× human genome coverage with ultra-long reads (N50 >35 kb) with optimized DNA processing and library preparation [52]. Assuming that sequencing with a shorter (10Kb) read length N50 produces higher yields per flow cell [49], in future we can reduce sequencing costs by using only one flowcell each for tumour and normal.

Although long reads allow improved mapping in ~~complex~~ genomic regions of low complexity, the alignment accuracy remains imperfect [53]. Some suggest the reason to be low read accuracy, however, the main factor could be the genome assembly [54]. We used the GRCh38 assembly in this study as it provides more comprehensive clinical annotations for the downstream analyses, however, GRCh38 remains incomplete with unresolved gaps and repetitive sequences of unknown size. Recent studies suggest that using the CHM13-T2T [14] assembly results in better genetic variant detection from more precision alignment, especially in highly repetitive regions such as centromeres and telomeres [55,56]. Future studies exploring the impact of reference assemblies on variant calling performance may provide valuable insights into cancer genome sequencing.

The “gold standard” used in this study was constructed with the SRS with all the limitations that implies. Previously researchers~~Some~~ have utilised the synthetic mixtures of two fully homozygous human cell lines to mock somatic mutations [57], and apply read simulators such as NanoSim [58] or pbsim2 [59] to upsample~~ing~~ data for validation of~~e~~ variant

Field Code Changed

detection in control. While this is a good strategy to explore variant calling performance under extreme scenarioshowever, differences in variation calling performance were found when using the real tumour cell line [33], which underscores the heterogeneous nature of cancer samples [60][58]. A major output of our study is the creation of reference data (with a range of tumour purities) that can be used by research community. To this end, we deliberately selected two cancer types, one with a high SNV burden, the other with a high SV burden. Establishing references for other key features of cancer genomes, such as subclone mutations, tandem repeats, and complex SVs, will largely facilitate the development of better computational tools [61,62][59,60].

This study explored the optimal experimental design for cancer genome sequencing using LRS through creation of a tumour purity mixtures. Many LRS analysis tools are still under active development, and while we are far from a definitive "best practice" for cancer variant calling using LRS, we have investigated key aspects key and highlighted directions for improvement. Importantly, we provide a publicly available dataset of LRS cancer cell line mixtures, offering a valuable resource for future tool development and benchmarking in this fast-evolving field.

4. Materials and Methods

4.1 Ethical statement

This work involves the use of human cell lines, which were approved by the QIMR Berghofer Human Research Ethical Committee (P3577 and P3527).

4.2 Cell culture, DNA extraction and mixing

COLO829 (RRID:CVCL_1137), COLO829_BL (RRID:CVCL_1999), HCC1937 (RRID: CVCL_0290) and HCC1937_BL (RRID:CVCL_3281) were maintained in RPMI +10% FBS. All cell lines were tested for mycoplasma and STR profiling. Cells were harvested between

passages 8 and 10. Cells were dissociated using 0.25% Trypsin at 37oC for 5 mins and spun at 1200 rpm for 5 mins. Cells were washed once with PBS and kept at -80oC until DNA extraction. DNA was extracted using AllPrep DNA/RNA Mini Kit (802024, Qiagen) according to the manufacturer's protocols and eluted in TE buffer. To prepare samples with various tumour purities, both tumour and normal DNA were diluted to 50 ng/uL using the TE buffer. The tumour and its corresponding normal DNA were mixed in specific ratios to create 10 samples that represent tumour purities ranging from 0 to 100% with increments of 10%.

4.3 Library preparation and Oxford nanopore sequencing with Promethion

DNA quantity and quality were assessed using an Implen NanoPhotometer, then run on a TapeStation (Agilent) to evaluate fragment size. High molecular weight DNA was sheared to 10-12 Kb using Covaris g-tubes. Approximately 1 ug of sheared genomic DNA was used to make libraries using the Oxford Nanopore Technologies (ONT) ligation sequencing kit (SQK-LSK114), according to the manufacturer's instructions, except that ligation was extended to 30 minutes. Each library was loaded at approximately 10 fmol on two separate R10.4.1 PromethION flow cells and sequenced for 72 hours on a PromethION P24 sequencer within QIMR Berghofer scientific services.

4.4 Basecalling, methylation calling and alignment

The raw PODpod5 files from the sequencer were basecalled using Dorado (v0.5.1) [63][61] with the super accuracy (SUP) model for the R10 flow cell (dna_r10.4.1_e8.2_400bps_sup@v4.3.0). The 5mC and 5hmC methylation were also detected using dorado with the matched remora model (dna_r10.4.1_e8.2_400bps_sup@v4.3.0_5mCG_5hmCG@v1). SAMtools (v1.17) [64][62] was used to convert the unaligned basecalled BAM file from basecalling to FASTQfastq format and filter out reads with average quality (QS) <10 before alignment, and minimap2 (v2.26) [65][63] was used to align reads to the GRCh38 genome assembly (-y –MD -ax map-

ont). The aligned bam files were sorted and indexed using SAMtools. The percentage of 5mC methylated reads at CpG sites was calculated using modkit (v2.4.0) pileup based on the MM and ML tags of reads from both strands in the bam files (--combine-strands --cpg).

4.5 Read depth simulation

To evaluate the performance of variant calling under sequencing strategies, we used Sambamba (v1.0.1) [66][64] to downsample data from the tumour and the matched B lymphoblast bam files with 60x, 45x, 30x, and 15x read depths in samples with 100%, 80%, 60% and 40% tumour purities. Reads were randomly sampled from the aligned BAM files at the proportion based on the expected and original depth. If a BAM file did not reach a 60x read depth, we used the original BAM file (53.42x for the library of HCC1937 cell line at 40% tumour purity). In total, we generated nine combinations of sequencing depths with either higher read depths in tumour samples (60x-45x, 60x-30x, 45x-30x, 45x-15x, 30x-15x) or equal read depths in both tumour and normal (60x-60x, 45x-45x, 30x-30x, 15x-15x). The depth of subsampled BAM files was confirmed with mosdepth (v0.2.9) [67][65] to check the potential effect of varied read lengths.

4.6 Somatic SNV, indel and SV detection

We used ClairS (v0.1.7) and DeepSomatic (v1.6.0) for SNV and short indel detection in tumour cells. These tools were selected as they are both actively updated and designed to call somatic SNV and indels using a pair of tumour and normal bam files to predict tumour-specific variants using pre-trained deep-learning models. For ClairS, we used the ont_r10_dorado_sup_5khz model for somatic SNV detection and the r1041_e82_400bps_sup_v430 model from rerio for Clair3 (https://github.com/nanoporetech/rerio/tree/master/clair3_models). For DeepSomatic SNV calling, we used default parameters with an internal ONT model provided by Google Health.

The final results in VCF were filtered for somatic SNVs with GT=”1/1” and FILTER=”PASS” using BCFtools (v1.19) [68]~~[66]~~ as described in the DeepSomatic document.

We used four tools (Delly (v1.2.6) [69]~~[67]~~, nanomonsv (v0.7.1) [70]~~[68]~~, SAVANA (v.1.~~2.~~0.5) [34], and Severus (v1.0) [33]) to call SV using long reads. Each tool uses a pair of tumour and normal bam files and runs with default parameters with SV length less than 50bp were excluded. In addition, nanomonsv included an extra reference panel of 30 human genomes to filter somatic SVs. As part of the nanomonsv pipeline, SV events that overlapped with simple repeats were filtered and the SV type was annotated using the sv_type.py script of the package. Phased and haplotagged bam files were provided to Severus and variable number tandem repeat (VNTR) regions were used in the analysis. Whatshap (v1.4) [71]~~[69]~~ was used to phase genomics variants in germline VCF files and add tags to each read in BAM files with known haplotypes.

4.7 Short-read sequencing for cancer cell lines

DNA was extracted for COLO829 and HCC1937 cells and matched B lymphoblastoid cells using the Qiagen AllPrep DNA mini kit according to the manufacturer’s protocol (Qiagen, Germany). Whole genome analysis was performed on DNA from tumour and BL cells using DNA PCR-free prep and sequenced on a NovaSeq (Illumina).

4.8 The short-read gold standard for SNVs and indels

We used the concordant high-confident somatic SNV calls based on three high-depth short-reads sequencing of COLO829 and HCC1937 as our “gold~~en~~ standard”. The cell lines were cultured from the same purchase within a few passages and thus can avoid extensive sub-clonal somatic mutations. The short-read data of three biological replicates were analysed with our in-house standard Cromwell pipeline to detect somatic SNVs with GATK (v4.0.4.0) [72]~~[70]~~ and qSNP (v2.1.4) [73]~~[71]~~ and somatic small indels with GATK. For merging SNVs, we first split multi-base variants (MNVs) into consecutive SNVs and also split multiallelic

sites into multiple entries using the BCFtools norm module. Next, the intersection of high-confidence SNVs for three libraries was calculated using the BCFtools isec command. Finally, the three VCF files were merged and annotated with the intersection results using BCFtools merge. SNVs that are detected in at least two libraries were used as short-read “gold standard”. The indel gold standard was generated similarly to SNV but without atomisation.

4.9 The short-read gold standard for SVs

For each library of COLO829 and HCC1937, we used Delly, GRIDSS (v2.13.2) [74][72], and LUMPY (v0.3.1) [75][73] to call SV from short-read data. The somatic SVs were extracted from each tool and merged within each library. The SVs that were detected by at least two tools were further merged between libraries for each cell line. Finally, SVs that were shared by at least two libraries were used as the SV gold standard. The merging of SVs is not as straightforward as SNVs. The output from each tool was first converted into a simple VCF format with five SV types (INS, INV, DEL, DUP and TRA), and Jasmine [76][74] was used to merge multiple VCF files and to determine the overlapping.

4.10 EPIC Array data analysis

The intensity data (IDAT) files of each cell line were loaded and analysed with the minfi (v1.48.0) [77][75] R package. Specifically, the raw signals were transformed and normalised as Beta values at each probe CpG position, and the probes that contained an SNP at the CpG interrogations or the single nucleotide extension were excluded. To compare long-read methylation with EPIC array data, we performed liftover for EPIC array results from hg19 to hg38 using R packages GenomicRanges (v1.54.1) [78][76] and rtracklayer (v1.62.0) [79][77] with the chain file from the UCSC genome browser [80][78]. The methylation data was also used to o confirm the complement the VAF-based tumour purity levels within the serial diluted samples. Specifically, differentially methylated estimation, we also evaluated the CpG

sites methylation frequency distributions as a quantitative indicator of tumour purity. CpG sites between the tumour and blood lymphocyte cell lines were determined as probes with Beta values ≥0.7 in tumour and ≤0.3 in BL, or ≤0.3 in tumour and ≥0.7 in BL. The distribution of these CpG sites was then assessed in the samples with different tumour purities for COLO829 and HCC1937. In samples with a mixture of normal blood lymphocytes, the characteristic bimodal distribution of tumour methylation composed of highly methylated and de-methylated CpG sites shifts toward intermediate values. The position with tumour-specific methylation status was extracted, which was defined as where the beta value is ≥ 0.7 in tumour and ≤ 0.3 in normal or ≤ 0.3 in tumour and ≥ 0.7 in normal.

4.11 Benchmarking

We benchmarked the somatic SNVs and indels calling using the Python script compare.py of ClairS. We also used hap.py for comparison which generated similar results. All SNVs and indels with PASS filter were used as the target to calculate the recall, precision, and F1 values with the corresponding golden standard for each cell line and variant type as the truth set. For SV benchmarking, tools had different formats to represent SV events. We converted the breakpoint pairs from different tools into the simple type VCF format and merged them with the short-read gold standard using Jasmine with default parameters, and only SVs with a length greater than 50bp were considered. The public COLO829 SV truth set (Version v4) was downloaded from Zenodo (10.5281/zenodo.7515830) and compared with the results for a 100% tumour purity sample. The recall and the precision rates were calculated as below:

$$Recall = \frac{overlap\ calls\ between\ LRS\ and\ SRS}{total\ calls\ from\ SRS}$$

$$Precision = \frac{overlap\ calls\ between\ LRS\ and\ SRS}{total\ calls\ from\ LRS}$$

$$FN = unique\ calls\ in\ SRS$$

$$FP = unique\ calls\ in\ LRS$$

4.12 Genome regions and stratifications

To investigate the genomic distribution of variants identified as TP, FP and FN, genome stratifications for GRCh38, including low complexity, segmental duplications, low mappability, and GC content outliers, were downloaded from the genome-in-a-bottle (GIAB) consortium (v3.4) [8]. The overlapping status of each variant to regions of different categories was determined in R with the GenomicRanges and VariantAnnotation (v1.48) [81]~~[79]~~ packages. The genome-wide distribution of TP, FP and FN SNVs was visualised ~~examined~~using rainfall plots which were aligned with chromosome ideograms annotated with cytogenetic banding patterns (gieStain) obtained from the UCSC genome browser [82]~~)~~.

4.13 Mutational signatures analysis

Formatted: Font: Italic

All SNV somatic mutations were converted into a 96-channel mutational profile (SBS-96 vector) and were subsequently decomposed into contributions of COSMIC reference catalogue (v2.0, GRCh38) using the R package deconstructSigs (v1.8.0) [83]~~(cite)~~. To evaluate whether LR specific variants, classified as false positives, might ~~nevertheless~~ represent genuine mutational events, we compared their mutational spectra with the short-read gold-standard set. Specifically, a cosine similarity was computed between the two profiles.

Formatted: English (Australia)

4.14~~3~~ Germline leakage estimate

To assess the extent of germline contamination in somatic variant calling, we quantified the proportion of SNVs commonly observed in the general population. Population allele frequencies were retrieved using BCFtools, which was used to query each variant against the gnomAD VCF files (version 4.1) [42]. SNVs with a population allele frequency (AF_grpmax) greater than 0.1 were classified as germline variants.

4.154 Visualisation and IGV check

Data wrangling was implemented in R (v4.3.1) with tidyverse (v1.2.1) [84][80] and visualised using ggplot2 (v3.4.4) [85][81] and ggh4x (v0.2.8) [86][82]. The long-read and short-read specific SV events were extracted as BEDPE files, and the IGV view of regions was generated using igv-reports (v1.12.0) (https://github.com/igvteam/igv-reports).

Data accession

The BAM files for cell line mixtures will be deposited in the European Genome-phenome Archive (EGA) under accession number EGAD00001015628 under study EGAS00001008107. Raw POD5 files are available on request.

Code availability

All code used in this study is available at GitHhub (https://github.com/bakeronit/nanopore_celllines_benchmark), and a versioned release will be archived on Zenodo or equivalent alternatives for long-term accessibility.

Conflict of interests

JVP and NW are co-founders of genomiQa. LL and NW were funded by Oxford Nanopore to present work from this study at meetings. The remaining authors declare that there are no competing interests.

Funding sources

This work was funded by a Cancer Council Queensland (CCQ) Accelerating Collaborative Cancer Research (AACR) Grant (000000027). NW is funded by the National Health and Medical Research Council of Australia (NHMRC) Investigator Grant (2018244). LL is supported by a University Queensland Graduate School Scholarship, QIMR Berghofer PhD Top-up scholarship. This research was performed on QIMR Berghofer computing

infrastructure supported by The Ian Potter Foundation and Australian Cancer Research Foundation (ACRF).

Author contributions

Jia Zhang: Conceptualization, Investigation, Formal Analysis, Methodology, Software, Writing – Original Draft Preparation, Visualisation.

~~Bonnie~~ Ho Yi Wong: Investigation, Resources, Writing - Review and Editing.

Lingchen Liu: Methodology, Writing - Review and Editing,

Lambros T. Koufariotis: Formal Analysis, Resources, Writing - Review and Editing.

Scott Wood: Resources, Writing - Review and Editing.

Nadine Fitzpatrick: Investigation, Resources, Writing - Review and Editing

Jenny Quiatchon: Investigation, Resources, Writing - Review and Editing

Paul Collins: Supervision, Investigation, Resources, Writing - Review and Editing

John V. Pearson: Conceptualization, Supervision, Methodology, Resources, Data Curation, Writing - Review and Editing, Funding Acquisition.

Nicola Waddell: Conceptualization, Supervision, Project Administration, Methodology, Resources, Data Curation, Writing - Review and Editing, Funding Acquisition.

Figure Legends

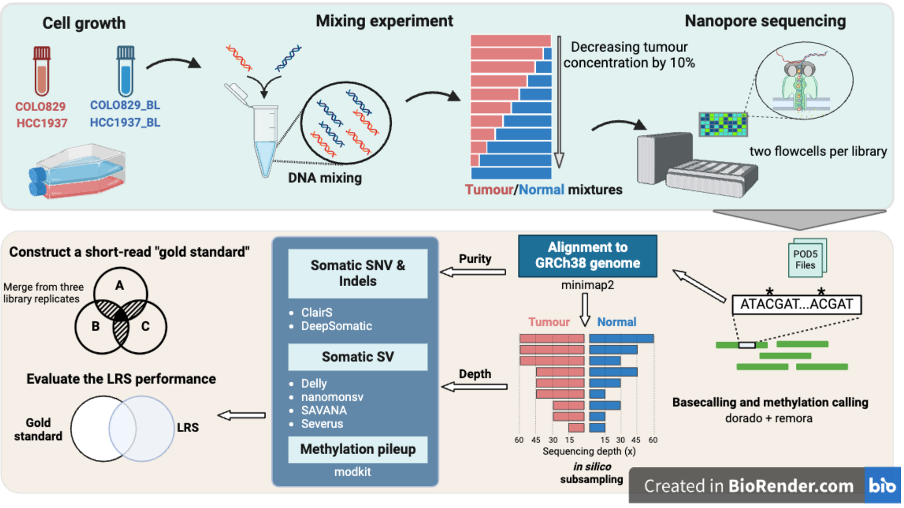

Figure 1: The overview of the experiment design. Two tumour derived cancer cell lines, COLO829 and HCC1937, with their patient matched- non tumour B lymphoblastoid (BL) cell line were cultured and DNA extracted. The DNA was quantified and tumour and non-tumour DNA samples mixed to obtain tumour cell mixtures with decreasing tumour purity by 10% decrements. Libraries for each cell line mixture (n=11 for each tumour cell line) were sequenced with the Nanopore Promethion sequencer with two flow cells per library. The raw data was basecalled with Dorado and the 5mC methylation at CpG sites were called with a matched Remora model during basecalling. Sequence data were alighned to GRCh38 assembly and somatic SNVs, indels, and structural variations identified for samples with different tumour purity levels. Sequencing depths were simulated by subsampling reads at 60x, 45x, 30x, and 15x for samples with 100%, 80%, 60% and 40% tumour purity levels. These combinations sequencing depths (n=9 per tumour cell line) were used for somatic SNV, indels, and SV detection. Short-read gold standard somatic mutations were constructed by overlapping high confident sets from short-read sequencing of three replicate libraries for each cell line. Finally, the somatic variation results were compared with the short-read gold standard to evaluate the performance of long-read sequencing under different tumour purity and depths. The figure was created using BbioRrender (https://www.biorender.com).

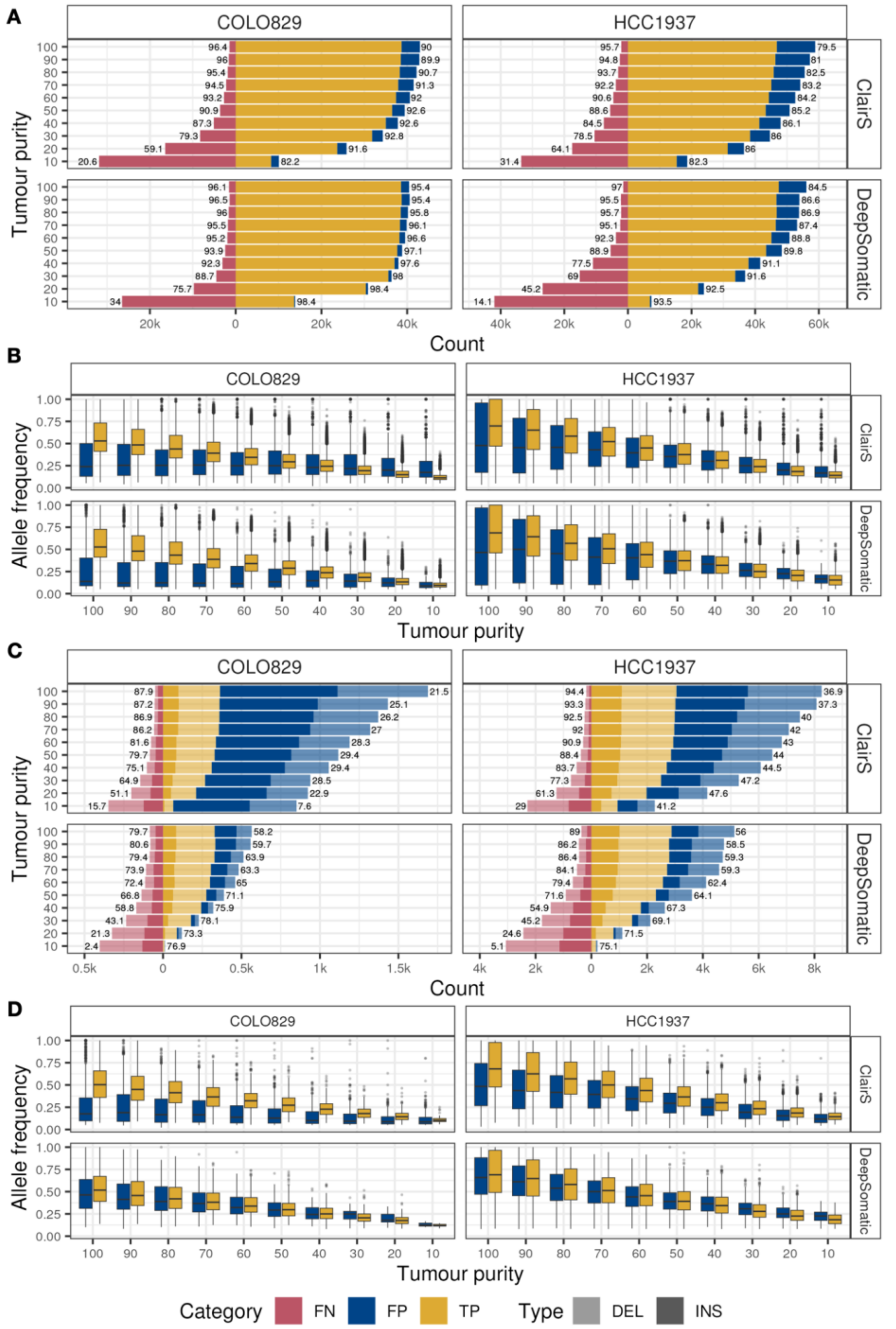
A
B
C
D
COLO829
HCC1937
ClairS
DeepSomatic
Tumour purity
Allele frequency
Count
Category FN FP TP
Type DEL INS

Figure 2: Tumour purity affects somatic SNV and INDEL calling. (a) Number of true positive (TP), false positive (FP) and false negative (FN) SNV calls in COLO829 and HCC1937 tumour cell mixtures using ClairS and DeepSomatic. The size of each call was present on the right side of 0 on the x-axis, and the missed call size on the left side. Precision and Recall rates are labelled at the edges of the right and left sides of each row. (b) Boxplots comparing the distribution of variant allele frequency (VAF) for FP and TP in SNV calling with decreasing tumour purity. (c) The bar plot displays the number of TP, FP and FN indel calls. The pale colour represents the deletions, and the solid colour shows the insertions. Precision and Recall rates are labelled at the edges of the right and left sides of each row. (d) Boxplots comparing the distribution of VAF for FP and TP in indel calling with decreasing tumour purity.

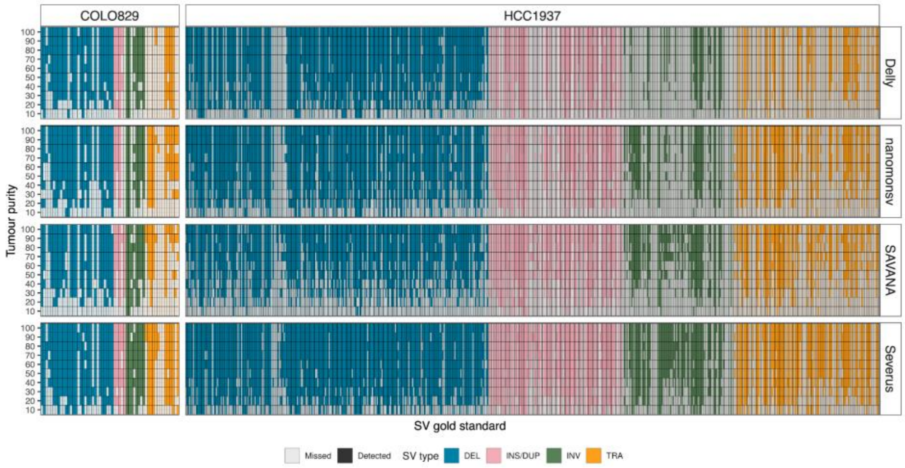

Figure 3: Tumour purity affects somatic SV calling. (a) A heatmap of short-read “gold standard” SV events that are detected by long read using four SV callers in decreasing tumour purity levels for COLO829 and HCC1937 tumour cell lines. SVs of different SV types (INS/DUP, DEL, INV, TRA) are filled with different colours.

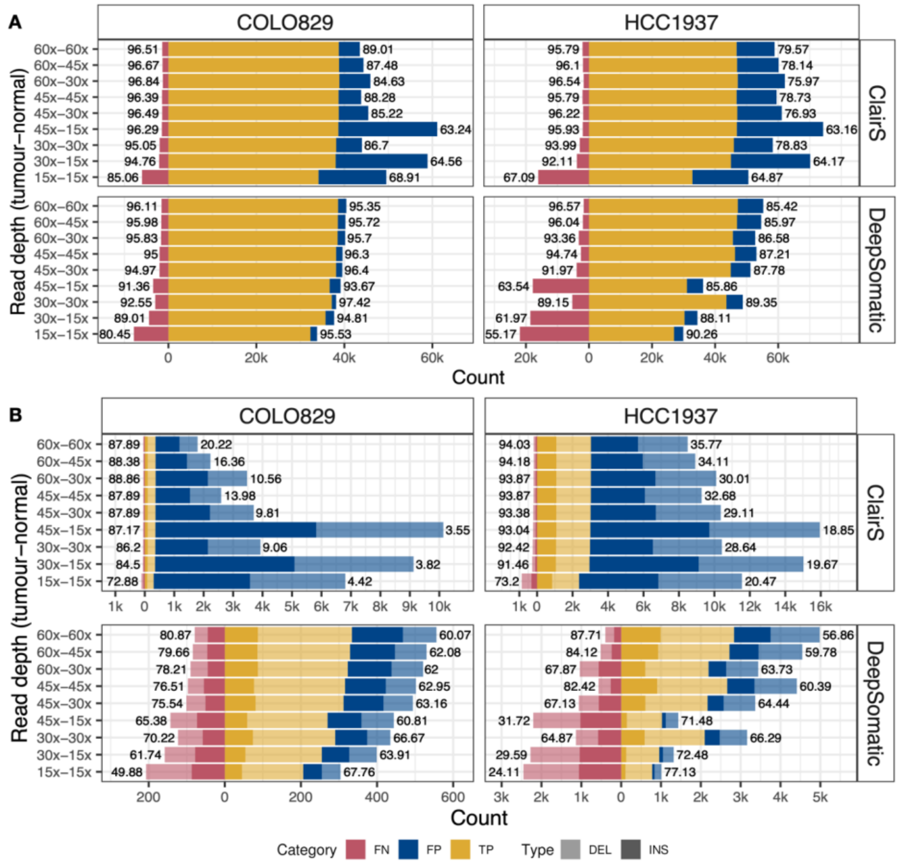

Figure 4: Sequencing depth affects somatic SNV and indel calling. Number of true positive, false positive, and false negative somatic variant calls under nine depth combinations (y-axis) of tumour and normal samples in COLO829 and HCC1937 using ClairS and DeepSomatic with varying tumour purity (100%, 80%, 60% and 40%). (A) Somatic SNV calling performance in recall and precision of ClairS and DeepSomatic. (B) Somatic indel calling performance in recall and precision of ClairS and DeepSomatic, deletions and insertions were filled with stacked bars in different alpha.

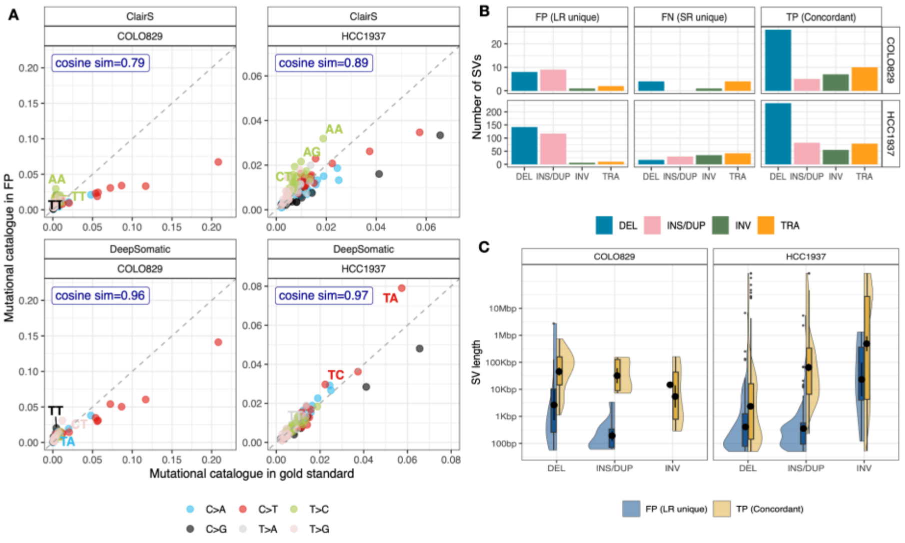

Figure 5: Potential novel events detected by LRS. (A) The comparison of the 96 mutational catalogues between the short-read gold standard and FP from LRS. (B) The number of SVs in four types (DEL: deletion, INS/DUP: insertion/duplication, INV: inversion, TRA: translocation) from FP, FN, and TP. (C) The SV length distribution of FP and TP events in DEL, INS/DUP and INV types, the black dot in each bar indicates the mean value.

References

1. Alexandrov LB, Kim J, Haradhvala NJ, Huang MN, Tian Ng AW, Wu Y, et al.. The repertoire of mutational signatures in human cancer. *Nature*. Springer Science and Business Media LLC; 578:94–1012020;

2. Hanahan D. Hallmarks of cancer: New dimensions. *Cancer Discov*. American Association for Cancer Research (AACR); 12:31–462022;

3. ICGC/TCGA Pan-Cancer Analysis of Whole Genomes Consortium. Pan-cancer analysis of whole genomes. *Nature*. Springer Science and Business Media LLC; 578:82–932020;

4. Yokoyama A, Kakiuchi N, Yoshizato T, Nannya Y, Suzuki H, Takeuchi Y, et al.. Age-related remodelling of oesophageal epithelia by mutated cancer drivers. *Nature*. Springer Science and Business Media LLC; 565:312–72019;

5. Ramarao-Milne P, Kondrashova O, Patch A-M, Nones K, Koufariotis LT, Newell F, et al.. Comparison of actionable events detected in cancer genomes by whole-genome sequencing, in silico whole-exome and mutation panels. *ESMO Open*. 7:1005402022;

Field Code Changed

Formatted: Font: Italic

Formatted: Font: Italic

Formatted: Font: Italic

Formatted: Font: Italic

Formatted: Font: Italic

6. Lee JK, Sivakumar S, Schrock AB, Madison R, Fabrizio D, Gjoerup O, et al.. Comprehensive pan-cancer genomic landscape of KRAS altered cancers and real-world outcomes in solid tumors. *NPJ Precis Oncol*. Springer Science and Business Media LLC; 6:912022;

7. Chakravarty D, Solit DB. Clinical cancer genomic profiling. *Nat Rev Genet*. Springer Science and Business Media LLC; 22:483–5012021;

8. Dwarshuis N, Kalra D, McDaniel J, Sanio P, Alvarez Jerez P, Jadhav B, et al.. The GIAB genomic stratifications resource for human reference genomes. *Nat Commun*. Springer Science and Business Media LLC; 15:90292024;

9. Ermini L, Driguez P. The application of long-read sequencing to cancer. *Cancers (Basel)*. 2024; doi: 10.3390/cancers16071275.

10. Mahmoud M, Gobet N, Cruz-Dávalos DI, Mounier N, Dessimoz C, Sedlazeck FJ. Structural variant calling: the long and the short of it. *Genome Biol*. Springer Science and Business Media LLC; 20:2462019;

11. Rhoads A, Au KF. PacBio Sequencing and its applications. *Genomics Proteomics Bioinformatics*. Oxford University Press (OUP); 13:278–892015;

12. Wang Y, Zhao Y, Bollas A, Wang Y, Au KF. Nanopore sequencing technology, bioinformatics and applications. *Nat Biotechnol*. Springer Science and Business Media LLC; 39:1348–652021;

13. Huddleston J, Ranade S, Malig M, Antonacci F, Chaisson M, Hon L, et al.. Reconstructing complex regions of genomes using long-read sequencing technology. *Genome Res*. Cold Spring Harbor Laboratory; 24:688–962014;

14. Nurk S, Koren S, Rhie A, Rautiainen M, Bzikadze AV, Mikheenko A, et al.. The complete sequence of a human genome. *Science*. American Association for the Advancement of Science (AAAS); 376:44–532022;

15. Kuleshov V, Xie D, Chen R, Pushkarev D, Ma Z, Blauwkamp T, et al.. Whole-genome haplotyping using long reads and statistical methods. *Nat Biotechnol*. Springer Science and Business Media LLC; 32:261–62014;

16. Sakamoto Y, Sereewattanawoot S, Suzuki A. A new era of long-read sequencing for cancer genomics. *J Hum Genet*. Springer Science and Business Media LLC; 65:3–102020;

17. Nattestad M, Goodwin S, Ng K, Baslan T, Sedlazeck FJ, Rescheneder P, et al.. Complex rearrangements and oncogene amplifications revealed by long-read DNA and RNA sequencing of a breast cancer cell line. *Genome Res*. Cold Spring Harbor Laboratory; 28:1126–352018;

18. Cui X, Lin Q, Chen M, Wang Y, Wang Y, Wang Y, et al.. Long-read sequencing unveils novel somatic variants and methylation patterns in the genetic information system of early lung cancer. *Comput Biol Med*. Elsevier BV; 171:1081742024;

19. Xu L, Wang X, Lu X, Liang F, Liu Z, Zhang H, et al.. Long-read sequencing identifies novel structural variations in colorectal cancer. *PLoS Genet*. 19:e10105142023;

20. O'Neill K, Pleasance E, Fan J, Akbari V, Chang G, Dixon K, et al.. Long-read sequencing of an advanced cancer cohort resolves rearrangements, unravels haplotypes, and reveals methylation landscapes. *Cell Genom*. Elsevier BV; 4:1006742024;

Formatted: Font: Italic (×15, margin comments)

21. Brändl B, Steiger M, Kubelt C, Rohrandt C, Zhu Z, Evers M, et al.. Rapid brain tumor classification from sparse epigenomic data. *Nat Med*. Nature Publishing Group; 31:840–82025;

Formatted: Font: Italic

22. Patel A, Göbel K, Ille S, Hinz F, Schoebe N, Bogumil H, et al.. Prospective, multicenter validation of a platform for rapid molecular profiling of central nervous system tumors. *Nat Med*. Nature Publishing Group; :1–11 2025;

Formatted: Font: Italic

23. Amarasinghe SL, Ritchie ME, Gouil Q. Long-read-tools.org: An interactive catalogue of analysis methods for long-read sequencing data. *Gigascience*. Oxford University Press (OUP); 2021; doi: 10.1093/gigascience/giab003.

Formatted: Font: Italic

24. Jain M, Koren S, Miga KH, Quick J, Rand AC, Sasani TA, et al.. Nanopore sequencing and assembly of a human genome with ultra-long reads. *Nat Biotechnol*. 36:338–452018;

Formatted: Font: Italic

25. Sedlazeck FJ, Lee H, Darby CA, Schatz MC. Piercing the dark matter: bioinformatics of long-range sequencing and mapping. *Nat Rev Genet*. Springer Science and Business Media LLC; 19:329–462018;

Formatted: Font: Italic

26. LoTempio J, Delot E, Vilain E. Benchmarking long-read genome sequence alignment tools for human genomics applications. *PeerJ*. 11:e165152023;

Formatted: Font: Italic

27. Liu L, Zhang J, Wood S, Newell F, Leonard C, Koufariotis LT, et al.. Performance of somatic structural variant calling in lung cancer using Oxford Nanopore sequencing technology. *BMC Genomics*. Springer Science and Business Media LLC; 25:8982024;

Formatted: Font: Italic

28. Sigurpalsdottir BD, Stefansson OA, Holley G, Beyter D, Zink F, Hardarson MÞ, et al.. A comparison of methods for detecting DNA methylation from long-read sequencing of human genomes. *Genome Biol*. Springer Science and Business Media LLC; 25:692024;

Formatted: Font: Italic

29. Dong X, Du MRM, Gouil Q, Tian L, Jabbari JS, Bowden R, et al.. Benchmarking long-read RNA-sequencing analysis tools using in silico mixtures. *Nat Methods*. Springer Science and Business Media LLC; 20:1810–212023;

Formatted: Font: Italic

30. Liu Y, Rosikiewicz W, Pan Z, Jillette N, Wang P, Taghbalout A, et al.. DNA methylation-calling tools for Oxford Nanopore sequencing: a survey and human epigenome-wide evaluation. *Genome Biol*. 22:2952021;

Formatted: Font: Italic

31. Park J, Cook DE, Chang P-C, Kolesnikov A, Brambrink L, Mier JC, et al.. DeepSomatic: Accurate somatic small variant discovery for multiple sequencing technologies. bioRxivorg.

32. Zheng Z, Su J, Chen L, Lee Y-L, Lam T-W, Luo R. ClairS: a deep-learning method for long-read somatic small variant calling. bioRxiv.

33. Keskus A, Bryant A, Ahmad T, Yoo B, Aganezov S, Goretsky A, et al.. Severus: accurate detection and characterization of somatic structural variation in tumor genomes using long reads. *medRxiv*. :2024.03.22.243047562024;

Formatted: Font: Italic

34. Elrick H, Sauer CM, Espejo Valle-Inclan J, Trevers K, Tanguy M, Zumalave S, et al.. SAVANA: reliable analysis of somatic structural variants and copy number aberrations in clinical samples using long-read sequencing. bioRxiv.

35. Griffith M, Miller CA, Griffith OL, Krysiak K, Skidmore ZL, Ramu A, et al.. Optimizing cancer genome sequencing and analysis. *Cell Syst*. Elsevier BV; 1:210–232015;

Formatted: Font: Italic

36. Xiao W, Ren L, Chen Z, Fang LT, Zhao Y, Lack J, et al.. Toward best practice in cancer mutation detection with whole-genome and whole-exome sequencing. *Nat Biotechnol*.

Formatted: Font: Italic

39:1141–502021;

37. Yu T, Huang Q, Zhao X, Zhang S, Zhang Q, Fan X, et al.. Tumour purity as an underlying key factor in tumour mutation detection in colorectal cancer. *Clin Transl Med*. Wiley; 13:e12522023;

38. Haider S, Tyekucheva S, Prandi D, Fox NS, Ahn J, Xu AW, et al.. Systematic assessment of tumor purity and its clinical implications. *JCO Precis Oncol*. American Society of Clinical Oncology; 2020; doi: 10.1200/PO.20.00016.

39. Aran D, Sirota M, Butte AJ. Systematic pan-cancer analysis of tumour purity. *Nat Commun*. Springer Science and Business Media LLC; 6:89712015;

40. Mwenifumbo JC, Marra MA. Cancer genome-sequencing study design. *Nat Rev Genet*. Springer Science and Business Media LLC; 14:321–322013;

41. Sendorek DH, Caloian C, Ellrott K, Bare JC, Yamaguchi TN, Ewing AD, et al.. Germline contamination and leakage in whole genome somatic single nucleotide variant detection. *BMC Bioinformatics*. Springer Science and Business Media LLC; 19:282018;

42. Chen S, Francioli LC, Goodrich JK, Collins RL, Kanai M, Wang Q, et al.. A genomic mutational constraint map using variation in 76,156 human genomes. *Nature*. 625:92–1002024;

43. Espejo Valle-Inclan J, Besselink NJM, de Bruijn E, Cameron DL, Ebler J, Kutzera J, et al.. A multi-platform reference for somatic structural variation detection. *Cell Genom*. Elsevier BV; 2:1001392022;

44. Yuen ZW-S, Srivastava A, Daniel R, McNevin D, Jack C, Eyras E. Systematic benchmarking of tools for CpG methylation detection from nanopore sequencing. *Nat Commun*. Springer Science and Business Media LLC; 12:34382021;

45. Pardo-Palacios FJ, Wang D, Reese F, Diekhans M, Carbonell-Sala S, Williams B, et al.. Systematic assessment of long-read RNA-seq methods for transcript identification and quantification. *Nat Methods*. Springer Science and Business Media LLC; 21:1349–632024;

46. Alioto TS, Buchhalter I, Derdak S, Hutter B, Eldridge MD, Hovig E, et al.. A comprehensive assessment of somatic mutation detection in cancer using whole-genome sequencing. *Nat Commun*. Springer Science and Business Media LLC; 6:100012015;

47. Poplin R, Chang P-C, Alexander D, Schwartz S, Colthurst T, Ku A, et al.. A universal SNP and small-indel variant caller using deep neural networks. *Nat Biotechnol*. Springer Science and Business Media LLC; 36:983–72018;

48. Le MK, Qin Q, Li H. Long-range somatic structural variation calling from matched tumor-normal co-assembly graphs. bioRxiv.

49. De La Cerda GY, Landis JB, Eifler E, Hernandez AI, Li F-W, Zhang J, et al.. Balancing read length and sequencing depth: Optimizing Nanopore long-read sequencing for monocots with an emphasis on the Liliales. *Appl Plant Sci*. 11:e115242023;

50. Kosugi S, Terao C. Comparative evaluation of SNVs, indels, and structural variations detected with short- and long-read sequencing data. *Hum Genome Var*. Nature Publishing Group; 11:182024;

51. Choo Z-N, Behr JM, Deshpande A, Hadi K, Yao X, Tian H, et al.. Most large structural variants in cancer genomes can be detected without long reads. *Nat Genet*. Springer

Science and Business Media LLC; 55:2139–482023;

52. Kolmogorov M, Billingsley KJ, Mastoras M, Meredith M, Monlong J, Lorig-Roach R, et al.. Scalable Nanopore sequencing of human genomes provides a comprehensive view of haplotype-resolved variation and methylation. *Nat Methods*. 20:1483–922023;

53. Nyaga DM, Tsai P, Gebbie C, Phua HH, Yap P, Le Quesne Stabej P, et al.. Benchmarking nanopore sequencing and rapid genomics feasibility: validation at a quaternary hospital in New Zealand. *NPJ Genom Med*. Springer Science and Business Media LLC; 9:572024;

54. Olson ND, Wagner J, Dwarshuis N, Miga KH, Sedlazeck FJ, Salit M, et al.. Variant calling and benchmarking in an era of complete human genome sequences. *Nat Rev Genet*. Springer Science and Business Media LLC; 24:464–832023;

55. Paulin LF, Fan J, O'Neill K, Pleasance E, Porter VL, Jones SJM, et al.. The benefit of a complete reference genome for cancer structural variant analysis. *medRxiv*. :2024.03.15.243043692024;

56. Ten Berk de Boer E, Ameur A, Bunikis I, Ek M, Stattin E-L, Feuk L, et al.. Long-read sequencing and optical mapping generates near T2T assemblies that resolves a centromeric translocation. *Sci Rep*. 14:90002024;

57. Li H, Bloom JM, Farjoun Y, Fleharty M, Gauthier L, Neale B, et al.. A synthetic-diploid benchmark for accurate variant-calling evaluation. *Nat Methods*. Nature Publishing Group; 15:595–72018;

58. Yang C, Chu J, Warren RL, Birol I. NanoSim: nanopore sequence read simulator based on statistical characterization. *Gigascience*. Oxford Academic; 6:1–62017;

59. Ono Y, Asai K, Hamada M. PBSIM2: a simulator for long-read sequencers with a novel generative model of quality scores. *Bioinformatics*. Oxford University Press (OUP); 37:589–952021;

60. Craig DW, Nasser S, Corbett R, Chan SK, Murray L, Legendre C, et al.. A somatic reference standard for cancer genome sequencing. *Sci Rep*. Springer Science and Business Media LLC; 6:246072016;

61. Ewing AD, Houlahan KE, Hu Y, Ellrott K, Caloian C, Yamaguchi TN, et al.. Combining tumor genome simulation with crowdsourcing to benchmark somatic single-nucleotide-variant detection. *Nat Methods*. Springer Science and Business Media LLC; 12:623–302015;

62. English AC, Dolzhenko E, Ziaei Jam H, McKenzie SK, Olson ND, De Coster W, et al.. Analysis and benchmarking of small and large genomic variants across tandem repeats. *Nat Biotechnol*. Nature Publishing Group; :1–12 2024;

63. . Dorado: Oxford nanopore's basecaller. Github;

64. Li H, Handsaker B, Wysoker A, Fennell T, Ruan J, Homer N, et al.. The Sequence Alignment/Map format and SAMtools. *Bioinformatics*. Oxford University Press (OUP); 25:2078–92009;

65. Li H. New strategies to improve minimap2 alignment accuracy. *Bioinformatics*. Oxford University Press (OUP); 37:4572–42021;

66. Tarasov A, Vilella AJ, Cuppen E, Nijman IJ, Prins P. Sambamba: fast processing of NGS alignment formats. *Bioinformatics*. Oxford University Press (OUP); 31:2032–42015;

67. Pedersen BS, Quinlan AR. Mosdepth: quick coverage calculation for genomes and exomes. *Bioinformatics*. Oxford University Press (OUP); 34:867–82018;

Formatted: Font: Italic

68. Danecek P, Bonfield JK, Liddle J, Marshall J, Ohan V, Pollard MO, et al.. Twelve years of SAMtools and BCFtools. *Gigascience*. Oxford University Press (OUP); 2021; doi: 10.1093/gigascience/giab008.

Formatted: Font: Italic

69. Rausch T, Zichner T, Schlattl A, Stütz AM, Benes V, Korbel JO. DELLY: structural variant discovery by integrated paired-end and split-read analysis. *Bioinformatics*. Oxford University Press (OUP); 28:i333–92012;

Formatted: Font: Italic

70. Shiraishi Y, Koya J, Chiba K, Okada A, Arai Y, Saito Y, et al.. Precise characterization of somatic complex structural variations from tumor/control paired long-read sequencing data with nanomonsv. *Nucleic Acids Res*. Oxford University Press (OUP); 51:e742023;

Formatted: Font: Italic

71. Martin M, Ebert P, Marschall T. Read-based phasing and analysis of phased variants with WhatsHap. *Methods Mol Biol*. 2590:127–382023;

Formatted: Font: Italic

72. Van der Auwera GA, Carneiro MO, Hartl C, Poplin R, del Angel G, Levy-Moonshine A, et al.. From FastQ Data to High-Confidence Variant Calls: The Genome Analysis Toolkit Best Practices Pipeline. *Current Protocols in Bioinformatics*. 43:11.10.1-11.10.332013;

Formatted: Font: Italic

73. Kassahn KS, Holmes O, Nones K, Patch A-M, Miller DK, Christ AN, et al.. Somatic point mutation calling in low cellularity tumors. *PLoS One*. Public Library of Science (PLoS); 8:e743802013;

Formatted: Font: Italic

74. Cameron DL, Baber J, Shale C, Valle-Inclan JE, Besselink N, van Hoeck A, et al.. GRIDSS2: comprehensive characterisation of somatic structural variation using single breakend variants and structural variant phasing. *Genome Biol*. Springer Science and Business Media LLC; 22:2022021;

Formatted: Font: Italic

75. Layer RM, Chiang C, Quinlan AR, Hall IM. LUMPY: a probabilistic framework for structural variant discovery. *Genome Biol*. Springer Nature; 15:R842014;

Formatted: Font: Italic

76. Kirsche M, Prabhu G, Sherman R, Ni B, Battle A, Aganezov S, et al.. Jasmine and Iris: population-scale structural variant comparison and analysis. *Nat Methods*. Springer Science and Business Media LLC; 20:408–172023;

Formatted: Font: Italic

77. Aryee MJ, Jaffe AE, Corrada-Bravo H, Ladd-Acosta C, Feinberg AP, Hansen KD, et al.. Minfi: a flexible and comprehensive Bioconductor package for the analysis of Infinium DNA methylation microarrays. *Bioinformatics*. Oxford University Press (OUP); 30:1363–92014;

Formatted: Font: Italic

78. Lawrence M, Huber W, Pagès H, Aboyoun P, Carlson M, Gentleman R, et al.. Software for computing and annotating genomic ranges. *PLoS Comput Biol*. Public Library of Science (PLoS); 9:e10031182013;

Formatted: Font: Italic

79. Lawrence M, Gentleman R, Carey V. rtracklayer: an R package for interfacing with genome browsers. *Bioinformatics*. Oxford University Press (OUP); 25:1841–22009;

Formatted: Font: Italic

80. Nassar LR, Barber GP, Benet-Pagès A, Casper J, Clawson H, Diekhans M, et al.. The UCSC Genome Browser database: 2023 update. *Nucleic Acids Res*. Oxford University Press (OUP); 51:D1188–952023;

Formatted: Font: Italic

81. Obenchain V, Lawrence M, Carey V, Gogarten S, Shannon P, Morgan M. VariantAnnotation: a Bioconductor package for exploration and annotation of genetic variants. *Bioinformatics*. Oxford University Press (OUP); 30:2076–82014;

Formatted: Font: Italic

82. Perez G, Barber GP, Benet-Pages A, Casper J, Clawson H, Diekhans M, et al.. The UCSC Genome Browser database: 2025 update. *Nucleic Acids Res*. Oxford University Press (OUP); 53:D1243–92025;

83. Rosenthal R, McGranahan N, Herrero J, Taylor BS, Swanton C. DeconstructSigs: delineating mutational processes in single tumors distinguishes DNA repair deficiencies and patterns of carcinoma evolution. *Genome Biol*. Springer Science and Business Media LLC; 17:312016;

84. Wickham H, Averick M, Bryan J, Chang W, McGowan LD, François R, et al.. Welcome to the tidyverse. Journal of Open Source Software.

85. Wickham H. ggplot2: Elegant Graphics for Data Analysis. Springer-Verlag New York;

86. van den Brand T. ggh4x: Hacks for “ggplot2.”1. Alexandrov LB, Kim J, Haradhvala NJ, Huang MN, Tian Ng AW, Wu Y, et al.. The repertoire of mutational signatures in human cancer. *Nature*. Springer Science and Business Media LLC; 578:94–1012020;

2. Hanahan D. Hallmarks of cancer: New dimensions. *Cancer Discov*. American Association for Cancer Research (AACR); 12:31–462022;

3. ICGC/TCGA Pan-Cancer Analysis of Whole Genomes Consortium. Pan-cancer analysis of whole genomes. *Nature*. Springer Science and Business Media LLC; 578:82–932020;

4. Yokoyama A, Kakiuchi N, Yoshizato T, Nannya Y, Suzuki H, Takeuchi Y, et al.. Age-related remodelling of oesophageal epithelia by mutated cancer drivers. *Nature*. Springer Science and Business Media LLC; 565:312–72019;

5. Ramarao-Milne P, Kondrashova O, Patch A-M, Nones K, Koufariotis LT, Newell F, et al.. Comparison of actionable events detected in cancer genomes by whole-genome sequencing, in silico whole-exome and mutation panels. *ESMO Open*. 7:1005402022;

6. Lee JK, Sivakumar S, Schrock AB, Madison R, Fabrizio D, Gjoerup O, et al.. Comprehensive pan-cancer genomic landscape of KRAS altered cancers and real-world outcomes in solid tumors. *NPJ Precis Oncol*. Springer Science and Business Media LLC; 6:912022;

7. Chakravarty D, Solit DB. Clinical cancer genomic profiling. *Nat Rev Genet*. Springer Science and Business Media LLC; 22:483–5012021;

8. Dwarshuis N, Kalra D, McDaniel J, Sanio P, Alvarez Jerez P, Jadhav B, et al.. The GIAB genomic stratifications resource for human reference genomes. *Nat Commun*. Springer Science and Business Media LLC; 15:90292024;

9. Ermini L, Driguez P. The application of long-read sequencing to cancer. *Cancers (Basel)*. 2024; doi: 10.3390/cancers16071275.

10. Mahmoud M, Gobet N, Cruz-Dávalos DI, Mounier N, Dessimoz C, Sedlazeck FJ. Structural variant calling: the long and the short of it. *Genome Biol*. Springer Science and Business Media LLC; 20:2462019;

11. Rhoads A, Au KF. PacBio Sequencing and its applications. *Genomics Proteomics Bioinformatics*. Oxford University Press (OUP); 13:278–892015;

12. Wang Y, Zhao Y, Bollas A, Wang Y, Au KF. Nanopore sequencing technology, bioinformatics and applications. *Nat Biotechnol*. Springer Science and Business Media LLC; 39:1348–652021;

Formatted: Font: Italic

Formatted: Font: Italic

13. Huddleston J, Ranade S, Malig M, Antonacci F, Chaisson M, Hon L, et al.. Reconstructing complex regions of genomes using long-read sequencing technology. *Genome Res*. Cold Spring Harbor Laboratory; 24:688–962014;

14. Nurk S, Koren S, Rhie A, Rautiainen M, Bzikadze AV, Mikheenko A, et al.. The complete sequence of a human genome. *Science*. American Association for the Advancement of Science (AAAS); 376:44–532022;

15. Kuleshov V, Xie D, Chen R, Pushkarev D, Ma Z, Blauwkamp T, et al.. Whole-genome haplotyping using long reads and statistical methods. *Nat Biotechnol*. Springer Science and Business Media LLC; 32:261–62014;

16. Sakamoto Y, Sereewattanawoot S, Suzuki A. A new era of long-read sequencing for cancer genomics. *J Hum Genet*. Springer Science and Business Media LLC; 65:3–102020;

17. Nattestad M, Goodwin S, Ng K, Baslan T, Sedlazeck FJ, Rescheneder P, et al.. Complex rearrangements and oncogene amplifications revealed by long-read DNA and RNA sequencing of a breast cancer cell line. *Genome Res*. Cold Spring Harbor Laboratory; 28:1126–352018;

18. Cui X, Lin Q, Chen M, Wang Y, Wang Y, Wang Y, et al.. Long-read sequencing unveils novel somatic variants and methylation patterns in the genetic information system of early lung cancer. *Comput Biol Med*. Elsevier BV; 171:1081742024;

19. Xu L, Wang X, Lu X, Liang F, Liu Z, Zhang H, et al.. Long-read sequencing identifies novel structural variations in colorectal cancer. *PLoS Genet*. 19:e10105142023;

20. O'Neill K, Pleasance E, Fan J, Akbari V, Chang G, Dixon K, et al.. Long-read sequencing of an advanced cancer cohort resolves rearrangements, unravels haplotypes, and reveals methylation landscapes. *Cell Genom*. Elsevier BV; 4:1006742024;

21. Brändl B, Steiger M, Kubelt C, Rohrandt C, Zhu Z, Evers M, et al.. Rapid brain tumor classification from sparse epigenomic data. *Nat Med*. Nature Publishing Group; 31:840–82025;

22. Patel A, Göbel K, Ille S, Hinz F, Schoebe N, Bogumil H, et al.. Prospective, multicenter validation of a platform for rapid molecular profiling of central nervous system tumors. *Nat Med*. Nature Publishing Group; :1–11 2025;

23. Amarasinghe SL, Ritchie ME, Gouil Q. Long-read-tools.org: An interactive catalogue of analysis methods for long-read sequencing data. *Gigascience*. Oxford University Press (OUP); 2021; doi: 10.1093/gigascience/giab003.

24. Jain M, Koren S, Miga KH, Quick J, Rand AC, Sasani TA, et al.. Nanopore sequencing and assembly of a human genome with ultra-long reads. *Nat Biotechnol*. 36:338–452018;

25. Sedlazeck FJ, Lee H, Darby CA, Schatz MC. Piercing the dark matter: bioinformatics of long-range sequencing and mapping. *Nat Rev Genet*. Springer Science and Business Media LLC; 19:329–462018;

26. LoTempio J, Delot E, Vilain E. Benchmarking long-read genome sequence alignment tools for human genomics applications. *PeerJ*. 11:e165152023;

27. Liu L, Zhang J, Wood S, Newell F, Leonard C, Koufariotis LT, et al.. Performance of somatic structural variant calling in lung cancer using Oxford Nanopore sequencing technology. *BMC Genomics*. Springer Science and Business Media LLC; 25:8982024;

28. Sigurpalsdottir BD, Stefansson OA, Holley G, Beyter D, Zink F, Hardarson MÞ, et al.. A comparison of methods for detecting DNA methylation from long-read sequencing of human genomes. *Genome Biol*. Springer Science and Business Media LLC; 25:692024;

29. Dong X, Du MRM, Gouil Q, Tian L, Jabbari JS, Bowden R, et al.. Benchmarking long-read RNA-sequencing analysis tools using in silico mixtures. *Nat Methods*. Springer Science and Business Media LLC; 20:1810–212023;

30. Liu Y, Rosikiewicz W, Pan Z, Jillette N, Wang P, Taghbalout A, et al.. DNA methylation-calling tools for Oxford Nanopore sequencing: a survey and human epigenome-wide evaluation. *Genome Biol*. 22:2952021;

31. Park J, Cook DE, Chang P-C, Kolesnikov A, Brambrink L, Mier JC, et al.. DeepSomatic: Accurate somatic small variant discovery for multiple sequencing technologies. bioRxivorg.

32. Zheng Z, Su J, Chen L, Lee Y-L, Lam T-W, Luo R. ClairS: a deep-learning method for long-read somatic small variant calling. bioRxiv.

33. Keskus A, Bryant A, Ahmad T, Yoo B, Aganezov S, Goretsky A, et al.. Severus: accurate detection and characterization of somatic structural variation in tumor genomes using long reads. *medRxiv*. :2024.03.22.243047562024;

34. Elrick H, Sauer CM, Espejo Valle-Inclan J, Trevers K, Tanguy M, Zumalave S, et al.. SAVANA: reliable analysis of somatic structural variants and copy number aberrations in clinical samples using long-read sequencing. bioRxiv.

35. Griffith M, Miller CA, Griffith OL, Krysiak K, Skidmore ZL, Ramu A, et al.. Optimizing cancer genome sequencing and analysis. *Cell Syst*. Elsevier BV; 1:210–232015;

36. Xiao W, Ren L, Chen Z, Fang LT, Zhao Y, Lack J, et al.. Toward best practice in cancer mutation detection with whole-genome and whole-exome sequencing. *Nat Biotechnol*. 39:1141–502021;

37. Yu T, Huang Q, Zhao X, Zhang S, Zhang Q, Fan X, et al.. Tumour purity as an underlying key factor in tumour mutation detection in colorectal cancer. *Clin Transl Med*. Wiley; 13:e12522023;

38. Haider S, Tyekucheva S, Prandi D, Fox NS, Ahn J, Xu AW, et al.. Systematic assessment of tumor purity and its clinical implications. *JCO Precis Oncol*. American Society of Clinical Oncology; 2020; doi: 10.1200/PO.20.00016.

39. Aran D, Sirota M, Butte AJ. Systematic pan-cancer analysis of tumour purity. *Nat Commun*. Springer Science and Business Media LLC; 6:89712015;

40. Mwenifumbo JC, Marra MA. Cancer genome-sequencing study design. *Nat Rev Genet*. Springer Science and Business Media LLC; 14:321–322013;

41. Sendorek DH, Caloian C, Ellrott K, Bare JC, Yamaguchi TN, Ewing AD, et al.. Germline contamination and leakage in whole genome somatic single nucleotide variant detection. *BMC Bioinformatics*. Springer Science and Business Media LLC; 19:282018;

42. Chen S, Francioli LC, Goodrich JK, Collins RL, Kanai M, Wang Q, et al.. A genomic mutational constraint map using variation in 76,156 human genomes. *Nature*. 625:92–1002024;

43. Espejo Valle-Inclan J, Besselink NJM, de Bruijn E, Cameron DL, Ebler J, Kutzera J, et al.. A multi-platform reference for somatic structural variation detection. *Cell Genom*. Elsevier

BV; 2:1001392022;

44. Yuen ZW-S, Srivastava A, Daniel R, McNevin D, Jack C, Eyras E. Systematic benchmarking of tools for CpG methylation detection from nanopore sequencing. *Nat Commun*. Springer Science and Business Media LLC; 12:34382021;

45. Pardo-Palacios FJ, Wang D, Reese F, Diekhans M, Carbonell-Sala S, Williams B, et al.. Systematic assessment of long-read RNA-seq methods for transcript identification and quantification. *Nat Methods*. Springer Science and Business Media LLC; 21:1349–632024;

46. Alioto TS, Buchhalter I, Derdak S, Hutter B, Eldridge MD, Hovig E, et al.. A comprehensive assessment of somatic mutation detection in cancer using whole-genome sequencing. *Nat Commun*. Springer Science and Business Media LLC; 6:100012015;

47. Poplin R, Chang P-C, Alexander D, Schwartz S, Colthurst T, Ku A, et al.. A universal SNP and small-indel variant caller using deep neural networks. *Nat Biotechnol*. Springer Science and Business Media LLC; 36:983–72018;

48. Le MK, Qin Q, Li H. Long-range somatic structural variation calling from matched tumor-normal co-assembly graphs. bioRxiv.

49. De La Cerda GY, Landis JB, Eifler E, Hernandez AI, Li F-W, Zhang J, et al.. Balancing read length and sequencing depth: Optimizing Nanopore long-read sequencing for monocots with an emphasis on the Liliales. *Appl Plant Sci*. 11:e115242023;

50. Kosugi S, Terao C. Comparative evaluation of SNVs, indels, and structural variations detected with short- and long-read sequencing data. *Hum Genome Var*. Nature Publishing Group; 11:182024;

51. Choo Z-N, Behr JM, Deshpande A, Hadi K, Yao X, Tian H, et al.. Most large structural variants in cancer genomes can be detected without long reads. *Nat Genet*. Springer Science and Business Media LLC; 55:2139–482023;

52. Kolmogorov M, Billingsley KJ, Mastoras M, Meredith M, Monlong J, Lorig-Roach R, et al.. Scalable Nanopore sequencing of human genomes provides a comprehensive view of haplotype-resolved variation and methylation. *Nat Methods*. 20:1483–922023;

53. Nyaga DM, Tsai P, Gebbie C, Phua HH, Yap P, Le Quesne Stabej P, et al.. Benchmarking nanopore sequencing and rapid genomics feasibility: validation at a quaternary hospital in New Zealand. *NPJ Genom Med*. Springer Science and Business Media LLC; 9:572024;

54. Olson ND, Wagner J, Dwarshuis N, Miga KH, Sedlazeck FJ, Salit M, et al.. Variant calling and benchmarking in an era of complete human genome sequences. *Nat Rev Genet*. Springer Science and Business Media LLC; 24:464–832023;

55. Paulin LF, Fan J, O'Neill K, Pleasance E, Porter VL, Jones SJM, et al.. The benefit of a complete reference genome for cancer structural variant analysis. *medRxiv*. :2024.03.15.243043692024;

56. Ten Berk de Boer E, Ameur A, Bunikis I, Ek M, Stattin E-L, Feuk L, et al.. Long-read sequencing and optical mapping generates near T2T assemblies that resolves a centromeric translocation. *Sci Rep*. 14:90002024;

57. Li H, Bloom JM, Farjoun Y, Fleharty M, Gauthier L, Neale B, et al.. A synthetic-diploid benchmark for accurate variant-calling evaluation. *Nat Methods*. Nature Publishing Group;

15:595–72018;

58. Craig DW, Nasser S, Corbett R, Chan SK, Murray L, Legendre C, et al.. A somatic reference standard for cancer genome sequencing. *Sci Rep*. Springer Science and Business Media LLC; 6:246072016;

59. Ewing AD, Houlahan KE, Hu Y, Ellrott K, Caloian C, Yamaguchi TN, et al.. Combining tumor genome simulation with crowdsourcing to benchmark somatic single-nucleotide-variant detection. *Nat Methods*. Springer Science and Business Media LLC; 12:623–302015;

60. English AC, Dolzhenko E, Ziaei Jam H, McKenzie SK, Olson ND, De Coster W, et al.. Analysis and benchmarking of small and large genomic variants across tandem repeats. *Nat Biotechnol*. Nature Publishing Group; :1–12 2024;

61. . Dorado: Oxford nanopore's basecaller. Github;

62. Li H, Handsaker B, Wysoker A, Fennell T, Ruan J, Homer N, et al.. The Sequence Alignment/Map format and SAMtools. *Bioinformatics*. Oxford University Press (OUP); 25:2078–92009;

63. Li H. New strategies to improve minimap2 alignment accuracy. *Bioinformatics*. Oxford University Press (OUP); 37:4572–42021;

64. Tarasov A, Vilella AJ, Cuppen E, Nijman IJ, Prins P. Sambamba: fast processing of NGS alignment formats. *Bioinformatics*. Oxford University Press (OUP); 31:2032–42015;

65. Pedersen BS, Quinlan AR. Mosdepth: quick coverage calculation for genomes and exomes. *Bioinformatics*. Oxford University Press (OUP); 34:867–82018;

66. Danecek P, Bonfield JK, Liddle J, Marshall J, Ohan V, Pollard MO, et al.. Twelve years of SAMtools and BCFtools. *Gigascience*. Oxford University Press (OUP); 2021; doi: 10.1093/gigascience/giab008.

67. Rausch T, Zichner T, Schlattl A, Stütz AM, Benes V, Korbel JO. DELLY: structural variant discovery by integrated paired-end and split-read analysis. *Bioinformatics*. Oxford University Press (OUP); 28:i333–92012;

68. Shiraishi Y, Koya J, Chiba K, Okada A, Arai Y, Saito Y, et al.. Precise characterization of somatic complex structural variations from tumor/control paired long-read sequencing data with nanomonsv. *Nucleic Acids Res*. Oxford University Press (OUP); 51:e742023;

69. Martin M, Ebert P, Marschall T. Read-based phasing and analysis of phased variants with WhatsHap. *Methods Mol Biol*. 2590:127–382023;

70. Van der Auwera GA, Carneiro MO, Hartl C, Poplin R, del Angel G, Levy-Moonshine A, et al.. From FastQ Data to High-Confidence Variant Calls: The Genome Analysis Toolkit Best Practices Pipeline. *Current Protocols in Bioinformatics*. 43:11.10.1-11.10.332013;

71. Kassahn KS, Holmes O, Nones K, Patch A-M, Miller DK, Christ AN, et al.. Somatic point mutation calling in low cellularity tumors. *PLoS One*. Public Library of Science (PLoS); 8:e743802013;

72. Cameron DL, Baber J, Shale C, Valle-Inclan JE, Besselink N, van Hoeck A, et al.. GRIDSS2: comprehensive characterisation of somatic structural variation using single breakend variants and structural variant phasing. *Genome Biol*. Springer Science and Business Media LLC; 22:2022021;

73. Layer RM, Chiang C, Quinlan AR, Hall IM. LUMPY: a probabilistic framework for structural variant discovery. *Genome Biol*. Springer Nature; 15:R842014;

74. Kirsche M, Prabhu G, Sherman R, Ni B, Battle A, Aganezov S, et al.. Jasmine and Iris: population-scale structural variant comparison and analysis. *Nat Methods*. Springer Science and Business Media LLC; 20:408–172023;

75. Aryee MJ, Jaffe AE, Corrada-Bravo H, Ladd-Acosta C, Feinberg AP, Hansen KD, et al.. Minfi: a flexible and comprehensive Bioconductor package for the analysis of Infinium DNA methylation microarrays. *Bioinformatics*. Oxford University Press (OUP); 30:1363–92014;

76. Lawrence M, Huber W, Pagès H, Aboyoun P, Carlson M, Gentleman R, et al.. Software for computing and annotating genomic ranges. *PLoS Comput Biol*. Public Library of Science (PLoS); 9:e10031182013;

77. Lawrence M, Gentleman R, Carey V. rtracklayer: an R package for interfacing with genome browsers. *Bioinformatics*. Oxford University Press (OUP); 25:1841–22009;

78. Nassar LR, Barber GP, Benet-Pagès A, Casper J, Clawson H, Diekhans M, et al.. The UCSC Genome Browser database: 2023 update. *Nucleic Acids Res*. Oxford University Press (OUP); 51:D1188–952023;

79. Obenchain V, Lawrence M, Carey V, Gogarten S, Shannon P, Morgan M. VariantAnnotation: a Bioconductor package for exploration and annotation of genetic variants. *Bioinformatics*. Oxford University Press (OUP); 30:2076–82014;

80. Wickham H, Averick M, Bryan J, Chang W, McGowan LD, François R, et al.. Welcome to the tidyverse. Journal of Open Source Software.

81. Wickham H. ggplot2: Elegant Graphics for Data Analysis. Springer-Verlag New York;

82. van den Brand T. ggh4x: Hacks for “ggplot2.”

Manuscript

Click here to access/download;Manuscript;Zhang et al main_revision_Clean.docx

Cancer genome standards for long-read sequencing using cancer cell line mixtures

Jia Zhang[1,2], Ho Yi Wong[1,2], Lingchen Liu[1,2], Lambros T. Koufariotis[1], Scott Wood[1], Nadine Fitzpatrick[1], Jenny Quiatchon[1], Paul Collins[1], John V. Pearson[1], Nicola Waddell[1,2]

[1]QIMR Berghofer Medical Research Institute, Brisbane, QLD, Australia

[2] School of Biomedical Sciences, , The University of Queensland, Brisbane, Australia.

Abstract (199 words)

Long-read sequencing (LRS) improves genome alignment and facilitates resolving variants in genomic regions of low complexity, making it a promising approach for cancer variant detection and biomarker discovery. Here, we evaluate the performance of LRS using Oxford Nanopore sequencing to detect somatic variants across different tumour purities and sequencing depths by comparing to somatic variants from short-read sequencing. We generated experimental mixtures of cancer cell lines and matched normal cell lines to simulate 10 tumour purities (ranging from 0 to 100% tumour content). This resulted in 22 samples which were sequenced using whole genome LRS to a targeted depth of 60x and somatic variants identified. We down-sampled data to explore the optimal read depth for somatic variant detection. Our results show long-read variant calling tools achieve recall rates comparable to short-read gold standards. While tumour sequencing depth of 30x to 60x is generally sufficient for detecting common variants, particularly structural variants, sequencing the matched normal sample at adequate depth is crucial for accuracy. Notably, we found variants unique to LRS that may represent real and previously undetected events. This study highlights key factors for optimising cancer genome sequencing with LRS and provides a comprehensive dataset of cell line mixtures for future research and tool development.

Keywords

Long read sequencing, Oxford Nanopore Technologies, somatic mutation, tumour purity, sequencing depth

1. Introduction

Cancer is a complex and multifaceted disease characterised by diverse genetic and epigenetic alterations, such as single nucleotide variations (SNVs), structural variations (SVs), and changes in base methylation [1–3]. These genomic changes drive abnormal cellular proliferation and carcinogenesis [4], and represent targets for treatment [5] making the detection of mutations in tumour cells crucial for cancer research and precision therapy [6,7]. While high-throughput short-read sequencing (SRS) has been widely used to identify somatic mutations across various cancer types, it often fails to detect variations in low-complexity, highly repetitive, or GC-biased regions due to limited mappability [8–10]. Long-read sequencing (LRS) technologies, such as those developed by Pacific Biosciences (PacBio) [11] and Oxford Nanopore Technologies (ONT) [12], offer extended read lengths that can span highly repetitive genomic regions effectively [13]. Initially used to enhance reference genome assembly and to resolve haplotypes [14,15], LRS is increasingly applied in cancer genomics due to its ability to identify novel variations in previously unresolved regions of tumour genomes [16–20]. Recent studies using high-throughput platforms like the Nanopore PromethION have enabled rapid and comprehensive profiling of various advanced cancer cohorts [20] and central nervous system tumours [21,22], demonstrating significant potential for clinical applications. However, the use of LRS in cancer has largely been restricted to a small number of studies or the analysis of cell lines, which underscores the importance of benchmarking these technologies to optimise their clinical and research use.

The evolution of LRS technologies has led to a proliferation of approaches for analysing the resulting data [23], including basecalling, alignment and variant calling. LRS requires computational methods that are distinct from SRS methods due to its longer read length and unique error profiles [24,25]. Benchmarking studies have evaluated various LRS tools to

analyse cancer genome data, including aligners, somatic structural variation detection and methylation detection methods [26–28]. However, many of these evaluations relied on *in-silico* data or sequence data from cell-lines [29,30], meaning they did not capture complexities of tumour tissue samples such as tumour purity. Notably, some benchmarking efforts are published alongside the development of new tools, which can introduce bias as optimal parameters may not be used for each tool [31–34]. Therefore, independent benchmarking is valuable with the continuously emerging new tools and frequent version updates for existing tools [35].

Tumour purity and sequencing depth have a significant impact on the data quality and interpretation in tumour genomics studies [36–38]. Tumour purity refers to the proportion of cancer cells in a sample and it can affect the signal-to-noise ratio, particularly for detecting somatic alterations (mutations specific to the cancer cells in a sample). Low tumour purity can mask somatic mutations by lowering the somatic variant allele frequency, potentially leading to false negatives [39]. Sequencing depth determines the sensitivity for identifying low-frequency variants as well as the ability to distinguish signals from germline cells, therefore, sequencing depth is particularly crucial in heterogeneous tumours [35] where low tumour purity and sub clonal mutations are prevalent. Together, tumour purity and read depth influence the ability to detect somatic mutations, analyse clonal evolution, and understand tumour heterogeneity. Inadequate tumour purity or sequencing depth can result in the incorrect detection of clinically relevant alterations, which will negatively impact genomic discovery for cancer research and precision medicine [38,40]. The implications of tumour purity and sequencing depth for LRS methods have not been explored.

In this study, we created cancer reference samples from two pairs of matched tumour-normal cell lines, to measure the impact of tumour purity (0 to 100%) and sequence depth on somatic mutation detection. We sequenced the samples to an average read depth of >60x using Oxford Nanopore long-read sequencing. We compared the somatic variant calls from different experimental settings against our SRS gold standard to determine optimal

sequencing strategies. The results establish guidelines for expected data quality based on differing tumour purity levels and sequencing depths, providing valuable insights for optimising experimental design in tumour sequencing studies. Furthermore, the generated dataset serves as a valuable resource for the development and validation of bioinformatic tools in this rapidly evolving field.

2. Results

2.1 Study design and overview

We generated two tumour-normal paired cancer samples to simulate different tumour purities and sequence read depths to evaluate the ability of LRS to identify somatic mutations (**Figure 1**). We cultured two cell lines representing distinct tumour types (melanoma, COLO829 and breast cancer, HCC1937) and a matched non-tumour cell line for each. DNA was extracted from each cell line and matched tumour and non-tumour derived DNA were serially mixed to simulate 10 tumour purity levels at 10% increments (0 to 100%). This resulted in 10 tumour mixtures and one matched blood lymphocyte (BL, as a germline/normal control) sample per cell line pair. A sequencing library was made for each sample and sequenced with the PromethION from Oxford Nanopore technologies using two flow cells per library (R10.4.1). Basecalling was performed using Dorado (v0.5.1) with the super accuracy (SUP) model. The mean and median read quality scores were 21.8 and 23. The average yield per flow cell was 126 Gb, and the read length N50 was 10.4 Kb (**Supplementary Figure S1A**).

Two experiments were designed to assess the impact of common variables on somatic mutation calling in cancer samples: tumour purity and sequence read depth. To assess the impact of tumour purity, the 10 tumour cell mixtures and BL germline samples were sequenced to a mean depth of 70.61x for COLO829 (range 58.29x to 79.13x) and 61.57x for HCC1937 (range 53.42x to 71.14x) (**Supplementary Figure S1B),** resulting in >95% of the genome being covered by in at least 1 read (**Supplementary Figure S1C**). The N50

(median aligned read length) for each library ranged from 9.4 to 10.4 kb (**Supplementary Figure S1d**). We assessed impact of alignment using different human reference assemblies, CHM13-T2T and GRCh38. Slight improvements were observed using the CHM13-T2T reference with lower sequence divergence between reads and reference sequences and higher mapping rates (**Supplementary Table S1**). However, in our study we used the GRCh38 assembly due to the comprehensive annotations and maximised compatibility with current tools and our short-read gold standard. To evaluate the performance of mutation detection under different sequencing read depths we randomly subsampled sequence reads from four tumour mixtures (100%, 80%, 60% and 40%) per tumour type (COLO829 and HCC1937) and the matched BL sequence data to create tumour and germline BAM files with 60x, 45x, 30x, and 15x sequencing read depths. This resulted in nine combinations of sequencing depths.

In both experiments, we compared multiple approaches to call somatic single nucleotide variants (SNVs), small insertions and deletions (indels) and structural variants (SVs). To assess the accuracy of the mutations detected with the LRS, we created a "gold standard" call from short-read data by sequencing DNA from the two tumour and matched non-tumour cell lines using three short-read replicate libraries. Sequencing resulted in a minimum read depth of 34x in BL and 67x in tumour samples, and we identified 40,422 SNVs, 416 indels, and 57 SVs in the "gold standard" short-read somatic calls for COLO829 and 48,982 SNVs, 3,236 indels and 537 SVs for HCC1937 (**Supplementary Figure S2A-F**).

2.2 Tumour purity impacts somatic mutation detection

Patient tumour tissues have different tumour purities, therefore, we created 10 tumour mixtures for each cell line to explore how tumour purity impacts somatic mutation detection from LRS. Two approaches were used for somatic SNVs and indels detection from long-read data. ClairS [32] and DeepSomatic [31]. The shifted distributions of variant mutation allele frequency (VAF) for SNVs (**Supplementary Figure S3A**) and methylation frequency at CpG

sites that are differentially methylated between the tumour and normal cell line (**Supplementary Figure S3B**) supports the serial dilutions of tumour DNA by normal cell DNA.

Overall, the number of somatic mutations detected by ClairS and DeepSomatic decreased with decreasing tumour purity, resulting in a lower recall (**Figure 2A**). Nevertheless, even at low tumour purity, the precision remained high (>79.5%). DeepSomatic had a higher precision than ClairS for COLO829 (ClairS range from 82.2% to 92.8%, DeepSomatic range 95.4% to 98.4%) and HCC1937 (ClairS range 79.5% to 86.1%, DeepSomatic range 84.5% to 93.5%) (**Figure 2A** and **Supplementary Figure S4**). In terms of recall, DeepSomatic had a higher mean recall at all tumour purity levels except the sample with 100% tumour purity compared to ClairS for COLO829 (ClairS 81.27%, DeepSomatic 86.39%), however, ClairS had a higher mean recall than DeepSomatic in HCC1937 in samples with lower tumour purity (<50%) (ClairS 81.43%, DeepSomatic 77.02%). The majority of the SNVs were detected by both approaches, with 90% of SNV being detected by ClairS and DeepSomatic for the COLO829 non-diluted sample and 87% for HCC1937 (**Supplementary Table S2**). The union of the two approaches achieved higher recall at the cost of introducing more FP events.

The variant allele frequency of true positive (TP, present in “gold standard”) calls decreased with decreasing tumour purity for both COLO829 and HCC1937 (**Figure 2B**) as expected. In contrast, the false positive (FP, absent from “gold standard”) calls for COLO829 do not show a decrease in AF, with FP having a greater range of AF in both COLO829 and HCC1937 at higher tumour purities (**Figure 2B**). Interestingly, for ClairS, the read depth for FP calls was consistently lower than for TP calls (**Supplementary Figure S5A**). This was not the case for DeepSomatic, suggesting that DeepSomatic FP variants might be TP variants missed by SRS meanwhile read depth could be used as a filter to remove real FP events in ClairS. The quality scores were lower for FP events compared to TP for both callers (**Supplementary Figure S5B**). The different patterns observed in allele frequency, read depth and quality

score distributions between TP and FP events suggest that integrating these measures could serve as an efficient filter to reduce FP calls.

The average recall for indels (ClairS 71.62% for COLO829, 80.28% for HCC1937; DeepSomatic 57.85% for COLO829, 62.66% for HCC1937) (**Figure 2C**) was lower than SNV (**Figure 2A**). ClairS had a higher recall for indels at all tumour purity levels than DeepSomatic. Similar to SNVs detection, the number of somatic indels detected by ClairS and DeepSomatic decreased with decreasing tumour purity, resulting in a lower recall (**Figure 2C**). The average precision was significantly lower for indels (ClairS: 24.6% for COLO829, 42.39% for HCC1937; DeepSomatic: 68.55% for COLO829, 64.27% for HCC1937) than for SNVs (ClairS: 90.57% for COLO829, 83.61% for HCC1937; DeepSomatic: 96.87% for COLO829, 89.29% for HCC1937). The ClairS approach consistently had a lower precision in all tumour purities compared to DeepSomatic for both cell lines (**Figure 2C**). There was no difference between the detection of insertion and deletion events (**Figure 2C**).

Similar to the SNVs calls, the mutation allele frequency of true positive (TP, present in “gold standard”) indel calls decreased with decreasing tumour purity for both COLO829 and HCC1937 (**Figure 2D**). The AF of TP indels was higher than the FP calls for ClairS but not DeepSomatic, suggesting that AF could be used as a filter to remove FP indel events in ClairS. The read depth for FP and TP calls was similar for indel events in ClairS and DeepSomatic (**Supplementary Figure S6A**), suggesting that read depth is not useful for determining TP events. The quality scores were lower for FP events compared to TP for both callers (**Supplementary Figure S6B**), suggesting that quality could be used as a filter to remove potential FP calls.

We compared the SV calls under different purities using four SV detection tools in two cell lines. Most SV calls in the short-read “gold standard” are identified with long-read SV callers, however with 3 out of 57 in COLO829 and 87 out of 573 HCC1937 short-read “gold

standard" SVs were not detected by any LR tool in all tumour purity levels (**Figure 3**), suggesting these events may be missed by LRS or false positive calls in the short-read data. Samples with higher purity usually generate more SV calls (**Supplementary Figure S7**), with a decreasing SV count as purity decreases, particularly in HCC1937, where there are more SV events (573, compared to 57 in COLO829). The Severus tool displays the best recall rates (36.84% to 84.21% in COLO829 and 26.18% to 78.53% in HCC1937) at all tumour purity levels for both cell lines. Even at 20% tumour purity, the recall for Severus is 63.16% for COLO829 and 50.79% for HCC1937. In contrast, the recall for other tools at 20% tumour purity for COLO829 and HCC1937, respectively, was 42.11% and 36.47% for Delly, 40.35% and 35.25% for nanomonsv, and 28.07% and 20.59% for SAVANA. The recall rate for SAVANA was comparable to nanomonsv in higher purity samples (>60%), however, SAVANA did not perform as well in lower purity samples (**Supplementary Figure S7**).

2.3 Sequencing depth affects somatic mutation calls

We sub-sampled tumour and germline samples to simulate different read depths (60x, 45x, 30x, and 15x) and then compared the somatic mutation detection under four purities (100%, 80%, 60%, and 40%). This resulted in nine scenarios of tumour-germline sequencing depths under 4 tumour purities (**Figure 1**).

Lower read depth in the germline sample is expected to impact somatic SNVs detection. For ClairS, this resulted in an increase in recall and a decrease in precision, whereas for DeepSomatic, it resulted in a lower recall and a higher precision. When the germline samples were sequenced at 15x sequencing depth, the performance of these approaches was severely impacted. The precision dropped below 65% in both cell lines for ClairS, and the recall also dropped below 65% in HCC1937 for DeepSomatic (**Figure 4A**). Furthermore, we observed the average allele frequency of FP SNVs when the normal sample at 15x depth higher than other read depth combinations (**Supplementary Figure S8**). Similarly, in indel calling, lower sequencing depth in normal samples leads to decreased precision but

increased recall for ClairS except when normal samples had only 15x read depth, whereas DeepSomatic results in reduced recall but improved precision (**Figure 4B**). Interestingly, ClairS demonstrates robustness to the impact in the HCC1937 cell line, while DeepSomatic shows greater robustness in COLO829. Similar results were observed for samples with lower tumour purity (**Supplementary Figure S9**).

Sequencing depth in both tumour and normal samples had a smaller impact on SVs calling than SNVs and indels calling, particularly for nanomonsv and Severus (**Supplementary Figure S10**). Nanomonsv consistently demonstrated the highest precision in four tools across tumour purities, underscoring the importance of incorporating a reference panel to achieve accurate variant detection. Meanwhile, SAVANA is more sensitive not only to tumour purity but also to sequencing depth. In terms of recall, Delly outperformed SAVANA in recall when germline samples were sequenced at 15x, although its recall remained lower than that of nanomonsv and Severus. Severus exhibited the highest recall among the four tools across all depth combinations and detected more LR-specific (FP) SVs than nanomonsv, particularly in scenarios with lower depth in the normal sample. While the LR-specific SVs may represent novel SVs, they could also be read false positives.

2.4 False positives detected by LRS might be true

Despite the high recall rate in variant detection using LRS compared with SRS, we found that many LR-specific false positive calls might be real. The proportions of single substitution signatures (SBS) catalogues extracted in the false positives had high cosine similarity to our short-read gold standard with 0.79 and 0.89 for ClairS, 0.96 and 0.97 for DeepSomatic in COLO829 and HCC1937 (**Figure 5A**). Notably, ClairS detected more T to C (A to G) type mutations with a context AA existing in FP positive calls for both cell lines. We also found that false positive SNVs from ClairS tend to cluster along the centromere and telomere regions (**Supplementary Figure S11 and Supplementary Figure S12**) along chromosomes for both cell lines compared with DeepSomatic (**Supplementary Figure S13**

and Supplementary Figure S14). This suggests that DeepSomatic has more stringent variant calling at difficult genome regions. The genome stratification of false positive SNVs shows that ClairS calls more FPs in tandem repeats and low mappability regions while DeepSomatic calls more FPs in areas of high or low GC%. Meanwhile, the genome stratification of false positive indels displays a similar pattern between the two tools with a higher proportion of FPs overlapped with tandem repeats and genomic regions with GC percentages <30% or > 55% (**Supplementary Figure S15**).

The high-confidence LR SV calls presented in at least two out of four tools also contain many LR-specific SVs. While these SVs may appear as false positives, we expected some of these SVs to be real due to the technical advantage of long-read over short-read. In support of this, we found that a substantial fraction of the LR-specific SVs are located at genomic regions of extreme GC content (GC% <30 or >55) or within low mappability for short reads (**Supplementary Figure S16**). Furthermore, these events are mostly deletions, insertions and duplications with shorter lengths (<10 kb) (**Figure 5B, Figure 5C**), which means they can be sequenced by a single long read with high confidence. Meanwhile, SRS might miss these smaller SVs (<10 kb) due to the restriction of the length of reads and the uncertainty of the pair-end library sizes.

2.5 Germline leakage is more related to the sequencing depth but not tumour purity

Germline leakage refers to germline mutations being misclassified as somatic calls in the variant detection pipeline [41]. We estimate the germline leakage rate by quantifying somatic calls with a maximum population frequency above 0.1 in gnomAD (version 4.1) [42]. While this threshold may not capture all germline variants, we used these values to compare the germline leakage across tumour purity and sequencing depth. We found that the germline leakage rates remain low across tumour purity and start to increase with tumour purity less than 30% for ClairS while decreasing for DeepSomatic (**Supplementary Figure 17A**). Notably, we found that the leakage spiked when the sequencing depth in the germline was

15x, suggesting enough depth in germline samples is crucial for avoiding germline variation contamination in somatic mutation detection (**Supplementary Figure 17B**).

2.6 Benchmarking SV using publicly available gold-standard

Using short-read data to build the SV gold standard is a challenge due to technical limitations of SRS to detect the full set of SVs in the genome. Previously, a COLO829 SV truth set SV including 68 SVs was thoroughly characterised with multiple sequencing platforms, including short-read and long-read technologies [43]. We benchmarked our long-read SVs to this truth set to evaluate how many SVs that were missed by our SR gold standard can be detected by LRS. The recall rates are slightly lower than the results when benchmarking with our SR gold standard (**Supplementary Figure S18**). This suggests our "gold standard" captured most SVs, however, differences exist because our "gold standard" was constructed using single-platform sequencing data and also genetic changes may accumulate in cell line passaging causing cells from different organisations to increasingly differ across time.

3. Discussion

In this study, we sequenced series of *in vitro* cell line mixtures with different tumour purity levels from two cancer types and compared the performance of long-read somatic variant detection tools, including SNVs, indels, and SVs (≥50bp). Additionally, our *in silico* sequencing depth down-sampling allowed us to evaluate the impact of sequencing read depths of tumour and normal samples on variant detection. Our results provide a practical guide for cancer genomic sequencing of clinical samples, and the dataset of sequenced mixtures could be valuable for benchmarking the robustness of variant calling tools under different tumour purity settings.

Previous studies have benchmarked long-read sequencing for variant calling in a range of genomic contexts, but many focused on germline-specific variants or prioritised specific

genomic features such as SVs [27], CpG methylation [44], and mRNA [45], some with the impact of tumour purity addressed by the *in silico* mixing dataset [29]. However, there remains a critical need to benchmark LRS for variant calling in the context of tumour genomics. Unlike germline variants, somatic mutations in tumours exhibit substantial heterogeneity, both within and across cancer types, posing unique challenges for accurate detection [46]. Through experimental mixing DNA of tumour and normal cell lines and *in silico* down-sampling data, we showed that LRS tools can detect >50% of the variants of all kinds from a short-read “gold standard” at tumour purities as low as 20% and for 30x to 60x sequencing depth in tumour. Importantly, our data show that having enough depth in germline sample (>15x) is critical for recall and precision of somatic mutations. These findings can guide parameters for clinical sequencing protocols.

As expected, tumour purity significantly affects somatic mutation detection, with lower purity reducing sensitivity across SNVs, indels, and SVs. This highlights the challenge of identifying rare somatic alleles in samples with significant stromal cell content. However, even at low tumour purity (e.g., 20%), LRS, especially with Severus for SVs, maintains a reasonable recall rate, demonstrating its utility in low-cellularity samples. Although Nanopore sequencing has lower base qualities (Q20–Q30) than the current SRS (Q30–Q40), deep-learning-based SNV callers like ClairS and DeepSomatic effectively mitigate sequencing errors. ClairS combines pileup and full-alignment models using Bi-GRU and ResNet architectures to classify variants as germline, somatic, or artifacts [32]. DeepSomatic, using a CNN-based model, processes alignment pileups as images [47]. These architectural differences likely drive performance differences. DeepSomatic’s higher precision for SNVs and indels suggests a more conservative, error-resistant approach, possibly due to its training data and algorithm design. While ClairS achieves comparable SNV recall and better indel recall, it has lower precision, suggesting it requires stringent downstream filtering of variants based on biological context. Notably, ClairS detects variants in centromeric and telomeric regions, indicating its potential to identify mutations in genomic regions traditionally

inaccessible to short reads. These results suggest that despite lower base qualities, LRS is able to accurately call somatic mutations even in samples with low tumour content.

Among the four long-read SV detection tools evaluated, Severus consistently outperformed Delly, nanomonsv, and SAVANA across varying tumour purities and sequencing depths. Severus maintained high recall even at low tumour purity and sequencing depth (15×), suggesting its ability to detect tumour-specific signals with minimal interference from germline contamination. SAVANA's performance declined at lower tumour purities and showed high sensitivity to reduced sequencing depth, potentially limiting its applicability in clinical settings. However, its recent integration of tumour purity, ploidy, and copy number alteration (CNA) inferences may address these problems [34]. Nanomonsv performed similarly to SAVANA but remained more robust at lower tumour purities and sequencing depths. Its use of an internal human germline SV reference panel makes it more conservative in SV detection, which aligns with previous findings [27,48].

LRS inherently offers advantages for SV detection over short-read approaches. Given that SNVs and small indels detection show comparable performance [49] between long- and short-read technologies [50], a key question is whether LRS enhances SVs discovery by detecting variants missed by SRS. While previous studies suggested that most large SVs in tumour genomes are detectable by short reads, our findings indicate that long reads uniquely capture numerous short-to-intermediate SVs (<10 kb), which were underrepresented in earlier analyses [51]. This further underscores the importance of LRS in tumour genomics, particularly for identifying novel SV markers beyond the resolution of short-read methods.

To enable future cancer genomics studies and translate LRS into the clinic, we need to know what read depth is required to reliably detect somatic mutations. Our sequencing depth down-sampling experiment suggests that balancing coverage between tumour and normal samples can improve somatic variant detection potentially by minimizing germline

contamination. Furthermore, sequencing depths of 15x or less for the germline sample significantly harms somatic variant calling and should be avoid. In our study, we sequenced both tumour and normal samples using two flow cells aim for a read depth of 60x. While we obtain data of ~30x genomic sequencing coverage per flow cell, a study has shown that a single Nanopore Promethion flow cell can generate >33× human genome coverage with ultra-long reads (N50 >35 kb) with optimized DNA processing and library preparation [52]. Assuming that sequencing with a shorter (10Kb) read length N50 produces higher yields per flow cell [49], in future we can reduce sequencing costs by using only one flowcell each for tumour and normal.

Although long reads allow improved mapping in genomic regions of low complexity, the alignment accuracy remains imperfect [53]. Some suggest the reason to be low read accuracy, however, the main factor could be the genome assembly [54]. We used the GRCh38 assembly in this study as it provides more comprehensive clinical annotations for the downstream analyses, however, GRCh38 remains incomplete with unresolved gaps and repetitive sequences of unknown size. Recent studies suggest that using the CHM13-T2T [14] assembly results in better genetic variant detection from more precision alignment, especially in highly repetitive regions such as centromeres and telomeres [55,56]. Future studies exploring the impact of reference assemblies on variant calling performance may provide valuable insights into cancer genome sequencing.

The "gold standard" used in this study was constructed with the SRS with all the limitations that implies. Previously researchers have utilised the synthetic mixtures of two fully homozygous human cell lines to mock somatic mutations [57], and apply read simulators such as NanoSim [58] or pbsim2 [59] to upsample data for validation of variant detection. While this is a good strategy to explore variant calling performance under extreme scenarios, differences in variation calling performance were found when using the real tumour cell line [33], which underscores the heterogeneous nature of cancer samples [60]. A major output of our study is the creation of reference data (with a range of tumour purities) that can be used

by research community. To this end, we deliberately selected two cancer types, one with a high SNV burden, the other with a high SV burden. Establishing references for other key features of cancer genomes, such as subclone mutations, tandem repeats, and complex SVs, will largely facilitate the development of better computational tools [61,62].

This study explored the optimal experimental design for cancer genome sequencing using LRS through creation of tumour purity mixtures. Many LRS analysis tools are still under active development, and while we are far from a definitive "best practice" for cancer variant calling using LRS, we have investigated key aspects key and highlighted directions for improvement. Importantly, we provide a publicly available dataset of LRS cancer cell line mixtures, offering a valuable resource for future tool development and benchmarking in this fast-evolving field.

4. Materials and Methods

4.1 Ethical statement

This work involves the use of human cell lines, which were approved by the QIMR Berghofer Human Research Ethical Committee (P3577 and P3527).

4.2 Cell culture, DNA extraction and mixing

COLO829 (RRID:CVCL_1137), COLO829_BL (RRID:CVCL_1999), HCC1937 (RRID: CVCL_0290) and HCC1937_BL (RRID:CVCL_3281) were maintained in RPMI +10% FBS. All cell lines were tested for mycoplasma and STR profiling. Cells were harvested between passages 8 and 10. Cells were dissociated using 0.25% Trypsin at 37oC for 5 mins and spun at 1200 rpm for 5 mins. Cells were washed once with PBS and kept at -80oC until DNA extraction. DNA was extracted using AllPrep DNA/RNA Mini Kit (802024, Qiagen) according to the manufacturer’s protocols and eluted in TE buffer. To prepare samples with various tumour purities, both tumour and normal DNA were diluted to 50 ng/uL using the TE buffer.

The tumour and its corresponding normal DNA were mixed in specific ratios to create 10 samples that represent tumour purities ranging from 0 to 100% with increments of 10%.

4.3 Library preparation and Oxford nanopore sequencing with Promethion

DNA quantity and quality were assessed using an Implen NanoPhotometer, then run on a TapeStation (Agilent) to evaluate fragment size. High molecular weight DNA was sheared to 10-12 Kb using Covaris g-tubes. Approximately 1 ug of sheared genomic DNA was used to make libraries using the Oxford Nanopore Technologies (ONT) ligation sequencing kit (SQK-LSK114), according to the manufacturer's instructions, except that ligation was extended to 30 minutes. Each library was loaded at approximately 10 fmol on two separate R10.4.1 PromethION flow cells and sequenced for 72 hours on a PromethION P24 sequencer within QIMR Berghofer scientific services.

4.4 Basecalling, methylation calling and alignment

The raw POD5 files from the sequencer were basecalled using Dorado (v0.5.1) [63] with the super accuracy (SUP) model for the R10 flow cell (dna_r10.4.1_e8.2_400bps_sup@v4.3.0). The 5mC and 5hmC methylation were also detected using dorado with the matched remora model (dna_r10.4.1_e8.2_400bps_sup@v4.3.0_5mCG_5hmCG@v1). SAMtools (v1.17) [64] was used to convert the unaligned basecalled BAM file from basecalling to FASTQ format and filter out reads with average quality (QS) <10 before alignment, and minimap2 (v2.26) [65] was used to align reads to the GRCh38 genome assembly (-y –MD -ax map-ont). The aligned bam files were sorted and indexed using SAMtools. The percentage of 5mC methylated reads at CpG sites was calculated using modkit (v2.4.0) pileup based on the MM and ML tags of reads from both strands in the bam files (--combine-strands --cpg).

4.5 Read depth simulation

To evaluate the performance of variant calling under sequencing strategies, we used Sambamba (v1.0.1) [66] to downsample data from the tumour and the matched B lymphoblast bam files with 60x, 45x, 30x, and 15x read depths in samples with 100%, 80%, 60% and 40% tumour purities. Reads were randomly sampled from the aligned BAM files at the proportion based on the expected and original depth. If a BAM file did not reach a 60x read depth, we used the original BAM file (53.42x for the library of HCC1937 cell line at 40% tumour purity). In total, we generated nine combinations of sequencing depths with either higher read depths in tumour samples (60x-45x, 60x-30x, 45x-30x, 45x-15x, 30x-15x) or equal read depths in both tumour and normal (60x-60x, 45x-45x, 30x-30x, 15x-15x). The depth of subsampled BAM files was confirmed with mosdepth (v0.2.9) [67] to check the potential effect of varied read lengths.

4.6 Somatic SNV, indel and SV detection

We used ClairS (v0.1.7) and DeepSomatic (v1.6.0) for SNV and short indel detection in tumour cells. These tools were selected as they are both actively updated and designed to call somatic SNV and indels using a pair of tumour and normal bam files to predict tumour-specific variants using pre-trained deep-learning models. For ClairS, we used the ont_r10_dorado_sup_5khz model for somatic SNV detection and the r1041_e82_400bps_sup_v430 model from rerio for Clair3 (https://github.com/nanoporetech/rerio/tree/master/clair3_models). For DeepSomatic SNV calling, we used default parameters with an internal ONT model provided by Google Health. The final results in VCF were filtered for somatic SNVs with GT="1/1" and FILTER="PASS" using BCFtools (v1.19) [68] as described in the DeepSomatic document.

We used four tools (Delly (v1.2.6) [69], nanomonsv (v0.7.1) [70], SAVANA (v.1.0.5) [34], and Severus (v1.0) [33]) to call SV using long reads. Each tool uses a pair of tumour and normal bam files and runs with default parameters with SV length less than 50bp were excluded. In

addition, nanomonsv included an extra reference panel of 30 human genomes to filter somatic SVs. As part of the nanomonsv pipeline, SV events that overlapped with simple repeats were filtered and the SV type was annotated using the sv_type.py script of the package. Phased and haplotagged bam files were provided to Severus and variable number tandem repeat (VNTR) regions were used in the analysis. Whatshap (v1.4) [71] was used to phase genomics variants in germline VCF files and add tags to each read in BAM files with known haplotypes.

4.7 Short-read sequencing for cancer cell lines

DNA was extracted for COLO829 and HCC1937 cells and matched B lymphoblastoid cells using the Qiagen AllPrep DNA mini kit according to the manufacturer's protocol (Qiagen, Germany). Whole genome analysis was performed on DNA from tumour and BL cells using DNA PCR-free prep and sequenced on a NovaSeq (Illumina).

4.8 The short-read gold standard for SNVs and indels

We used the concordant high-confident somatic SNV calls based on three high-depth short-reads sequencing of COLO829 and HCC1937 as our "gold standard". The cell lines were cultured from the same purchase within a few passages and thus can avoid extensive sub-clonal somatic mutations. The short-read data of three biological replicates were analysed with our in-house standard Cromwell pipeline to detect somatic SNVs with GATK (v4.0.4.0) [72] and qSNP (v2.1.4) [73] and somatic small indels with GATK. For merging SNVs, we first split multi-base variants (MNVs) into consecutive SNVs and also split multiallelic sites into multiple entries using the BCFtools norm module. Next, the intersection of high-confidence SNVs for three libraries was calculated using the BCFtools isec command. Finally, the three VCF files were merged and annotated with the intersection results using BCFtools merge. SNVs that are detected in at least two libraries were used as short-read "gold standard". The indel gold standard was generated similarly to SNV but without atomisation.

4.9 The short-read gold standard for SVs

For each library of COLO829 and HCC1937, we used Delly, GRIDSS (v2.13.2) [74], and LUMPY (v0.3.1) [75] to call SV from short-read data. The somatic SVs were extracted from each tool and merged within each library. The SVs that were detected by at least two tools were further merged between libraries for each cell line. Finally, SVs that were shared by at least two libraries were used as the SV gold standard. The merging of SVs is not as straightforward as SNVs. The output from each tool was first converted into a simple VCF format with five SV types (INS, INV, DEL, DUP and TRA), and Jasmine [76] was used to merge multiple VCF files and to determine the overlapping.

4.10 EPIC Array data analysis

The intensity data (IDAT) files of each cell line were loaded and analysed with the minfi (v1.48.0) [77] R package. Specifically, the raw signals were transformed and normalised as Beta values at each probe CpG position, and the probes that contained an SNP at the CpG interrogations or the single nucleotide extension were excluded. To compare long-read methylation with EPIC array data, we performed liftover for EPIC array results from hg19 to hg38 using R packages GenomicRanges (v1.54.1) [78] and rtracklayer (v1.62.0) [79] with the chain file from the UCSC genome browser [80]. The methylation data was also used to confirm the tumour purity levels within the serial diluted samples. Specifically, differentially methylated CpG sites between the tumour and blood lymphocyte cell lines were determined as probes with Beta values ≥0.7 in tumour and ≤0.3 in BL, or ≤0.3 in tumour and ≥0.7 in BL. The distribution of these CpG sites was then assessed in the samples with different tumour purities for COLO829 and HCC1937.

4.11 Benchmarking

We benchmarked the somatic SNVs and indels calling using the Python script compare.py of ClairS. We also used hap.py for comparison which generated similar results. All SNVs and

indels with PASS filter were used as the target to calculate the recall, precision, and F1 values with the corresponding gold standard for each cell line and variant type as the truth set. For SV benchmarking, tools had different formats to represent SV events. We converted the breakpoint pairs from different tools into the simple type VCF format and merged them with the short-read gold standard using Jasmine with default parameters, and only SVs with a length greater than 50bp were considered. The public COLO829 SV truth set (Version v4) was downloaded from Zenodo (10.5281/zenodo.7515830) and compared with the results for a 100% tumour purity sample. The recall and the precision rates were calculated as below:

$$Recall = \frac{overlap\ calls\ between\ LRS\ and\ SRS}{total\ calls\ from\ SRS}$$

$$Precision = \frac{overlap\ calls\ between\ LRS\ and\ SRS}{total\ calls\ from\ LRS}$$

$$FN = unique\ calls\ in\ SRS$$

$$FP = unique\ calls\ in\ LRS$$

4.12 Genome regions and stratifications

To investigate the genomic distribution of variants identified as TP, FP and FN, genome stratifications for GRCh38, including low complexity, segmental duplications, low mappability, and GC content outliers, were downloaded from the genome-in-a-bottle (GIAB) consortium (v3.4) [8]. The overlapping status of each variant to regions of different categories was determined in R with the GenomicRanges and VariantAnnotation (v1.48) [81] packages. The genome-wide distribution of TP, FP and FN SNVs was visualised using rainfall plots which were aligned with chromosome ideograms annotated with cytogenetic banding patterns (gieStain) obtained from the UCSC genome browser [82].

4.13 Mutational signatures analysis

All SNV somatic mutations were converted into a 96-channel mutational profile (SBS-96 vector) and were subsequently decomposed into contributions of COSMIC reference catalogue (v2.0, GRCh38) using the R package deconstructSigs (v1.8.0) [83]. To evaluate whether LR specific variants, classified as false positives, might represent genuine mutational events, we compared their mutational spectra with the short-read gold-standard set. Specifically, a cosine similarity was computed between the two profiles.

4.14 Germline leakage estimate

To assess the extent of germline contamination in somatic variant calling, we quantified the proportion of SNVs commonly observed in the general population. Population allele frequencies were retrieved using BCFtools, which was used to query each variant against the gnomAD VCF files (version 4.1) [42]. SNVs with a population allele frequency (AF_grpmax) greater than 0.1 were classified as germline variants.

4.15 Visualisation and IGV check

Data wrangling was implemented in R (v4.3.1) with tidyverse (v1.2.1) [84] and visualised using ggplot2 (v3.4.4) [85] and ggh4x (v0.2.8) [86]. The long-read and short-read specific SV events were extracted as BEDPE files, and the IGV view of regions was generated using igv-reports (v1.12.0) (https://github.com/igvteam/igv-reports).

Data accession

The BAM files for cell line mixtures will be deposited in the European Genome-phenome Archive (EGA) under accession number EGAD00001015628 under study EGAS00001008107. Raw POD5 files are available on request.

Code availability

All code used in this study is available at GitHub (https://github.com/bakeronit/nanopore_celllines_benchmark), and a versioned release will be archived on Zenodo or equivalent alternatives for long-term accessibility.

Conflict of interests

JVP and NW are co-founders of genomiQa. LL and NW were funded by Oxford Nanopore to present work from this study at meetings. The remaining authors declare that there are no competing interests.

Funding sources

This work was funded by a Cancer Council Queensland (CCQ) Accelerating Collaborative Cancer Research (AACR) Grant (000000027). NW is funded by the National Health and Medical Research Council of Australia (NHMRC) Investigator Grant (2018244). LL is supported by a University Queensland Graduate School Scholarship, QIMR Berghofer PhD Top-up scholarship. This research was performed on QIMR Berghofer computing infrastructure supported by The Ian Potter Foundation and Australian Cancer Research Foundation (ACRF).

Author contributions

Jia Zhang: Conceptualization, Investigation, Formal Analysis, Methodology, Software, Writing – Original Draft Preparation, Visualisation.

Ho Yi Wong: Investigation, Resources, Writing - Review and Editing.

Lingchen Liu: Methodology, Writing - Review and Editing,

Lambros T. Koufariotis: Formal Analysis, Resources, Writing - Review and Editing.

Scott Wood: Resources, Writing - Review and Editing.

Nadine Fitzpatrick: Investigation, Resources, Writing - Review and Editing

Jenny Quiatchon: Investigation, Resources, Writing - Review and Editing

Paul Collins: Supervision, Investigation, Resources, Writing - Review and Editing

John V. Pearson: Conceptualization, Supervision, Methodology, Resources, Data Curation, Writing - Review and Editing, Funding Acquisition.

Nicola Waddell: Conceptualization, Supervision, Project Administration, Methodology, Resources, Data Curation, Writing - Review and Editing, Funding Acquisition.

Figure Legends

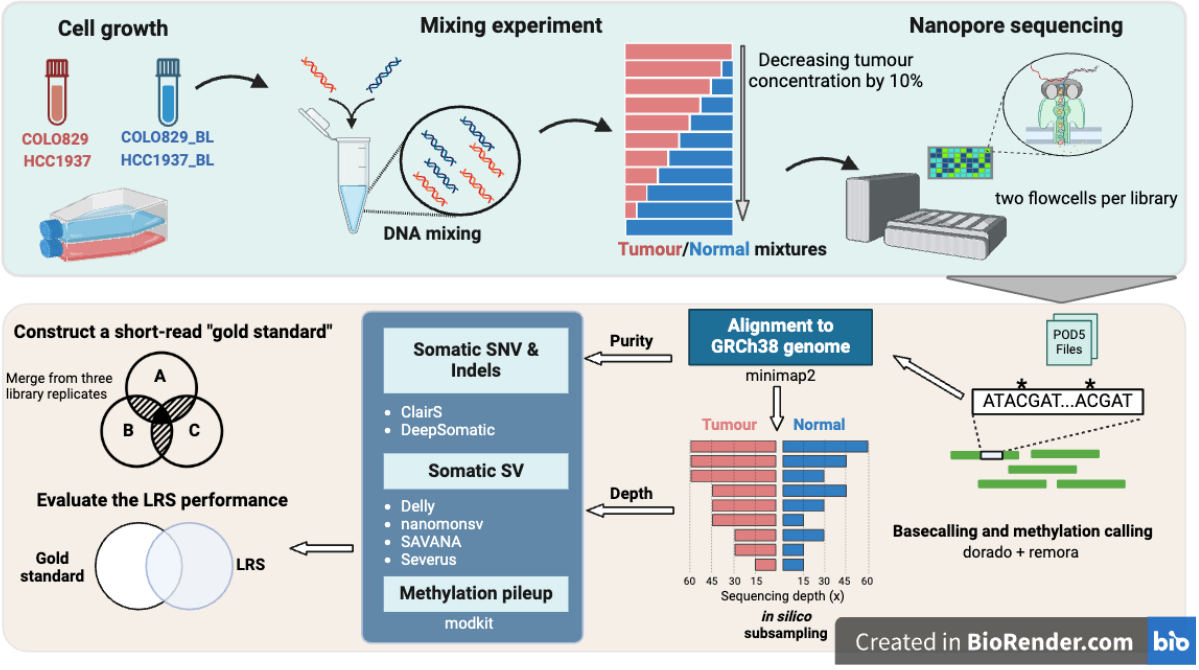

Figure 1: The overview of the experiment design. Two tumour derived cancer cell lines, COLO829 and HCC1937, with their patient matched non tumour B lymphoblastoid (BL) cell line were cultured and DNA extracted. The DNA was quantified and tumour and non-tumour DNA samples mixed to obtain tumour cell mixtures with decreasing tumour purity by 10% decrements. Libraries for each cell line mixture (n=11 for each tumour cell line) were sequenced with the Nanopore Promethion sequencer with two flow cells per library. The raw data was basecalled with Dorado and the 5mC methylation at CpG sites were called with a matched Remora model during basecalling. Sequence data were aligned to GRCh38 assembly and somatic SNVs, indels, and structural variations identified for samples with different tumour purity levels. Sequencing depths were simulated by subsampling reads at 60x, 45x, 30x, and 15x for samples with 100%, 80%, 60% and 40% tumour purity levels. These combinations sequencing depths (n=9 per tumour cell line) were used for somatic SNV, indels, and SV detection. Short-read gold standard somatic mutations were constructed by overlapping high confident sets from short-read

sequencing of three replicate libraries for each cell line. Finally, the somatic variation results were compared with the short-read gold standard to evaluate the performance of long-read sequencing under different tumour purity and depths. The figure was created using BioRender (https://www.biorender.com).

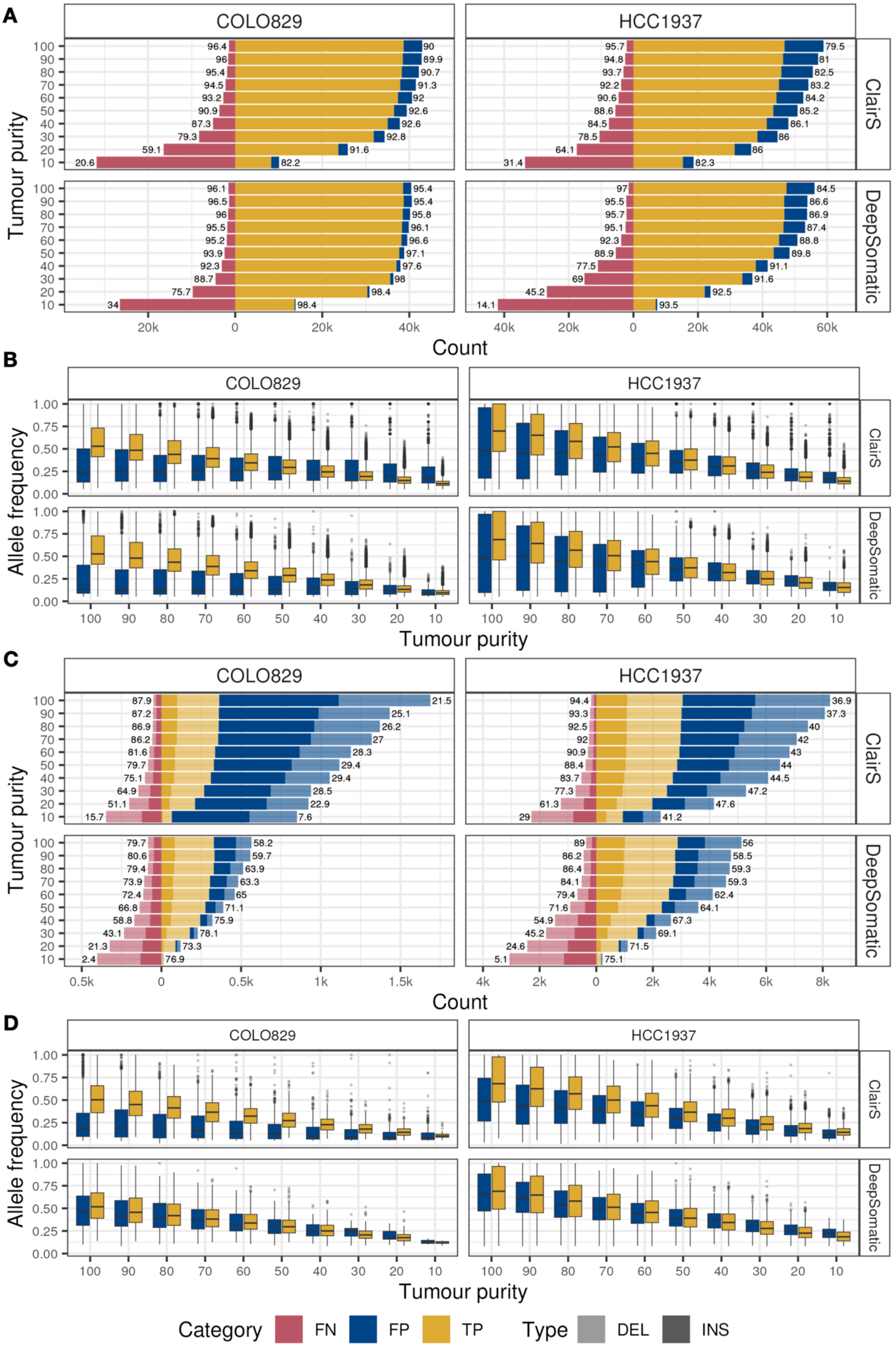
A
COLO829
HCC1937
ClairS
DeepSomatic
Tumour purity
100 90 80 70 60 50 40 30 20 10
96.4 96 95.4 94.5 93.2 90.9 87.3 79.3 59.1 20.6
90 89.9 90.7 91.3 92 92.6 92.6 92.8 91.6 82.2
95.7 94.8 93.7 92.2 90.6 88.6 84.5 78.5 64.1 31.4
79.5 81 82.5 83.2 84.2 85.2 86.1 86 86 82.3
96.1 96.5 96 95.5 95.2 93.9 92.3 88.7 75.7 34
95.4 95.4 95.8 96.1 96.6 97.1 97.6 98 98.4 98.4
97 95.5 95.7 95.1 92.3 88.9 77.5 69 45.2 14.1
84.5 86.6 86.9 87.4 88.8 89.8 91.1 91.6 92.5 93.5
20k 0 20k 40k
40k 20k 0 20k 40k 60k
Count
B
COLO829
HCC1937
ClairS
DeepSomatic
Allele frequency
1.00 0.75 0.50 0.25 0.00
100 90 80 70 60 50 40 30 20 10
Tumour purity
C
COLO829
HCC1937
ClairS
DeepSomatic
Tumour purity
100 90 80 70 60 50 40 30 20 10
87.9 87.2 86.9 86.2 81.6 79.7 75.1 64.9 51.1 15.7
21.5 25.1 26.2 27 28.3 29.4 29.4 28.5 22.9 7.6
94.4 93.3 92.5 92 90.9 88.4 83.7 77.3 61.3 29
36.9 37.3 40 42 43 44 44.5 47.2 47.6 41.2
79.7 80.6 79.4 73.9 72.4 66.8 58.8 43.1 21.3 2.4
58.2 59.7 63.9 63.3 65 71.1 75.9 78.1 73.3 76.9
89 86.2 86.4 84.1 79.4 71.6 54.9 45.2 24.6 5.1
56 58.5 59.3 59.3 62.4 64.1 67.3 69.1 71.5 75.1
0.5k 0 0.5k 1k 1.5k
4k 2k 0 2k 4k 6k 8k
Count
D
COLO829
HCC1937
ClairS
DeepSomatic
Allele frequency
1.00 0.75 0.50 0.25 0.00
100 90 80 70 60 50 40 30 20 10
Tumour purity
Category FN FP TP
Type DEL INS

Figure 2: Tumour purity affects somatic SNV and INDEL calling. (a) Number of true positive (TP), false positive (FP) and false negative (FN) SNV calls in COLO829 and HCC1937 tumour cell mixtures using ClairS and DeepSomatic. The size of each call was present on the right side of 0 on the x-axis, and the missed call size on the left side. Precision and Recall rates are labelled at the edges of the right and left sides of each row. (b) Boxplots comparing the distribution of variant allele frequency (VAF) for FP and TP in SNV calling with decreasing tumour purity. (c) The bar plot displays the number of TP, FP and FN indel calls. The pale colour represents the deletions, and the solid colour shows the insertions. Precision and Recall rates are labelled at the edges of the right and left sides of each row. (d) Boxplots comparing the distribution of VAF for FP and TP in indel calling with decreasing tumour purity.

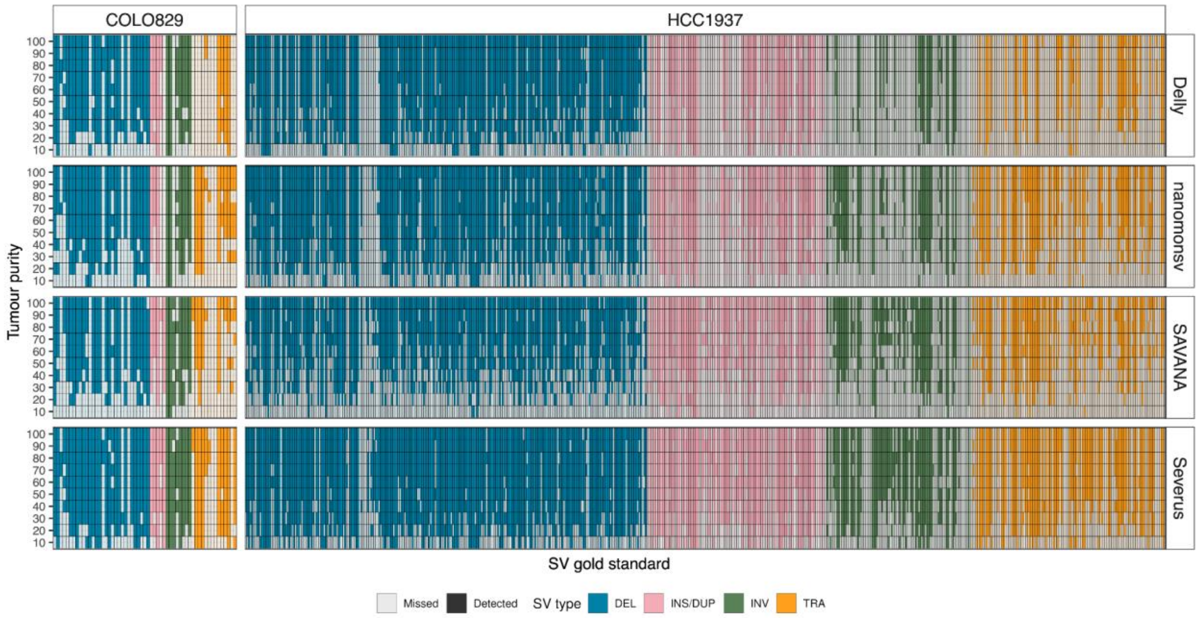

Figure 3: Tumour purity affects somatic SV calling. (a) A heatmap of short-read “gold standard” SV events that are detected by long read using four SV callers in decreasing tumour purity levels for COLO829 and HCC1937 tumour cell lines. SVs of different SV types (INS/DUP, DEL, INV, TRA) are filled with different colours.

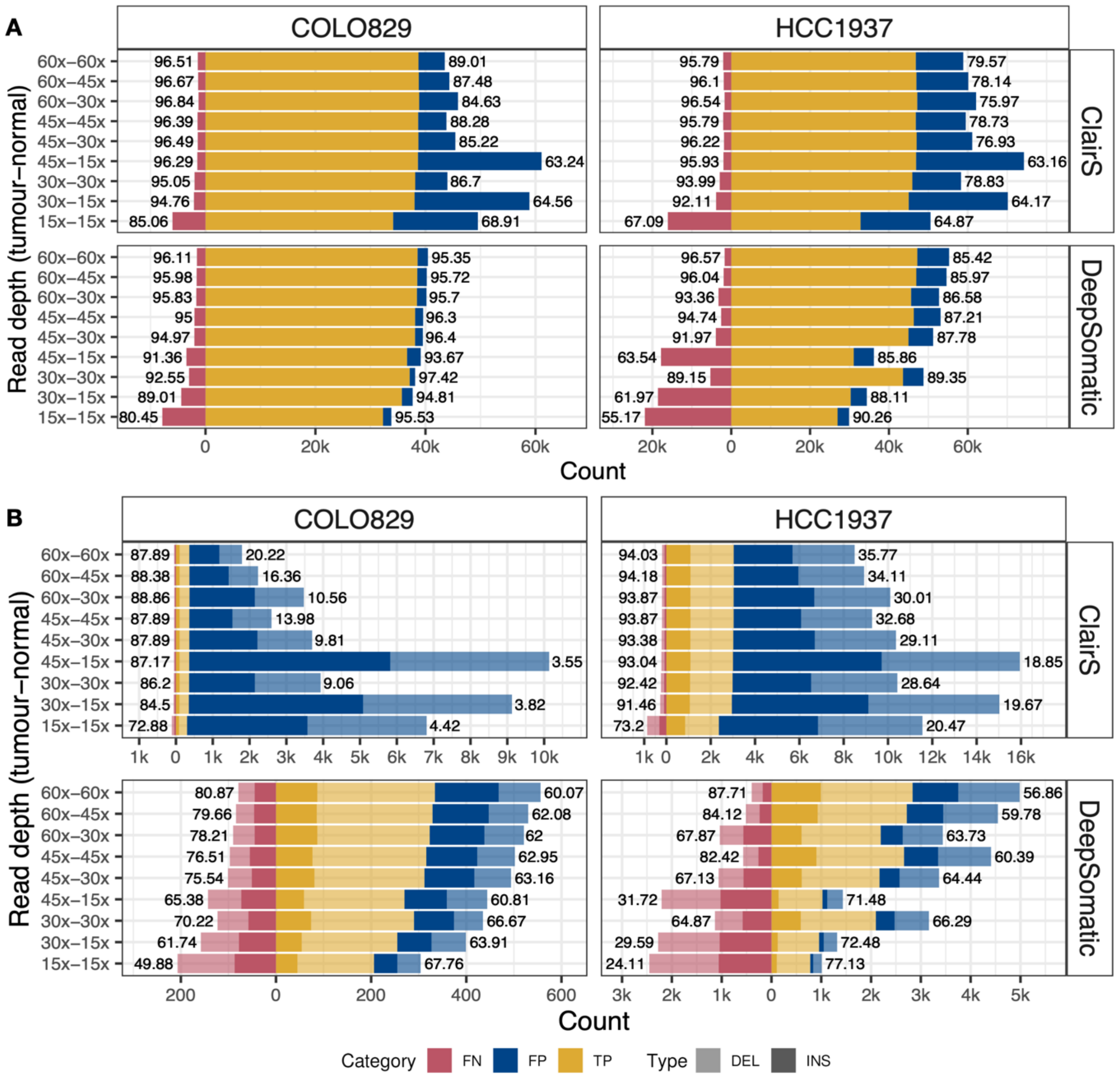

Figure 4: Sequencing depth affects somatic SNV and indel calling. Number of true positive, false positive, and false negative somatic variant calls under nine depth combinations (y-axis) of tumour and normal samples in COLO829 and HCC1937 using ClairS and DeepSomatic with varying tumour purity (100%, 80%, 60% and 40%). (A) Somatic SNV calling performance in recall and precision of ClairS and DeepSomatic. (B) Somatic indel calling performance in recall and precision of ClairS and DeepSomatic, deletions and insertions were filled with stacked bars in different alpha.

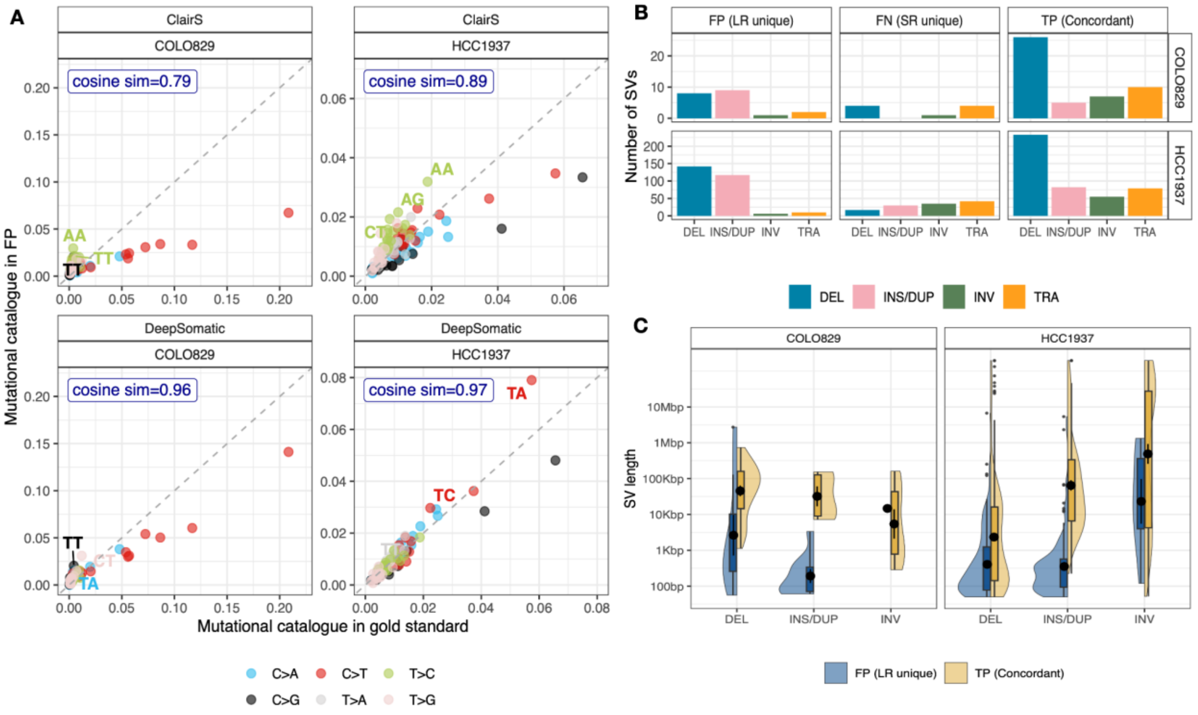

Figure 5: Potential novel events detected by LRS. (A) The comparison of the 96 mutational catalogues between the short-read gold standard and FP from LRS. (B) The number of SVs in four types (DEL: deletion, INS/DUP: insertion/duplication, INV: inversion, TRA: translocation) from FP, FN, and TP. (C) The SV length distribution of FP and TP events in DEL, INS/DUP and INV types, the black dot in each bar indicates the mean value.

References

1. Alexandrov LB, Kim J, Haradhvala NJ, Huang MN, Tian Ng AW, Wu Y, et al.. The repertoire of mutational signatures in human cancer. *Nature*. Springer Science and Business Media LLC; 578:94–1012020;

2. Hanahan D. Hallmarks of cancer: New dimensions. *Cancer Discov*. American Association for Cancer Research (AACR); 12:31–462022;

3. ICGC/TCGA Pan-Cancer Analysis of Whole Genomes Consortium. Pan-cancer analysis of whole genomes. *Nature*. Springer Science and Business Media LLC; 578:82–932020;

4. Yokoyama A, Kakiuchi N, Yoshizato T, Nannya Y, Suzuki H, Takeuchi Y, et al.. Age-related remodelling of oesophageal epithelia by mutated cancer drivers. *Nature*. Springer Science and Business Media LLC; 565:312–72019;

5. Ramarao-Milne P, Kondrashova O, Patch A-M, Nones K, Koufariotis LT, Newell F, et al.. Comparison of actionable events detected in cancer genomes by whole-genome sequencing, in silico whole-exome and mutation panels. *ESMO Open*. 7:1005402022;

6. Lee JK, Sivakumar S, Schrock AB, Madison R, Fabrizio D, Gjoerup O, et al.. Comprehensive pan-cancer genomic landscape of KRAS altered cancers and real-world outcomes in solid tumors. *NPJ Precis Oncol*. Springer Science and Business Media LLC; 6:912022;

7. Chakravarty D, Solit DB. Clinical cancer genomic profiling. *Nat Rev Genet*. Springer Science and Business Media LLC; 22:483–5012021;

8. Dwarshuis N, Kalra D, McDaniel J, Sanio P, Alvarez Jerez P, Jadhav B, et al.. The GIAB genomic stratifications resource for human reference genomes. *Nat Commun*. Springer Science and Business Media LLC; 15:90292024;

9. Ermini L, Driguez P. The application of long-read sequencing to cancer. *Cancers (Basel)*. 2024; doi: 10.3390/cancers16071275.

10. Mahmoud M, Gobet N, Cruz-Dávalos DI, Mounier N, Dessimoz C, Sedlazeck FJ. Structural variant calling: the long and the short of it. *Genome Biol*. Springer Science and Business Media LLC; 20:2462019;

11. Rhoads A, Au KF. PacBio Sequencing and its applications. *Genomics Proteomics Bioinformatics*. Oxford University Press (OUP); 13:278–892015;

12. Wang Y, Zhao Y, Bollas A, Wang Y, Au KF. Nanopore sequencing technology, bioinformatics and applications. *Nat Biotechnol*. Springer Science and Business Media LLC; 39:1348–652021;

13. Huddleston J, Ranade S, Malig M, Antonacci F, Chaisson M, Hon L, et al.. Reconstructing complex regions of genomes using long-read sequencing technology. *Genome Res*. Cold Spring Harbor Laboratory; 24:688–962014;

14. Nurk S, Koren S, Rhie A, Rautiainen M, Bzikadze AV, Mikheenko A, et al.. The complete sequence of a human genome. *Science*. American Association for the Advancement of Science (AAAS); 376:44–532022;

15. Kuleshov V, Xie D, Chen R, Pushkarev D, Ma Z, Blauwkamp T, et al.. Whole-genome haplotyping using long reads and statistical methods. *Nat Biotechnol*. Springer Science and Business Media LLC; 32:261–62014;

16. Sakamoto Y, Sereewattanawoot S, Suzuki A. A new era of long-read sequencing for cancer genomics. *J Hum Genet*. Springer Science and Business Media LLC; 65:3–102020;

17. Nattestad M, Goodwin S, Ng K, Baslan T, Sedlazeck FJ, Rescheneder P, et al.. Complex rearrangements and oncogene amplifications revealed by long-read DNA and RNA sequencing of a breast cancer cell line. *Genome Res*. Cold Spring Harbor Laboratory; 28:1126–352018;

18. Cui X, Lin Q, Chen M, Wang Y, Wang Y, Wang Y, et al.. Long-read sequencing unveils novel somatic variants and methylation patterns in the genetic information system of early lung cancer. *Comput Biol Med*. Elsevier BV; 171:1081742024;

19. Xu L, Wang X, Lu X, Liang F, Liu Z, Zhang H, et al.. Long-read sequencing identifies novel structural variations in colorectal cancer. *PLoS Genet*. 19:e10105142023;

20. O’Neill K, Pleasance E, Fan J, Akbari V, Chang G, Dixon K, et al.. Long-read sequencing of an advanced cancer cohort resolves rearrangements, unravels haplotypes, and reveals methylation landscapes. *Cell Genom*. Elsevier BV; 4:1006742024;

21. Brändl B, Steiger M, Kubelt C, Rohrandt C, Zhu Z, Evers M, et al.. Rapid brain tumor classification from sparse epigenomic data. *Nat Med*. Nature Publishing Group; 31:840–82025;

22. Patel A, Göbel K, Ille S, Hinz F, Schoebe N, Bogumil H, et al.. Prospective, multicenter validation of a platform for rapid molecular profiling of central nervous system tumors. *Nat Med*. Nature Publishing Group; :1–11 2025;

23. Amarasinghe SL, Ritchie ME, Gouil Q. Long-read-tools.org: An interactive catalogue of analysis methods for long-read sequencing data. *Gigascience*. Oxford University Press (OUP); 2021; doi: 10.1093/gigascience/giab003.

24. Jain M, Koren S, Miga KH, Quick J, Rand AC, Sasani TA, et al.. Nanopore sequencing and assembly of a human genome with ultra-long reads. *Nat Biotechnol*. 36:338–452018;

25. Sedlazeck FJ, Lee H, Darby CA, Schatz MC. Piercing the dark matter: bioinformatics of long-range sequencing and mapping. *Nat Rev Genet*. Springer Science and Business Media LLC; 19:329–462018;

26. LoTempio J, Delot E, Vilain E. Benchmarking long-read genome sequence alignment tools for human genomics applications. *PeerJ*. 11:e165152023;

27. Liu L, Zhang J, Wood S, Newell F, Leonard C, Koufariotis LT, et al.. Performance of somatic structural variant calling in lung cancer using Oxford Nanopore sequencing technology. *BMC Genomics*. Springer Science and Business Media LLC; 25:8982024;

28. Sigurpalsdottir BD, Stefansson OA, Holley G, Beyter D, Zink F, Hardarson MÞ, et al.. A comparison of methods for detecting DNA methylation from long-read sequencing of human genomes. *Genome Biol*. Springer Science and Business Media LLC; 25:692024;

29. Dong X, Du MRM, Gouil Q, Tian L, Jabbari JS, Bowden R, et al.. Benchmarking long-read RNA-sequencing analysis tools using in silico mixtures. *Nat Methods*. Springer Science and Business Media LLC; 20:1810–212023;

30. Liu Y, Rosikiewicz W, Pan Z, Jillette N, Wang P, Taghbalout A, et al.. DNA methylation-calling tools for Oxford Nanopore sequencing: a survey and human epigenome-wide evaluation. *Genome Biol*. 22:2952021;

31. Park J, Cook DE, Chang P-C, Kolesnikov A, Brambrink L, Mier JC, et al.. DeepSomatic: Accurate somatic small variant discovery for multiple sequencing technologies. bioRxivorg.

32. Zheng Z, Su J, Chen L, Lee Y-L, Lam T-W, Luo R. ClairS: a deep-learning method for long-read somatic small variant calling. bioRxiv.

33. Keskus A, Bryant A, Ahmad T, Yoo B, Aganezov S, Goretsky A, et al.. Severus: accurate detection and characterization of somatic structural variation in tumor genomes using long reads. *medRxiv*. :2024.03.22.243047562024;

34. Elrick H, Sauer CM, Espejo Valle-Inclan J, Trevers K, Tanguy M, Zumalave S, et al.. SAVANA: reliable analysis of somatic structural variants and copy number aberrations in clinical samples using long-read sequencing. bioRxiv.

35. Griffith M, Miller CA, Griffith OL, Krysiak K, Skidmore ZL, Ramu A, et al.. Optimizing cancer genome sequencing and analysis. *Cell Syst*. Elsevier BV; 1:210–232015;

36. Xiao W, Ren L, Chen Z, Fang LT, Zhao Y, Lack J, et al.. Toward best practice in cancer mutation detection with whole-genome and whole-exome sequencing. *Nat Biotechnol*.

39:1141–502021;

37. Yu T, Huang Q, Zhao X, Zhang S, Zhang Q, Fan X, et al.. Tumour purity as an underlying key factor in tumour mutation detection in colorectal cancer. *Clin Transl Med*. Wiley; 13:e12522023;

38. Haider S, Tyekucheva S, Prandi D, Fox NS, Ahn J, Xu AW, et al.. Systematic assessment of tumor purity and its clinical implications. *JCO Precis Oncol*. American Society of Clinical Oncology; 2020; doi: 10.1200/PO.20.00016.

39. Aran D, Sirota M, Butte AJ. Systematic pan-cancer analysis of tumour purity. *Nat Commun*. Springer Science and Business Media LLC; 6:89712015;

40. Mwenifumbo JC, Marra MA. Cancer genome-sequencing study design. *Nat Rev Genet*. Springer Science and Business Media LLC; 14:321–322013;

41. Sendorek DH, Caloian C, Ellrott K, Bare JC, Yamaguchi TN, Ewing AD, et al.. Germline contamination and leakage in whole genome somatic single nucleotide variant detection. *BMC Bioinformatics*. Springer Science and Business Media LLC; 19:282018;

42. Chen S, Francioli LC, Goodrich JK, Collins RL, Kanai M, Wang Q, et al.. A genomic mutational constraint map using variation in 76,156 human genomes. *Nature*. 625:92–1002024;

43. Espejo Valle-Inclan J, Besselink NJM, de Bruijn E, Cameron DL, Ebler J, Kutzera J, et al.. A multi-platform reference for somatic structural variation detection. *Cell Genom*. Elsevier BV; 2:1001392022;

44. Yuen ZW-S, Srivastava A, Daniel R, McNevin D, Jack C, Eyras E. Systematic benchmarking of tools for CpG methylation detection from nanopore sequencing. *Nat Commun*. Springer Science and Business Media LLC; 12:34382021;

45. Pardo-Palacios FJ, Wang D, Reese F, Diekhans M, Carbonell-Sala S, Williams B, et al.. Systematic assessment of long-read RNA-seq methods for transcript identification and quantification. *Nat Methods*. Springer Science and Business Media LLC; 21:1349–632024;

46. Alioto TS, Buchhalter I, Derdak S, Hutter B, Eldridge MD, Hovig E, et al.. A comprehensive assessment of somatic mutation detection in cancer using whole-genome sequencing. *Nat Commun*. Springer Science and Business Media LLC; 6:100012015;

47. Poplin R, Chang P-C, Alexander D, Schwartz S, Colthurst T, Ku A, et al.. A universal SNP and small-indel variant caller using deep neural networks. *Nat Biotechnol*. Springer Science and Business Media LLC; 36:983–72018;

48. Le MK, Qin Q, Li H. Long-range somatic structural variation calling from matched tumor-normal co-assembly graphs. bioRxiv.

49. De La Cerda GY, Landis JB, Eifler E, Hernandez AI, Li F-W, Zhang J, et al.. Balancing read length and sequencing depth: Optimizing Nanopore long-read sequencing for monocots with an emphasis on the Liliales. *Appl Plant Sci*. 11:e115242023;

50. Kosugi S, Terao C. Comparative evaluation of SNVs, indels, and structural variations detected with short- and long-read sequencing data. *Hum Genome Var*. Nature Publishing Group; 11:182024;

51. Choo Z-N, Behr JM, Deshpande A, Hadi K, Yao X, Tian H, et al.. Most large structural variants in cancer genomes can be detected without long reads. *Nat Genet*. Springer

Science and Business Media LLC; 55:2139–482023;

52. Kolmogorov M, Billingsley KJ, Mastoras M, Meredith M, Monlong J, Lorig-Roach R, et al.. Scalable Nanopore sequencing of human genomes provides a comprehensive view of haplotype-resolved variation and methylation. *Nat Methods*. 20:1483–922023;

53. Nyaga DM, Tsai P, Gebbie C, Phua HH, Yap P, Le Quesne Stabej P, et al.. Benchmarking nanopore sequencing and rapid genomics feasibility: validation at a quaternary hospital in New Zealand. *NPJ Genom Med*. Springer Science and Business Media LLC; 9:572024;

54. Olson ND, Wagner J, Dwarshuis N, Miga KH, Sedlazeck FJ, Salit M, et al.. Variant calling and benchmarking in an era of complete human genome sequences. *Nat Rev Genet*. Springer Science and Business Media LLC; 24:464–832023;

55. Paulin LF, Fan J, O'Neill K, Pleasance E, Porter VL, Jones SJM, et al.. The benefit of a complete reference genome for cancer structural variant analysis. *medRxiv*. :2024.03.15.243043692024;

56. Ten Berk de Boer E, Ameur A, Bunikis I, Ek M, Stattin E-L, Feuk L, et al.. Long-read sequencing and optical mapping generates near T2T assemblies that resolves a centromeric translocation. *Sci Rep*. 14:90002024;

57. Li H, Bloom JM, Farjoun Y, Fleharty M, Gauthier L, Neale B, et al.. A synthetic-diploid benchmark for accurate variant-calling evaluation. *Nat Methods*. Nature Publishing Group; 15:595–72018;

58. Yang C, Chu J, Warren RL, Birol I. NanoSim: nanopore sequence read simulator based on statistical characterization. *Gigascience*. Oxford Academic; 6:1–62017;

59. Ono Y, Asai K, Hamada M. PBSIM2: a simulator for long-read sequencers with a novel generative model of quality scores. *Bioinformatics*. Oxford University Press (OUP); 37:589–952021;

60. Craig DW, Nasser S, Corbett R, Chan SK, Murray L, Legendre C, et al.. A somatic reference standard for cancer genome sequencing. *Sci Rep*. Springer Science and Business Media LLC; 6:246072016;

61. Ewing AD, Houlahan KE, Hu Y, Ellrott K, Caloian C, Yamaguchi TN, et al.. Combining tumor genome simulation with crowdsourcing to benchmark somatic single-nucleotide-variant detection. *Nat Methods*. Springer Science and Business Media LLC; 12:623–302015;

62. English AC, Dolzhenko E, Ziaei Jam H, McKenzie SK, Olson ND, De Coster W, et al.. Analysis and benchmarking of small and large genomic variants across tandem repeats. *Nat Biotechnol*. Nature Publishing Group; :1–12 2024;

63. . Dorado: Oxford nanopore's basecaller. Github;

64. Li H, Handsaker B, Wysoker A, Fennell T, Ruan J, Homer N, et al.. The Sequence Alignment/Map format and SAMtools. *Bioinformatics*. Oxford University Press (OUP); 25:2078–92009;

65. Li H. New strategies to improve minimap2 alignment accuracy. *Bioinformatics*. Oxford University Press (OUP); 37:4572–42021;

66. Tarasov A, Vilella AJ, Cuppen E, Nijman IJ, Prins P. Sambamba: fast processing of NGS alignment formats. *Bioinformatics*. Oxford University Press (OUP); 31:2032–42015;

67. Pedersen BS, Quinlan AR. Mosdepth: quick coverage calculation for genomes and exomes. *Bioinformatics*. Oxford University Press (OUP); 34:867–82018;

68. Danecek P, Bonfield JK, Liddle J, Marshall J, Ohan V, Pollard MO, et al.. Twelve years of SAMtools and BCFtools. *Gigascience*. Oxford University Press (OUP); 2021; doi: 10.1093/gigascience/giab008.

69. Rausch T, Zichner T, Schlattl A, Stütz AM, Benes V, Korbel JO. DELLY: structural variant discovery by integrated paired-end and split-read analysis. *Bioinformatics*. Oxford University Press (OUP); 28:i333–92012;

70. Shiraishi Y, Koya J, Chiba K, Okada A, Arai Y, Saito Y, et al.. Precise characterization of somatic complex structural variations from tumor/control paired long-read sequencing data with nanomonsv. *Nucleic Acids Res*. Oxford University Press (OUP); 51:e742023;

71. Martin M, Ebert P, Marschall T. Read-based phasing and analysis of phased variants with WhatsHap. *Methods Mol Biol*. 2590:127–382023;

72. Van der Auwera GA, Carneiro MO, Hartl C, Poplin R, del Angel G, Levy-Moonshine A, et al.. From FastQ Data to High-Confidence Variant Calls: The Genome Analysis Toolkit Best Practices Pipeline. *Current Protocols in Bioinformatics*. 43:11.10.1-11.10.332013;

73. Kassahn KS, Holmes O, Nones K, Patch A-M, Miller DK, Christ AN, et al.. Somatic point mutation calling in low cellularity tumors. *PLoS One*. Public Library of Science (PLoS); 8:e743802013;

74. Cameron DL, Baber J, Shale C, Valle-Inclan JE, Besselink N, van Hoeck A, et al.. GRIDSS2: comprehensive characterisation of somatic structural variation using single breakend variants and structural variant phasing. *Genome Biol*. Springer Science and Business Media LLC; 22:2022021;

75. Layer RM, Chiang C, Quinlan AR, Hall IM. LUMPY: a probabilistic framework for structural variant discovery. *Genome Biol*. Springer Nature; 15:R842014;

76. Kirsche M, Prabhu G, Sherman R, Ni B, Battle A, Aganezov S, et al.. Jasmine and Iris: population-scale structural variant comparison and analysis. *Nat Methods*. Springer Science and Business Media LLC; 20:408–172023;

77. Aryee MJ, Jaffe AE, Corrada-Bravo H, Ladd-Acosta C, Feinberg AP, Hansen KD, et al.. Minfi: a flexible and comprehensive Bioconductor package for the analysis of Infinium DNA methylation microarrays. *Bioinformatics*. Oxford University Press (OUP); 30:1363–92014;

78. Lawrence M, Huber W, Pagès H, Aboyoun P, Carlson M, Gentleman R, et al.. Software for computing and annotating genomic ranges. *PLoS Comput Biol*. Public Library of Science (PLoS); 9:e10031182013;

79. Lawrence M, Gentleman R, Carey V. rtracklayer: an R package for interfacing with genome browsers. *Bioinformatics*. Oxford University Press (OUP); 25:1841–22009;

80. Nassar LR, Barber GP, Benet-Pagès A, Casper J, Clawson H, Diekhans M, et al.. The UCSC Genome Browser database: 2023 update. *Nucleic Acids Res*. Oxford University Press (OUP); 51:D1188–952023;

81. Obenchain V, Lawrence M, Carey V, Gogarten S, Shannon P, Morgan M. VariantAnnotation: a Bioconductor package for exploration and annotation of genetic variants. *Bioinformatics*. Oxford University Press (OUP); 30:2076–82014;

82. Perez G, Barber GP, Benet-Pages A, Casper J, Clawson H, Diekhans M, et al.. The UCSC Genome Browser database: 2025 update. *Nucleic Acids Res*. Oxford University Press (OUP); 53:D1243–92025;

83. Rosenthal R, McGranahan N, Herrero J, Taylor BS, Swanton C. DeconstructSigs: delineating mutational processes in single tumors distinguishes DNA repair deficiencies and patterns of carcinoma evolution. *Genome Biol*. Springer Science and Business Media LLC; 17:312016;

84. Wickham H, Averick M, Bryan J, Chang W, McGowan LD, François R, et al.. Welcome to the tidyverse. Journal of Open Source Software.

85. Wickham H. ggplot2: Elegant Graphics for Data Analysis. Springer-Verlag New York;

86. van den Brand T. ggh4x: Hacks for “ggplot2.”

Click here to access/download
Supplementary Material
Supplementary Tables.pdf

Click here to access/download
Supplementary Material
Supplementary figures_revision_Marked.docx

Click here to access/download
Supplementary Material
Supplementary figures_revision_Clean.docx
