# Supplementary material for: Cancer genome standards for long-read sequencing using cancer cell line mixtures
Source: Gigascience. 2026 Apr 3;15:giag037. doi: 10.1093/gigascience/giag037 (PMC13137868; doi:10.1093/gigascience/giag037)
Supplement: giag037_Authors_Response_To_Reviewer_Comments_original_submission [file giag037_authors_response_to_reviewer_comments_original_submission.pdf]

Author's Response To Reviewer Comments

Thank you for the time and effort you spent on this manuscript. We would like to thank both referees for their thoughtful, in-depth reviews which have helped improve our manuscript. Based on their comments, we have revised the manuscript. Changes within the manuscript are highlighted as tracked changes. Please see below for a point-by-point response to the reviewers' comments.

Reviewer #1

Reviewer Comment:
The study measured how somatic variant classification with long-read sequencing (LRS) technology was affected by data quality: tumor sample purity and read depth. ……….., public release of the variable purity and sequencing depth dataset would enable further research into how data quality impacts somatic variant calling.

Response: We thank Reviewer 1 for the summary and highlighting the impact of our research. We also agree that the variable tumour dataset will enable further research. Therefore, we have deposited the BAM files of these data in the EGA under accession number EGAD00001015628 under study EGAS00001008107. The POD5 files are available on request. For the variable sequencing depth, these data were created from the sequence data which has been deposited in the EGA.

Reviewer Comment:
Strengths
1. The impact of the work included 1) informing data quality standards for somatic variant analysis, 2) hypothesizing somatic variants distinct to LRS methods and missed by SRS methods, and 3) a nuanced evaluation of various somatic variant calling tools in different data quality scenarios.
2. The analysis was novel compared to previous germline variant LRS benchmarks since, here, the experiments evaluated data quality aspects distinct to cancer datasets: tumor purity and sequencing depth of paired tumor and normal samples.
3. Interpretation of experimental results was mostly correct. And, the discussion section accurately summarized key findings regarding the effect of tumor purity and sequencing depth on somatic variant calling.

Response: We appreciate these positive comments and highlighting the novelty of the work.

Reviewer Comment:
Weakness
1. The lack of analysis for overlapping true positives or false positives across methods was a missed opportunity to understand the potential impact of ensemble methods. Albeit, an analysis of overlapping calls is slightly out of scope of this work. Therefore, I do not see overlap analysis as a necessary revision.

Response: We expect that an ensemble callset derived from ClairS and DeepSomatic to have higher precision as events detected by both methods are less likely to be false positives. Meanwhile, this comes at the cost of lower recall, since some true positive events may only be captured by a single method. To test this, we performed an inspection of SNV that were called by ClairS and DeepSomatic using samples from our tumour purity experiments.
Changes to manuscript: The results of this overlap analysis are provided in a new Supplementary table (See Supplementary Tables) and we included new text in Results section 2.2 (Page 6):
“The majority of the SNVs were detected by both approaches, with 90% of SNV being detected by ClairS and DeepSomatic for the COLO829 non-diluted sample and 87% for HCC1937 (Supplementary Table S2). The union of the two approaches achieved higher recall at the cost of introducing more FP events.”

Reviewer Comment:
2. The argument for filtering ClairS by a singular variant statistic should be cautioned by the implied decrease in recall when applying filters. From the results figures, it's unclear whether any threshold could

be applied for a singular variant statistic (e.g., sequencing depth, mapping quality, or variant allele frequency) without a significant reduction in recall. A safer argument is to filter by a combination of these statistics.

Response: We agree with the referee regarding the statement of using a combination of statistics to apply filters.
Changes to manuscript: We have revised the statement in the Results section 2.2 (Page 6) to improve clarity. The updated text now reads:
"The different patterns observed in allele frequency, read depth, and quality score distributions between TP and FP events suggest that integrating these measures could serve as an efficient filter to reduce FP calls."

Reviewer Comment:
Minor comments
Interpretation of SF16 results seems incorrect. The majority of LRS FPs overlap with high/low GC content regions for most methods, not low mappability regions. This argument is mentioned on Pg 9, sec 2.4, paragraph 2, lines 4-5.

Response: Thank you for the correction.
Changes to manuscript: We have revised the text to clarify the interpretation. The corrected sentence now reads:
"In support of this, we found that a substantial fraction of the LR-specific SVs are located at genomic regions of extreme GC content (GC%<30 or >55) or within low mappability for short reads".

Reviewer Comment:
Regarding the Figure 5A analysis, I do not understand how SBS vectors are set up for cosine similarity measurement. Please make it clear how SBS vectors are set-up/aggregated from variants. Also, a baseline reference of the expected cosine similarity at random would make this result more impactful and interpretable.

Response: SBS vectors refers to the single base substitution (SBS) profile categorised by the base change type (C>A, C>G, C>T, T>A, T>C, and T>G) within a trinucleotide context (the immediate 5' and 3' neighbours). This results in a 96-dimensional vector (SBS-96), which is the standard framework for describing mutational signatures in cancer (as described in [1]). These SBS signatures can provide some insight about how tumours develop. For example, in the COLO829 cell line, which is derived from a sun exposed melanoma sample, a high proportion of the SBS somatic mutations are linked to signatures associated with UV exposure (SBS7). Therefore, in Figure 5A, we tested whether the mutational profiles of LR variants classified as FP SNVs resemble those in short-read "gold standard" – and thus more likely to be true events.
Cosine similarity is a common approach to compare signatures (eg. [2,3]) and was used as it is insensitive to the absolute scale of counts, the shape of mutational spectrum across 96 channels is what matters. The high cosine similarity (0.79-0.97) we observe indicates that the FP calls recapitulate the same mutational patterns as the gold-standard set, suggesting that many of these calls may indeed reflect real biological signatures rather than random sequencing artefacts. This is because a cosine similarity score >0.85 suggests the sample's mutational profile strongly matches the reference, 0.6-0.8 represents partial similarity and <0.5 score suggests little resemblance. Our random simulation based on random sampling 96-SBS channels showed a background similarity of approximately 0.35.
Changes to manuscript: We agree that Figure 5A is rather a complicated figure and realised the main reason for the complexity is that we missed the method description for these results. We apologise for this omission and have now included a new section in the Methods (Section 4.13) which reads:
"4.13 Mutational signatures analysis
All SNV somatic mutations were converted into a 96-channel mutational profile (SBS-96 vector) and were subsequently decomposed into contributions of COSMIC reference catalogue (v2.0, GRCh38) using the R package deconstructSigs (v1.8.0) [83]. To evaluate whether LR specific variants, classified as false positives, might represent genuine mutational events, we compared their mutational spectra with the short-read gold-standard set. Specifically, a cosine similarity was computed between the two profiles."

Reviewer Comment:

The results of supplementary figure (SF) 3 seemed unrealistic to me. Specifically, I am surprised that high/low methylation always gets reversed as a function of purity. Please provide some intuition for why this result is (un)-expected, and double-check this result.

Response: The methylation frequency distribution plot (Supplementary Figure S3B) only shows the CpG sites with differential methylation frequency between tumour and blood lymphocytes cell lines. These sites were identified from EPIC Array data by extracting sites with tumour-specific methylation status where the beta values (methylation frequency) are ≥0.7 in tumour and ≤0.3 in BL, or ≤0.3 in tumour and ≥0.7 in BL. Consequently, in the mixing experiments, highly methylated sites show progressive decreases in methylation frequency, while demethylated sites display increases as the proportion of BL DNA rises. To validate that our cell line mixing recapitulated the expected tumour purity levels, we used both variant allele frequency (VAF) distributions and methylation frequency curves as orthogonal measures.
Changes to manuscript: We have included additional text in the Supplementary Figure 3 legend:
"Only the CpG sites with differential methylation frequency between the 100% tumour and blood lymphocyte cell lines are included in the plot."
We have also amended the results text to clarify the findings:
"The shifted distributions of variant mutation allele frequency (VAF) for SNVs (Supplementary Figure S3A) and methylation frequency at CpG sites that are differentially methylated between the tumour and normal cell line (Supplementary Figure S3B) supports the serial dilutions of tumour DNA by normal cell DNA."
We have also clarified the approach in a revised Methods section (4.10), which now reads:
"The methylation data was also used to confirm the tumour purity levels within the serial diluted samples. Specifically, differentially methylated CpG sites between the tumour and blood lymphocyte cell lines were determined as probes with Beta values ≥0.7 in tumour and ≤0.3 in BL, or ≤0.3 in tumour and ≥0.7 in BL. The distribution of these CpG sites was then assessed in the samples with different tumour purities for COLO829 and HCC1937."

Reviewer Comment:
When reading the paper top-down, it is confusing why the definitions of FP, TP, and FN were switched to LRS variants, overlapping variants and SRS variants in SF7. The analysis of SF7 preceded the evidence of LRS FPs being missed by the SRS standard shown later in Fig 5. Overall, the foreshadowing of LRS FPs being missed calls made me skeptical until there was evidence supporting this hypothesis later in the text.

Response: We hold a premise that long reads can improve variant calling in genomic regions that are difficult for short reads to align, especially for structural variation. For this reason, we emphasised this point in the SV benchmarking and originally referred to FP as LR novel and FN as SR specific events in Supplementary Figure S7 and S10. However, as pointed out by the reviewer, we acknowledge that introducing this terminology before Figure 5 may have caused confusion.
Changes to manuscript: We have now updated Supplementary Figure S7 and S10 to use the standard FP, FN and TP labels for consistency.

Reviewer Comment:
The supplementary figure 8 legend is incorrect. Column and row descriptions do not match the figure. And, what does DP stand for?

Response: Thank you for bringing this error to our attention.
Changes to manuscript: The column and row descriptions in the Supplementary Figure S8 legend have been corrected, and we have clarified that DP refers to the read depth in the tumour sample. The figure legend now reads:
"Supplementary Figure S8. Variant allele frequency (VAF), read depth in tumour (DP) and quality score (QUAL) of FP and TP SNVs calls from ClairS and DeepSomatic in COLO829 and HCC1937 samples with nine sequencing depth combinations. Panels are arranged with COLO829 on the left and HCC1937 on the right, and ClairS on the top and DeepSomatic on the bottom. Within each panel, results are further separated by sequencing depth combinations ranging from 15x -15x to 60x - 60x (tumour - normal). FP calls are shown in blue and TP calls in yellow."

Reviewer Comment:
A direct quantification, such as the difference of mean recalls across methods, is missing between SRS

standard and secondary callset.
Response: Thank you for this suggestion. In the Results section, we have reported mean recall and precision values for both SNV and indel calling to the text.
The text now reads:
Changes to manuscript: “In terms of recall, DeepSomatic had a higher mean recall at all tumour purity levels except the sample with 100% tumour purity compared to ClairS for COLO829 (ClairS 81.27%, DeepSomatic 86.39%), however, ClairS had a higher mean recall than DeepSomatic in HCC1937 in samples with lower tumour purity (<50%) (ClairS 81.43%, DeepSomatic 77.02%).”

Reviewer Comment:
The abstract argues that LRS better aligns to "complex" regions (abstract, line 2). The intro discusses how SRS is bad in low complexity regions (introduction, lines 7&8) … later in the intro, LRS is again discussed as effectively "spanning complex genomic regions" (introduction, line 11). I believe the argument attempting to be made is that LRS better aligns to low complexity regions than SRS. Please make this argument consistent and clear.

Response: Thank you for pointing out this inconsistency. Our intended argument is that longer reads provide improved alignment and variant detection in low complexity regions, which usually contain highly repetitive sequences.
Changes to manuscript: We have revised the wording in the abstract and introduction to ensure consistency and clarity. The updated wording now consistently refers to “low complexity regions” or “highly repetitive regions” rather than “complex regions”.

Reviewer Comment:
For analysis of SF10 in section 2.3, the consistently high precision of nanomonsv seems worth mentioning. This is mentioned in the discussion, but I think it should be mentioned in the results section, too.
Changes to manuscript: We have included new text highlighting the consistently high precision of nanomonsv in the Results section (Section 2.3). The revised text reads:
“Nanomonsv consistently demonstrated the highest precision across tumour purities, underscoring the importance of incorporating a reference panel to achieve accurate variant detection.”

Reviewer Comment:
It would be helpful to indicate which stains are telomere and centromere regions in supplementary figure 11-14 keys.

Response: In the chromosome ideograms, centromeres are coloured in red (acen), but there is no specific code for telomeres as they are the natural ends of chromosomes.
Changes to manuscript: We have revised the key in Supplementary Figures S11-14 to include details of the giemsa staining code and have revised the Figure legends accordingly.
In addition, we have expanded the information for the rainfall plot and chromosome diagram band colouring in the Methods section 4.12 to provide further clarification on the rainfall plots and chromosome ideogram tracks. The new text reads:
“The genome-wide distribution of TP, FP and FN SNVs was visualised using rainfall plots which were aligned with chromosome ideograms annotated with cytogenetic banding patterns (gieStain) obtained from the UCSC genome browser”

Reviewer Comment:
"N50 is" -> "N50 was" (results, paragraph 1, last line)
Make capitalization consistent for the sub-figure character labels in supplementary figure 1.
At section 2.2, paragraph 5, line 2 change plural "indels" to singular "indel"
Discussion, p14, para 8, line 7, "on" -> "one"
Conf of interest, P20, l1, "Nanopre" -> "Nanopo

Response: These have been fixed. Thank you for finding the errors.

Reviewer #2:

Reviewer Comment: This study assesses the accuracy of long-read sequencing with Oxford Nanopore Technology (ONT) for detecting somatic variants in tumor cell line mixtures, offering valuable insights for optimizing cancer genomic analyses, particularly regarding sequencing depth, tumor purity, and comparisons with short-read technologies. Overall, the manuscript is well-written, clearly structured, and presents significant findings in a highly relevant area of research.

Response: We thank Reviewer 2 for these positive comments and recognition of the quality and value of our research.

Reviewer Comment:
However, the code repository for this study (https://github.com/bakeronit/nanopore_celllines_benchmark) is currently unavailable. It is unclear whether the repository is private or has been renamed. Considering that GigaScience strongly emphasizes reproducibility, the inability to access and verify the repository significantly limits this review.

Response: Thank you for bringing this to our attention. We apologize for the inconvenience caused by the repository being private at the time of review. We did upload the whole github repository as a zipped package to GigaDB FTP server with all intermediate data prior to the review stage. The github repository was initially kept private to ensure that the full code and workflow would be presented in its proper scientific context along with the publication of this manuscript. We fully recognize the importance of transparency and reproducibility. To further facilitate reproducibility, the GitHub repository has now been made publicly accessible, with branch rev has been created to reflect all updates and changes made during our revision. This repository can now be accessed at:https://github.com/bakeronit/nanopore_celllines_benchmark. We hope this resolves the concern and demonstrates our commitment to open and reproducible research.

Reviewer Comment:
The manuscript mentions the use of downsampling to synthetically reduce coverage depth, but upsampling-based simulations were not explored. I strongly recommend including synthetic datasets, as they can substantially enhance both experimental design and result validation. For example: PBSIM2 for PacBio and ONT (https://github.com/yukiteruono/pbsim2), NanoSim for ONT (https://github.com/bcgsc/NanoSim). Such simulations offer several advantages: Validation of the reconstruction methodology; Full control over somatic variants (gold standard); Tunable sequencing coverage; Exploration of extreme scenarios; Cross-validation of real data (false positives/negatives); Systematic technology comparisons; Benchmarking against real datasets. Similarly, short-read simulations could be performed using ART (https://www.niehs.nih.gov/research/resources/software/biostatistics/art) or art_modern (https://github.com/YU-Zhejian/art_modern).

Response: We appreciate this suggestion and did spend some time reviewing NanoSim and pbsim2 as they seem like useful approaches. We tried to use nanosim to simulate ONT reads, however, even with 1% of reads sampled, the model training took more than 48 hours with 30 CPUs and 50Gb RAM. Furthermore, we feel that these approaches are not a good fit for our study because they are unable to capture all sequence artefacts and account for rearranged cancer genomes. We deliberately chose to generate sequence data using in vitro mixing of DNA samples. Although we agree that our DNA mix simulations do not cover all extreme scenarios, we believe that as we sequenced each DNA mixture, they reflect the expected performance of the technology over the majority of common strategies.
While upsampling data can be useful for controlled experiments, the premise of generating new ONT data based on profiles learned from a subset of data is less than ideal in our case. Therefore, we elected to sequence samples quite deeply (>60x) then used downsampling of these data to determine whether less data would still allow variant detection. Our downsampling approach was a random process of generating less reads from a sequencer, with every read from the real genome from tumour.
A challenge we are trying to address in this study is the lack of “gold standard” sequence variant data. In particular there is a lack of perfect gold standards for tumour samples to validate variant detection pipelines. Therefore, we hope the data generated in this manuscript will be a useful resource to others.
Changes to manuscript: We have included new text in discussion, acknowledge the potential use of read simulators to assess the extreme scenarios for variant calling. Now reads:
“Previously researchers have utilised the synthetic mixtures of two fully homozygous human cell lines to

mock somatic mutation, and apply read simulators such as NanoSim or pbsim2 to upsample data for validation of variant detection. While this is a good strategy to explore variant calling performance under extreme scenarios, differences in variation calling performance were found when using the real tumour cell line, which underscores the heterogeneous nature of cancer samples."

Reviewer Comment:
Sequencing technology strongly impacts the interpretation of long-read studies, as differences in error profiles, read length, required depth, and compatible tools directly influence sensitivity, precision, and the types of variants that can be reliably detected. This study exclusively reports ONT-based results, and some conclusions may not generalize to other long-read platforms. It would be more informative to explicitly mention the sequencing technology in the abstract and possibly in the article title.

Response: We acknowledge that our analysis and conclusions are most directly applicable to ONT and may not generalize fully to other long-read technologies such as PacBio HiFi.
Changes to manuscript: To make this scope clear to readers, we have revised the abstract to explicitly mention that the results are based on Oxford Nanopore sequencing.

Reviewer Comment:
The use of GRCh38 is suboptimal when compared to CHM13-T2T, which resolves previously unresolved repetitive, telomeric, and centromeric regions-areas that the study itself identifies as relevant to accuracy. Incorporating CHM13-T2T in the current analysis could significantly strengthen the study, and would also make Supplementary Figures 11-14 more informative.

Response: This is an excellent point. In the original manuscript, we had already raised this point in discussion referencing recent publications that demonstrate the value of CHM13-T2T eg.,[4]. However, GRCh38 remains the current most widely adopted reference in cancer genomics research and clinical use, because of its extensive functional and clinical annotations, integration with large-scale resources (e.g. gnomAD, TCGA), and compatibility with the majority of computational approaches. While we appreciate the value of CHM13-T2T assembly, our main goal for this study is to evaluate the LRS in the current most common clinical cancer genomics setting, which is based on GRCh38.
Even so, we have now conducted new analysis using the COLO829 and HCC1937 cell lines, where we evaluate genomic stratification of calls using CHM13-T2T assembly. We fully agree that the CHM13-T2T assembly represents the most complete human genome assembly to date and provides advantages over GRCh38 for variant calling, particularly in resolving repetitive, telomeric and centromeric regions. This is reflected in what we see in the reduced overall sequence divergence between aligned reads and the reference, as well as in the higher mapping rates observed when aligning to CHM13-T2T compared with GRCh38.
Changes to manuscript: We have included a new Supplementary Table S1 (See Supplementary Tables)

Meanwhile, we refer this supplementary table in the results section and provide rationale for the reason to use GRCh38 in our analysis.
"We assessed impact of alignment using different human reference assemblies, CHM13-T2T and GRCh38. Slight improvements were observed using the CHM13-T2T reference with lower sequence divergence between reads and reference sequences and higher mapping rates (Supplementary Table S1). However, in our study we used the GRCh38 assembly due to the comprehensive annotations and maximised compatibility with current tools and our short-read gold standard."

Reviewer Comment:
Including metrics that better capture imbalanced variant detection performance, such as AUROC and AUPRC, would provide a more comprehensive assessment of the methods.

Response: Great suggestion.
Changes to manuscript: We have incorporated AUPRC curves for indel and SV calling in the supplementary figure S4 alongside the AUPRC plot for SNV detection showing the trade-off between precision and recall across tumour purity.

Reviewer Comment:

Minor Comments
In the sentence: "Two approaches were used for somatic SNVs and indels detection from long-read data. ClairS [32] and DeepSomatic [31]." - consider replacing "." with a ":".
Changes to manuscript: We have changed the paper as suggested. This reads better after replacing “.” with “:”.

Reference

1. Alexandrov LB, Kim J, Haradhvala NJ, Huang MN, Tian Ng AW, Wu Y, et al.. The repertoire of mutational signatures in human cancer. Nature. Springer Science and Business Media LLC; 578:94–1012020;
2. Medo M, Ng CKY, Medová M. A comprehensive comparison of tools for fitting mutational signatures. Nat Commun. Springer Science and Business Media LLC; 15:94672024;
3. Jiang N, Wu Y, Rozen SG. Benchmarking 13 tools for mutational signature attribution, including a new and improved algorithm. Brief Bioinform. Oxford University Press (OUP); 26:bbaf0422024;
4. Paulin LF, Fan J, O’Neill K, Pleasance E, Porter VL, Jones SJM, et al.. Closing the gaps, and improving somatic structural variant analysis and benchmarking using CHM13-T2T. Genome Res. Cold Spring Harbor Laboratory; 35:621–312025;

em Editorial Manager®

Cookie Preference Center

We use cookies which are necessary to make our site work. We may also use additional cookies to analyze, improve and personalize our content and your digital experience. For more information, see our Cookie Policy.

You may choose not to allow some types of cookies. However, blocking some types may impact your experience of our site and the services we are able to offer. See the different category headings below to find out more or change your settings.

Allow all

Manage Consent Preferences

Strictly Necessary Cookies

Always active

These cookies are necessary for the website to function and cannot be switched off in our systems. They are usually only set in response to actions made by you which amount to a request for services, such as setting your privacy preferences, logging in or

filling in forms. You can set your browser to block or alert you about these cookies, but some parts of the site will not then work.

Functional Cookies

These cookies enable the website to provide enhanced functionality and personalisation. They may be set by us or by third party providers whose services we have added to our pages. If you do not allow these cookies then some or all of these services may not function properly.

Performance Cookies

These cookies allow us to count visits and traffic sources so we can measure and improve the performance of our site. They help us to know which pages are the most and least popular and see how visitors move around the site.

Targeting Cookies

These cookies may be set through our site by our advertising partners. They may be used by those companies to build a profile of your interests and show you relevant adverts on other sites. If you do not allow these cookies, you will experience less targeted advertising.

Cookie List

Clear

Apply Cancel
Consent Leg.Interest

Confirm my choices

Power
