# Supplementary material for: Cancer genome standards for long-read sequencing using cancer cell line mixtures
Source: Gigascience. 2026 Apr 3;15:giag037. doi: 10.1093/gigascience/giag037 (PMC13137868; doi:10.1093/gigascience/giag037)
Supplement: giag037_Reviewer_1_Report_original_submission — Reviewer 1 -- 7/3/2025 [file giag037_reviewer_1_report_original_submission.pdf]

Reviewer Report

Title: Cancer genome standards for long-read sequencing using cancer cell line mixtures

Version: Original Submission **Date:** 7/3/2025

Reviewer name: Reviewer 1

Reviewer Comments to Author:

The study measured how somatic variant classification with long-read sequencing (LRS) technology was affected by data quality: tumor sample purity and read depth. Various types of somatic variants (single nucleotide variants [SNV]s, indels, and structural variants [SV]s) called by LRS were compared to a reasonable short-read sequencing (SRS) gold standard. Evaluation was primarily done by quantifying the change in classification metrics (precision and recall) as tumor purity and sequencing depth decreased. The main impacts of the work were 1) showed comprehensive, empirical results of which values of purity and depth maintain reasonable recall and precision of somatic variants, 2) showed how length and genomic location of variants support the hypothesis that LRS false positives may be variants missed by SRS standard, and 3) provided insights toward how changes in precision and recall are nuanced across different variant calling methods. In addition, public release of the variable purity and sequencing depth dataset would enable further research into how data quality impacts somatic variant calling.

Major comments

Strengths

1. The impact of the work included 1) informing data quality standards for somatic variant analysis, 2) hypothesizing somatic variants distinct to LRS methods and missed by SRS methods, and 3) a nuanced evaluation of various somatic variant calling tools in different data quality scenarios.
2. The analysis was novel compared to previous germline variant LRS benchmarks since, here, the experiments evaluated data quality aspects distinct to cancer datasets: tumor purity and sequencing depth of paired tumor and normal samples.
3. Interpretation of experimental results was mostly correct. And, the discussion section accurately summarized key findings regarding the effect of tumor purity and sequencing depth on somatic variant calling.

Weakness

1. The lack of analysis for overlapping true positives or false positives across methods was a missed opportunity to understand the potential impact of ensemble methods. Albeit, an analysis of overlapping calls is slightly out of scope of this work. Therefore, I do not see overlap analysis as a necessary revision.
2. The argument for filtering ClairS by a singular variant statistic should be cautioned by the implied decrease in recall when applying filters. From the results figures, it's unclear whether any threshold could be applied for a singular variant statistic (e.g., sequencing depth, mapping quality, or variant allele frequency) without a significant reduction in recall. A safer argument is to filter by a combination of these statistics.

Minor comments

Interpretation of SF16 results seems incorrect. The majority of LRS FPs overlap with high/low GC content

regions for most methods, not low mappability regions. This argument is mentioned on Pg 9, sec 2.4, paragraph 2, lines 4-5.
Regarding the Figure 5A analysis, I do not understand how SBS vectors are set up for cosine similarity measurement. Please make it clear how SBS vectors are set-up/aggregated from variants. Also, a baseline reference of the expected cosine similarity at random would make this result more impactful and interpretable.
The results of supplementary figure (SF) 3 seemed unrealistic to me. Specifically, I am surprised that high/low methylation always gets reversed as a function of purity. Please provide some intuition for why this result is (un)-expected, and double-check this result.
When reading the paper top-down, it is confusing why the definitions of FP, TP, and FN were switched to LRS variants, overlapping variants and SRS variants in SF7. The analysis of SF7 preceded the evidence of LRS FPs being missed by the SRS standard shown later in Fig 5. Overall, the foreshadowing of LRS FPs being missed calls made me skeptical until there was evidence supporting this hypothesis later in the text.
The supplementary figure 8 legend is incorrect. Column and row descriptions do not match the figure. And, what does DP stand for?
A direct quantification, such as the difference of mean recalls across methods, is missing between SRS standard and secondary callset.
The abstract argues that LRS better aligns to "complex" regions (abstract, line 2). The intro discusses how SRS is bad in low complexity regions (introduction, lines 7&8) … later in the intro, LRS is again discussed as effectively "spanning complex genomic regions" (introduction, line 11). I believe the argument attempting to be made is that LRS better aligns to low complexity regions than SRS. Please make this argument consistent and clear.
For analysis of SF10 in section 2.3, the consistently high precision of nanomonsv seems worth mentioning. This is mentioned in the discussion, but I think it should be mentioned in the results section, too.
It would be helpful to indicate which stains are telomere and centromere regions in supplementary figure 11-14 keys.
"N50 is" -> "N50 was" (results, paragraph 1, last line)
Make capitalization consistent for the sub-figure character labels in supplementary figure 1.
At section 2.2, paragraph 5, line 2 change plural "indels" to singular "indel"
Discussion, p14, para 8, line 7, "on" -> "one"
Conf of interest, P20, l1, "Nanopre" -> "Nanopo

Methods

Are the methods appropriate to the aims of the study, are they well described, and are necessary controls included? Choose an item.

Conclusions

Are the conclusions adequately supported by the data shown? Choose an item.

Reporting Standards

Does the manuscript adhere to the journal's guidelines on minimum standards of reporting? Choose an item.

Choose an item.

Statistics

Are you able to assess all statistics in the manuscript, including the appropriateness of statistical tests used? Choose an item.

Quality of Written English

Please indicate the quality of language in the manuscript: Choose an item.

Declaration of Competing Interests

Please complete a declaration of competing interests, considering the following questions:

- Have you in the past five years received reimbursements, fees, funding, or salary from an organisation that may in any way gain or lose financially from the publication of this manuscript, either now or in the future?
- Do you hold any stocks or shares in an organisation that may in any way gain or lose financially from the publication of this manuscript, either now or in the future?
- Do you hold or are you currently applying for any patents relating to the content of the manuscript?
- Have you received reimbursements, fees, funding, or salary from an organization that holds or has applied for patents relating to the content of the manuscript?
- Do you have any other financial competing interests?
- Do you have any non-financial competing interests in relation to this paper?

If you can answer no to all of the above, write 'I declare that I have no competing interests' below. If your reply is yes to any, please give details below.

I declare that I have no competing interests.

I agree to the open peer review policy of the journal. I understand that my name will be included on my report to the authors and, if the manuscript is accepted for publication, my named report including any attachments I upload will be posted on the website along with the authors' responses. I agree for my report to be made available under an Open Access Creative Commons CC-BY license (http://creativecommons.org/licenses/by/4.0/). I understand that any comments which I do not wish to be included in my named report can be included as confidential comments to the editors, which will not be published.

Choose an item.

To further support our reviewers, we have joined with Publons, where you can gain additional credit to further highlight your hard work (see: https://publons.com/journal/530/gigascience). On publication of this paper, your review will be automatically added to Publons, you can then choose whether or not to claim your Publons credit. I understand this statement.

Yes Choose an item.
