# Supplementary material for: Cancer genome standards for long-read sequencing using cancer cell line mixtures
Source: Gigascience. 2026 Apr 3;15:giag037. doi: 10.1093/gigascience/giag037 (PMC13137868; doi:10.1093/gigascience/giag037)
Supplement: giag037_Reviewer_2_Report_original_submission — Reviewer 2 -- 7/31/2025 [file giag037_reviewer_2_report_original_submission.pdf]

Reviewer Report

Title: Cancer genome standards for long-read sequencing using cancer cell line mixtures

Version: Original Submission **Date:** 7/31/2025

Reviewer name: Reviewer 2

Reviewer Comments to Author:

This study assesses the accuracy of long-read sequencing with Oxford Nanopore Technology (ONT) for detecting somatic variants in tumor cell line mixtures, offering valuable insights for optimizing cancer genomic analyses, particularly regarding sequencing depth, tumor purity, and comparisons with short-read technologies. Overall, the manuscript is well-written, clearly structured, and presents significant findings in a highly relevant area of research.

However, the code repository for this study (https://github.com/bakeronit/nanopore_celllines_benchmark) is currently unavailable. It is unclear whether the repository is private or has been renamed. Considering that GigaScience strongly emphasizes reproducibility, the inability to access and verify the repository significantly limits this review.

The manuscript mentions the use of downsampling to synthetically reduce coverage depth, but upsampling-based simulations were not explored. I strongly recommend including synthetic datasets, as they can substantially enhance both experimental design and result validation. For example: PBSIM2 for PacBio and ONT (https://github.com/yukiteruono/pbsim2), NanoSim for ONT (https://github.com/bcgsc/NanoSim). Such simulations offer several advantages: Validation of the reconstruction methodology; Full control over somatic variants (gold standard); Tunable sequencing coverage; Exploration of extreme scenarios; Cross-validation of real data (false positives/negatives); Systematic technology comparisons; Benchmarking against real datasets. Similarly, short-read simulations could be performed using ART (https://www.niehs.nih.gov/research/resources/software/biostatistics/art) or art_modern (https://github.com/YU-Zhejian/art_modern).

Sequencing technology strongly impacts the interpretation of long-read studies, as differences in error profiles, read length, required depth, and compatible tools directly influence sensitivity, precision, and the types of variants that can be reliably detected. This study exclusively reports ONT-based results, and some conclusions may not generalize to other long-read platforms. It would be more informative to explicitly mention the sequencing technology in the abstract and possibly in the article title.

The use of GRCh38 is suboptimal when compared to CHM13-T2T, which resolves previously unresolved repetitive, telomeric, and centromeric regions-areas that the study itself identifies as relevant to accuracy. Incorporating CHM13-T2T in the current analysis could significantly strengthen the study, and would also make Supplementary Figures 11-14 more informative.

Including metrics that better capture imbalanced variant detection performance, such as AUROC and AUPRC, would provide a more comprehensive assessment of the methods.

Minor Comments

In the sentence: "Two approaches were used for somatic SNVs and indels detection from long-read data. ClairS [32] and DeepSomatic [31]." - consider replacing "." with a ":".

Methods

Are the methods appropriate to the aims of the study, are they well described, and are necessary controls included? Choose an item.

Conclusions

Are the conclusions adequately supported by the data shown? Choose an item.

Reporting Standards

Does the manuscript adhere to the journal's guidelines on minimum standards of reporting? Choose an item.

Choose an item.

Statistics

Are you able to assess all statistics in the manuscript, including the appropriateness of statistical tests used? Choose an item.

Quality of Written English

Please indicate the quality of language in the manuscript: Choose an item.

Declaration of Competing Interests

Please complete a declaration of competing interests, considering the following questions:

- Have you in the past five years received reimbursements, fees, funding, or salary from an organisation that may in any way gain or lose financially from the publication of this manuscript, either now or in the future?
- Do you hold any stocks or shares in an organisation that may in any way gain or lose financially from the publication of this manuscript, either now or in the future?
- Do you hold or are you currently applying for any patents relating to the content of the manuscript?
- Have you received reimbursements, fees, funding, or salary from an organization that holds or has applied for patents relating to the content of the manuscript?
- Do you have any other financial competing interests?
- Do you have any non-financial competing interests in relation to this paper?

If you can answer no to all of the above, write 'I declare that I have no competing interests' below. If your reply is yes to any, please give details below.

I declare that I have no competing interests

I agree to the open peer review policy of the journal. I understand that my name will be included on my report to the authors and, if the manuscript is accepted for publication, my named report including any attachments I upload will be posted on the website along with the authors' responses. I agree for my report to be made available under an Open Access Creative Commons CC-BY license (http://creativecommons.org/licenses/by/4.0/). I understand that any comments which I do not wish to be included in my named report can be included as confidential comments to the editors, which will not be published.

Choose an item.

To further support our reviewers, we have joined with Publons, where you can gain additional credit to further highlight your hard work (see: https://publons.com/journal/530/gigascience). On publication of this paper, your review will be automatically added to Publons, you can then choose whether or not to claim your Publons credit. I understand this statement.

Yes Choose an item.
